# Pollen precedence in sexual *Potentilla puberula* and its role as a protective reproductive barrier against apomictic cytotypes

**DOI:** 10.12705/676.9

**Published:** 2018-12

**Authors:** Henar Alonso-Marcos, Karl Hülber, Tuuli Myllynen, Patricia Pérez Rodríguez, Christoph Dobeš

**Affiliations:** 1 Department of Forest Genetics, Austrian Research Centre for Forests, Seckendorf-Gudent-Weg 8, 1131 Vienna, Austria; 2 Department of Conservation Biology, Vegetation Ecology and Landscape Ecology, University of Vienna, Rennweg 14, 1030 Vienna, Austria

**Keywords:** apomixis, Eastern European Alps, flow cytometric seed screen, pollen precedence, reproductive isolation, Rosaceae

## Abstract

Cross-pollination is a major factor determining the demographic dynamics of mixed-ploidy populations. Typically, rare cytotypes are suppressed due to reduced female fertility by losing gametes in heteroploid crosses (i.e., through minority cytotype exclusion). In species with reproductive differentiation into sexual and apomictic cytotypes, sexuals might be reproductively suppressed by apomicts (or transformed due to introgression of apomixis genes). Pollen precedence potentially acts as a post-pollination pre-fertilization barrier protecting sexuals against their apomictic counterparts. We estimated the role of pollen precedence as a barrier against cross-fertilization of tetraploid sexuals by penta- and heptaploid gametophytic apomicts in *Potentilla puberula* (Rosaceae) by means of controlled crosses, and inference of the paternity through DNA ploidy estimation of embryos. Individuals from five regions spanning an elevational and biogeographic gradient were used to account for the variation in the relative frequencies of reproductive modes across the study area. We tested (1) whether the application of heteroploid pollen (sexual × apomictic) causes a reduction of seed yield compared to homoploid crosses (sexual × sexual), and (2) if so, whether pollen precedence recovers the seed yield in simultaneous applications of pollen from sexuals and apomicts (mixed-ploidy). Seed yield was significantly lower in hetero- than in homoploid crosses. We found clear evidence for precedence of homoploid pollen, despite a 13% to 15% of embryos experienced a change in ploidy due to heteroploid fertilizations. Thus, our study indicates that pollen precedence operates as a barrier against intercytotype fertilization in *P. puberula*, promoting the integrity of the sexual cytotype and their co-existence with apomictic individuals.

## Introduction

Reproductive isolation is fundamental to maintain the integrity of diverged evolutionary lineages of plants. In sympatry, sequential mechanisms operate to restrict gene flow and, hence, limit hybrid formation. Post-zygotic barriers thereby decrease the reproductive potential of populations via abortion or reduced viability of the progeny due to genic or genomic incompatibilities (Dobzhansky, 1936; Muller, 1932). In contrast, pre-zygotic barriers act against the formation of a hybrid zygote. They include spatial and microhabitat differentiation (Hülber & al., 2009; Sonnleitner & al., 2010), temporal isolation via asynchronous flowering time (Petit & al., 1997; Lamont & al., 2003; Botes & al., 2008), differential flower architecture (Grant, 1994; Kay, 2006) leading to assortative pollinator behaviour (Husband & Schemske, 2000; Lowry & al., 2008), and pollen precedence (Howard, 1999). These reproductive barriers act in concert to establish and strengthen genetic boundaries.

Pollen precedence is defined as differential fertilization success of pollen from different species upon their simultaneous deposition on a recipient’s stigma(s) (Howard, 1999). Reduced germination of pollen on the stigma and/or slower pollen tube growth of foreign compared to conspecific pollen limit or avoid hybridization despite cross-pollination (Campbell & al., 2003; Chapman & al., 2005). Conspecific pollen precedence thus has been identified as an important component of reproductive isolation in closely related species (Arnold & al., 1993; Rieseberg & al., 1995; Brock, 2009; Abadie & al., 2012; Lepais & al., 2013; Brys & al., 2014). However, competitive interactions among pollen might also occur at the intraspecific level like in systems exhibiting cytological differentiation by ploidy. Whole-genome duplication events, or polyploidization, occur either due to genome duplication within a species (autopolyploid) or hybridization of two species (allopolyploid). Intraspecific pollen precedence was indeed demonstrated for species showing differentiation by ploidy as di- and tetraploid individuals of *Chamerion angustifolium* (L.) Holub (Husband & al., 2002) and *Ranunculus adoneus* A.Gray (Baack, 2005). Polyploidization is considered as a common driver of lineage divergence, deducible as ancient events preceding angiosperm radiation, and in more recent time scales, from increased diversification rates in periods of environmental instability (Stebbins, 1947; Grant, 1981; Otto & Whitton, 2000; Comai, 2005; Soltis & al., 2007; Baker & al., 2016; Soltis & Soltis, 2016; Landis & al., 2018). However, maintenance and divergence of novel ploidy differentiated lineages depends on their reproductive isolation (Kolár & al., 2017).

In the absence of complete prezygotic reproductive isolation, difference in ploidy among mating partners typically leads to a disturbed development or even the abortion of the progeny (Lin, 1984; Koltunow & Grossniklaus, 2003). This is because heteroploid fertilizations cause loss of zygotes or later ontological stages (Coyne & Orr, 1998; Brown & Mitchell, 2001; Hersh & al., 2016) and can constrain the maintenance or co-occurrence of cytotypes through the minority cytotype exclusion principle (Levin, 1975). A specific situation arises in sexual-apomictic systems in which ploidy differentiation is usually accompanied by a differentiated reproductive mode. Typically, diploid (or low-ploidy) cytotypes are sexual while higher-ploidy individuals are apomictic (Bayer, 1997; Hojsgaard & al., 2008; Cosendai & al., 2011). In contrast to the sexuals, the embryos of apomicts develop autonomously without fertilization (Nogler, 1984; Asker & Jerling, 1992; Hörandl & Hojsgaard, 2012), effectively counteracting loss of zygotes. This asexual embryo development in apomictic individuals may lead to asymmetric reproductive interference in mixed-cytotype populations (e.g., Hersh & al., 2016; Dobeš & al., 2018) because by expectation, only sexual individuals suffer from reduced fitness of progeny through heteroploid fertilizations. Furthermore, apomixis might introgress as a heritable trait (Ozias-Akins & Van Dijk, 2007), and reproductively transform sexuals (Joshi & Moody, 1995, 1998).

In this study, we investigated the role of pollen precedence as a barrier against fertilization of sexuals by apomictic ploidy cytotypes in *Potentilla puberula* (Rosaceae). We applied homo- and heteroploid pollen as well as a pollen mixture containing both types of pollen (mixed-ploidy pollination) onto the stigmas of emasculated flowers of sexuals in a controlled ex situ crossing experiment. Furthermore, we estimated and quantified, based on the ploidy of embryos, the paternity of seeds derived from the mixed-ploidy pollinations using flow cytometric seed screen (FCSS). By this approach, we verified the following expectations: (1) Heteroploid pollinations cause a reduction in seed yield compared to homoploid pollinations; and, if so, (2) homoploid pollen precedence is a means to avoid or reduce such losses of progeny in mixed-ploidy pollinations.

## Materials and Methods

### The study species

The rosaceous species *Potentilla puberula* Krašan (= *Potentilla pusilla* Host; Soják, 2010) is a perennial herb distributed in the European Alps and the Carpathians (Kurtto & al., 2004). The yellow pentamerous insect-pollinated flowers are polyandrous and present more than one ovule – on average 28 ± 9.7 ovules (N = 251 individuals) – with each of the ovaries giving rise to a one-seeded fruitlet (for convenience, we consistently use the term seed when referring to both, fruitlets and isolated seeds as used in the FCSS). The species exhibits a series of ploidy levels comprising tetra- (*x* = 7; 2*n* = 28), penta- (2*n* = 35), hexa- (2*n* = 42), hepta- (2*n* = 49), octo- (2*n* = 56), and occasionally nonaploids (2*n* = 63) (Dobeš, 1999). Tetraploid individuals are almost exclusively sexual and self-incompatible, whereas higher polyploids are self-compatible and predominantly apomictic. Apomixis is gametophytic and pseudogamous; i.e., fertilization is still required for endosperm development and successful seed formation (Dobeš & al., 2013, 2018). Reproductive modes are ecologically and biogeographically differentiated (Nardi & al., 2018): tetraploids preferentially inhabit pristine sites in areas that likely served as a glacial refugium (Tribsch & Schönswetter, 2003), while higher ploids prefer secondary habitats and higher elevations in formerly glaciated areas. However, intermixture at the population level occurs regularly (in 60 out of 269 sites tetraploids co-occurred with higher ploids in a screen within an area approximately congruent to the study area. as described in the following (Hülber & al., 2013; Nardi & al., 2018).

### Study area and plant material

The study is based on plants collected during spring 2016 from five regions spanning an elevational and biogeographic gradient, which largely covers the ecological range realized by the species. Within each region, sexual and apomictic individuals grow at similar frequencies. Three of the five studied regions were inhabited by tetra- and pentaploid individuals: Zabernig (47°00′16.812″ N / 12°31′09.119″ E, at 1340 m a.s.l.) and Obersteiner (47°01′04.33″ N / 12°24′31.39″ E, at 1460 m a.s.l.), both located in East Tyrol, Austria, and Bodenalm (46°55′23.124″ N / 11°41′53.195″ E, at 1700 m a.s.l.) in South Tyrol, Italy. Each of the two remaining regions of the study comprise in turn two similar and spatially close sites: with tetraploids in the first site, and both penta- and heptaploids in the second site. This way, Raas with tetraploids (46°44′49.49″ N / 11°39′12.60″ E, at 750 m a.s.l.) was combined with penta- and heptaploids from Fortezza (46°46′35.58″ N / 11°37′47.46″ E, at 730 m a.s.l.), in South Tyrol, Italy, whereas Ossenigo with tetraploids (45°40′27.95″ N / 10°54′34.16″ E, at 250 m a.s.l.) was combined with Scaiola penta- and heptaploid individuals (45°32′03.26″ N / 10°21′50.43″ E, at 200 m a.s.l.), Trentino/Lombardia, Italy.

### Homoploid, heteroploid and mixed-ploidy crossing experiment

A controlled ex situ crossing experiment was carried out from April to June 2017 in the experimental garden of the Austrian Research Centre for Forests (https://bfw.ac.at/), located in the grounds of the Schönbrunn Palace Park in Vienna (48°10′36″ N / 16°18′34″ E at 259 m a.s.l.). Plants were grown from cuttings collected in the field in 2016, in 14 cm pots. Out of the plants, we randomly selected from each of the five regions 12 individuals as pollen recipients and 190 plants as pollen donors. More specifically, sexual and apomictic pollen donors were used per each region, respectively, as follows: Zabernig 8/11; Obersteiner 16/13; Ossenigo/Scaiola 14/43; Raas/Fortezza 16/39 and Bodenalm 18/12, in the homoploid and the heteroploid treatment, respectively. Plants were planted in plots spaced in minimum 10 meters from each other. Prior to pollination, flowers were carefully emasculated to prevent self-fertilization and bagged using bridal veil, as this material has the least effect on the microclimate of the bagged flowers (Wyatt & al., 1992). At stigma maturity, flowers were pollinated by gently rubbing the anthers of pollen donors over the pollen recipients’ stigmas. Four treatments were applied: (i) Homoploid pollinations among sexuals: each of three flowers per individual was pollinated with pollen from one randomly selected donor of the same (tetraploid) ploidy; (ii) Heteroploid pollinations of sexuals by apomicts: three flowers (but six at sampling sites harboring not only pentaploid but also heptaploids) were treated with pollen of a heteroploid donor; and (iii) Mixed-ploidy pollinations: six flowers per recipient (but twelve at sampling sites including penta- and heptaploids) were pollinated with both, pollen from one sexual and one apomictic donor. In the latter case, we applied the pollen by rubbing the anthers of both donors over the recipients’ stigmas. Application of about equal amounts of pollen from both pollen donors was secured by visual inspection of stigmas using a binocular. To avoid bias due to the order of pollination, we alternated sexual versus apomictic pollen donors when starting a pollination (Electr. Suppl.: Table S1). In addition, (iv) two flowers per pollen recipient served as open control. At maturity, reproductive success was determined as the number of obtained viable seeds (i.e., filled with a fleshy embryo). We used seed yield as a measure of reproductive success, since no relationship between number of ovules and number of mature seeds was found in previous crossing experiment in *P. puberula* (Dobeš & al., 2018). The estimation of the number of ovules (required for defining the seed set) is destructive in our model system (Dobeš & al., 2018). Thus, by using seed yield, we maximized the number of flowers available for pollination. Moreover, effects of the variation in the number of ovules among individuals were removed by performing all treatments on every pollen recipient. Furthermore, seed yield has been used in other crossing experiments (e.g., Behrend & al., 2015; Sutherland & Galloway, 2017), including *Potentilla* (Trunschke & Stöcklin, 2017).

Data derived from crosses with penta- and heptaploid pollen donors were pooled for each treatment, since penta- and heptaploid individuals did not show differences in their behaviour among treatments and considered in the following together as apomicts (Electr. Suppl.: Table S2).

To compare the number of seeds (i.e., seed yield) among treatments, Poisson Generalized Linear Mixed Models (GLMMs), as implemented in the package glmmTMB v.0.2.0 (Magnusson & al., 2017) of the R statistical environment (R Development Core Team 2017), were applied. These models comprise a conditional and a zero-inflated model component (Brooks & al., 2017). Treatment was used as the fixed-effects predictor in the conditional model component, and random intercepts for each maternal and paternal individual, both nested in population, were included to account for potential differences in pollination success among individual pollen recipients and pollen donors. Because many flowers produced no viable seed (in all treatments, including the open control), we allowed for random intercepts in the zero-inflation component. We compared (i) the homo- against the heteroploid treatment to test for confounding effects of interploidy fertilization on seed yield, as well as (ii) the pooled data of these two against the mixed-ploidy treatment by using Helmert contrasts. This comparison was done in order to test for the existence of pollen precedence, i.e., if homo- and heteroploid pollen have an unequal probability to fertilize an ovule, resulting in a seed yield not intermediate to the mixed-ploidy treatment. (iii) We compared seed yield in the mixed-ploidy treatment (as the baseline) to that in the homoploid one, as well as to the heteroploid treatment in order to test whether, at least, partial recovery due to the pollen precedence from crosses with heteroploid pollen occurs. These comparisons among treatments were also applied separately to the data of each region to examine whether the detected pattern is idiosyncratic or generic.

To test whether pollen quality affected seed formation, the number of vital pollen grains (i.e., in the sense of stainable in a physiological vitality test) per flower was estimated for 157 pollen donors. Two anthers per individual were stained with a solution of Malachite green, acid fuchsin and Orange G for at least 12 hours (Peterson & al., 2010, vitality stain) and diluted in 50 μl of distilled water. A homogenized 2 μl aliquot was analyzed using the light microscope Axioskop 1 (Zeiss, Jena, Germany) and bright-field illumination at 400-fold magnification. Stained and regular-shaped (i.e., physiologically and morphologically intact) grains were considered as viable and counted (Electr. Suppl.: Fig. S1). The number of obtained pollen grains were compared among treatments using zero-inflated GLMMs as described above, but assuming pollen quality to be a Gaussian-distributed response variable.

### Flow cytometry seed screen

The ploidy of functional (successful in heteroploid crosses) male gametes of penta- and heptaploids was 1.6-times (ca. corresponding to triploidy) and 1.9-times (ca. tetraploid), respectively, higher than those of sexual tetraploids (Dobeš & al., 2018). Thus, embryos derived from heteroploid crosses (fertilization with pollen from penta- and heptaploid donor plants) are expected to have a higher ploidy than those obtained in homoploid crosses (tetraploid donors). To estimate the proportions of progeny sired by sexual (i.e., homoploid) and apomictic (i.e., heteroploid) pollen donors, we compared the embryo-to-standard fluorescence ratio of each seed derived from the mixed-ploidy crosses to the distribution of such ratios obtained from the following reference samples: Thirty-five seeds (sampled from 13 individuals) drawn from crosses with tetraploid pollen donors were used as a homoploid reference sample. As heteroploid reference samples, 37 seeds (from 17 individuals) and 30 seeds (from 7 individuals) obtained in crosses with pentaploid and heptaploid pollen donors were used, respectively. Seeds of the mixed-ploidy crosses with embryo-to-standard fluorescence ratios below the lower limit of the 95% confidence interval (i.e., the 2.5% confidence limit computed as arithmetic mean − 1.96 × standard deviation) of the distribution of embryo-to-standard fluorescence ratios obtained from (each of) the heteroploid reference sample, were regarded as emerged from a homoploid fertilization. Accordingly, embryo-to-standard fluorescence ratios above the upper limit of the 95% confidence interval (i.e., the 97.5% limit) of the homoploid reference sample identifies seeds originated by heteroploid fertilisation. Calculations were done separately for pentaploid and heptaploid pollen donors. Normality distribution of the values observed for the homo- and heteroploid references was tested with the Shapiro-Wilk test.

Then, from the mixed-ploidy treatment, 1001 randomly selected seeds were analysed using FCSS (Matzk & al., 2000) from all the five studied regions. The two-steps protocol followed Doležel & al. (2007) and modifications by Dobeš & al. (2013). In brief, seeds were analysed using Otto I + II buffers (Otto, 1990), DAPI (4′-6-diamidino-2-phenylindole) as DNA-specific fluorescence stain and *Pisum sativum* ‘Kleine Rheinländerin’ as internal biological standard (Greilhuber & Ebert, 1994). First, fleshy seeds freed from the fruit wall/testa were chopped together with the internal standard in 200 μl Otto I extraction buffer using a razor blade in a petri dish, and 200 μl more were added before placing the sample on ice for 30 minutes. Subsequently, the petri dishes were ultrasonicated for 1 minute and then filtered through a 20 μm nylon mesh filter (Partec CellTrics, Partec Münster, Germany). Prior to the measurements, 1.2 ml Otto II containing 0.2 μg DAPI/ml was added to the samples. After five minutes, samples were measured with a Partec ML Ploidy Analyser. The embryo to standard fluorescence ratio was calculated from the means of the fluorescence histograms using FloMax (v.2.9, Quantum Analysis, 2014).

## Results

### Crossing experiment

**Fig. 1:**
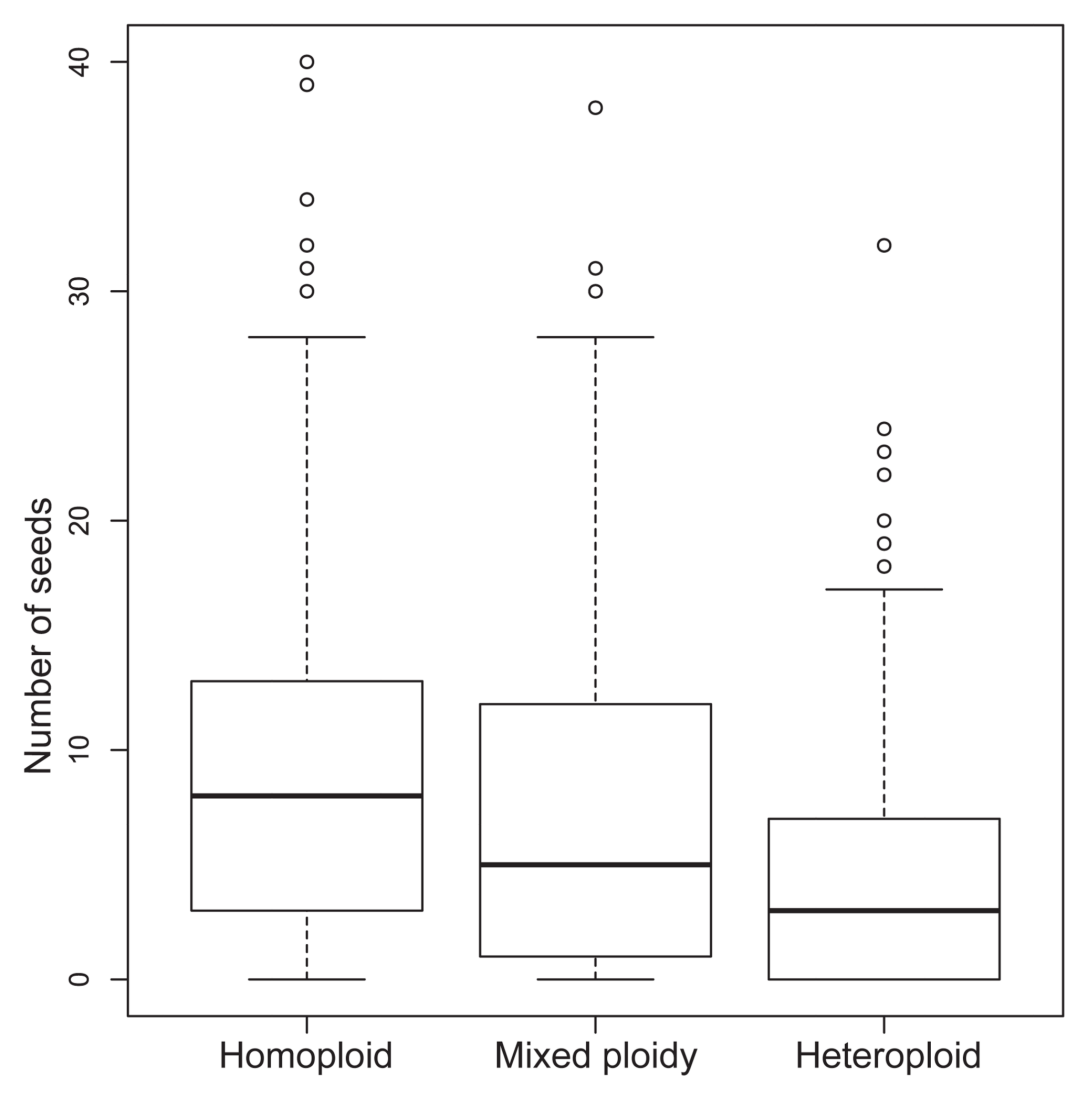
Seed yield of 60 sexual tetraploid *Potentilla puberula* Krašan individuals crossed with pollen derived from sexual (i.e., homoploid) and apomictic (i.e., heteroploid) donor plants as well as with a mixture of both pollen types. Significant differences (*p* < 0.001) were found for the comparison among all treatments.

A total of 638 pollinations were carried out for the three treatments. At maturity, 1410 seeds (146 pollinations, mean ± SD 9.65 ± 8.77) were obtained for the homoploid treatment, 930 seeds (193 pollinations, 4.89 ± 5.94) for the heteroploid treatment and 2124 seeds (299 pollinations, 7.1 ± 7.26) for the mixed-ploidy treatment (Table 1). Pooled over the five regions, seed yield obtained in the homoploid treatment was significantly higher than in the heteroploid treatment (Fig. 1; zero-inflated GLMM: coefficient ± SE: −0.33 ± 0.04, *z* = −7.83, *p* < 0.001). The seed yield derived from the mixed-ploidy treatment was significantly lower (0.31 ± 0.08, *z* = 3.73, *p* < 0.001) and higher (−0.35 ± 0.08, *z* = −4.34, *p* < 0.001) than the homoploid and heteroploid treatment, respectively, but did not significantly differ from the pooled data of these two treatments (0.01 ± 0.02, z = 0.33, *p* = 0.781). These patterns of seed yield (homoploid > mixed-ploidy > heteroploid but mixed-ploidy = pooled homoploid + heteroploid) were confirmed for most of the five studied regions when tested separately (Table 2).

The mean number of vital pollen grains per sample (Electr. Suppl.: Table S3) was 522.30 ± 330.64 SD for the tetraploids (range 69 to 1415), 347.72 ± 209.37 for the pentaploids (64 to 892) and 234.25 ± 219.68 for the heptaploids (1 to 921). Pollen quality decreased in tendency with increasing ploidy: tetraploids 94.59 ± 7.15% (71.45%–100%), pentaploids 90.76 ± 9.56% (56.49%–100%) and heptaploids 86.24% ± 14.08% (35.37%–100%). Pollen quality differed among reproductive modes, but did not significantly affect the number of obtained seeds neither in the homoploid (zero-inflated GLMM: coefficient ± SE: 0.02 ± 0.13, *z* = 0.15, *p* = 0.878) nor in the heteroploid (0.04 ± 0.04, *z* = 1.12, *p* = 0.263) or the mixed-ploidy treatment (−0.04 ± 0.06, *z* = −0.67, *p* = 0.506).

### Flow cytometric seed screen

**Fig. 2:**
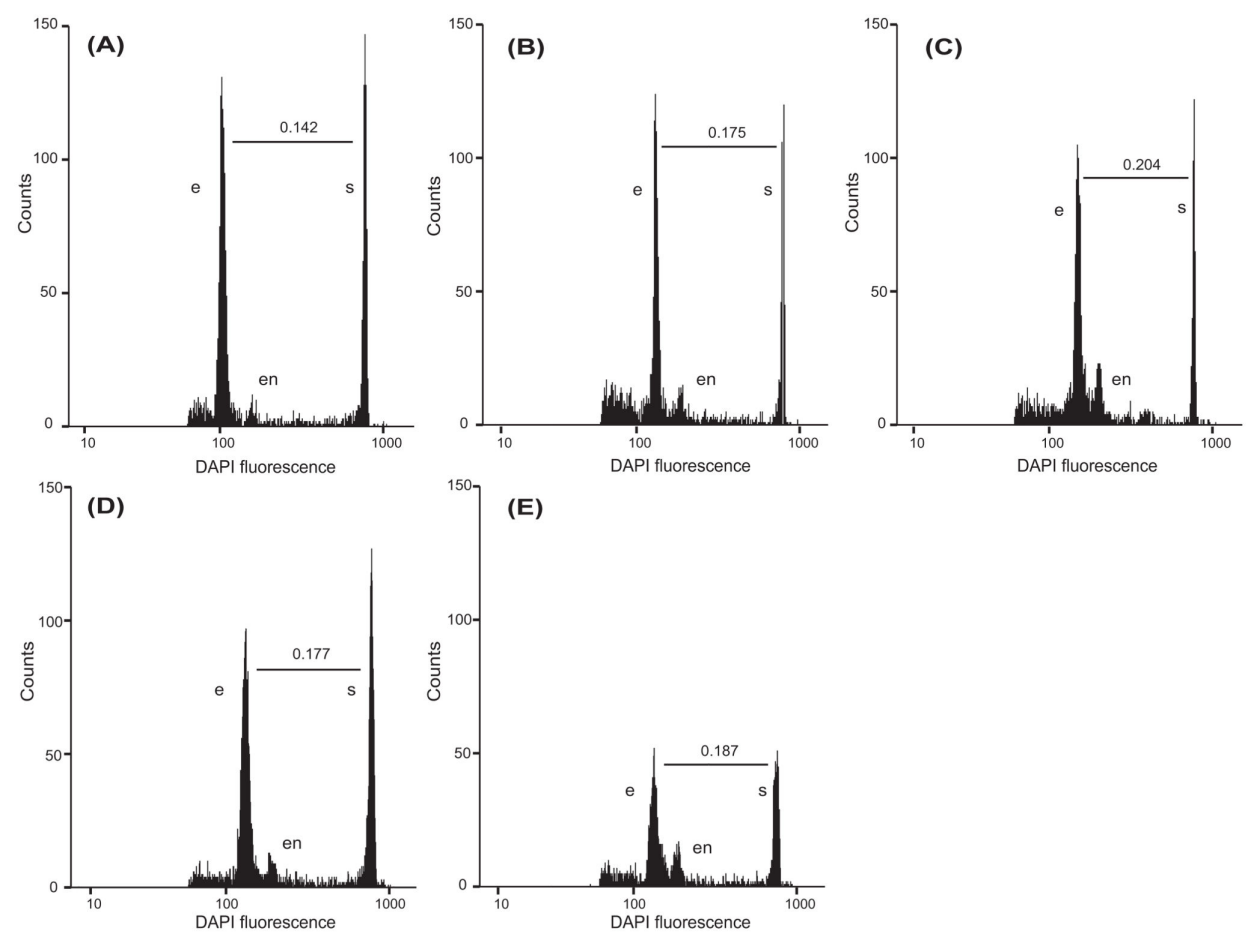
Flow cytometric sample histograms for *Potentilla puberula* Krašan seeds obtained from sexual tetraploid mother plants crossed with **(A)** a tetraploid sexual father, **(B)** a pentaploid apomictic father, **(C)** a heptaploid apomictic father, **(D)** a mixture of pollen of tetra- and pentaploid fathers, and **(E)** a mixture of pollen of tetra- and heptaploid fathers. Letters indicate the signals for the embryo (e), the endosperm (en) and the internal standard *Pisum sativum* (s). The embryo-to-standard fluorescence ratio was calculated for each seed. Indicated ratios of 0.142, 0.175 and 0.204 are representative for a pollination with (A) DNA-tetraploid, (B) DNA-pentaploid and (C) DNA-hexaploid donor plants. Accordingly, embryos in (d, e) are of heteroploid origin.

Good-quality fluorescence signals were obtained for 871 seeds out of the 1001 analysed from the mixed-ploidy treatment. More precisely, the number of screened seeds per region were 15, 183, 336, 134 and 203 from Zabernig, Obersteiner, Raas/Fortezza, Ossenigo/Scaiola and Bodenalm, respectively (Electr. Suppl.: Table S4). The number of embryo nuclei counted per sample and the coefficient of variation (CV) of the peaks ranged between 155 and 6367 (mean ± SD 1406 ± 821) and 3.32–8.22 (mean ± SD 5.22 ± 0.76), respectively. Examples of flow cytometric histograms are shown in Fig. 2 for homoploid, heteroploid and mixed-ploidy analysed embryos.

**Fig. 3:**
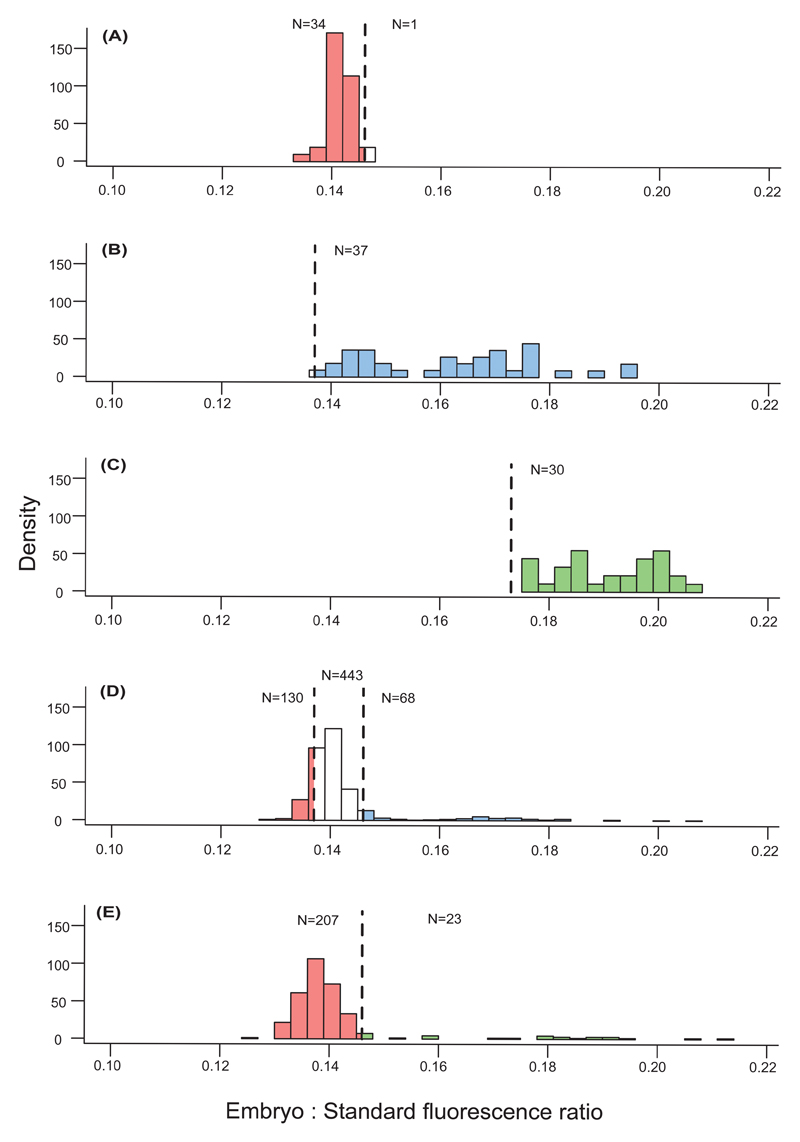
Frequency distributions of embryo-to-standard fluorescence ratios observed for *Potentilla puberula* seeds obtained in fertilizations of tetraploid sexual mothers with **(A)** homoploid sexual tetraploid pollen donors **(B)** with pentaploid apomictic pollen donors, **(C)** with heptaploid apomictic pollen donors, **(D)** with a mixture of pollen from tetra- and pentaploid donors, and **(E)** with a mixture of pollen from tetra- and heptaploid donors. The three reference samples (A–C) were used to infer the paternity of seeds obtained from the mixed-ploidy pollinations. To differentiate between fertilizations by sexuals and apomicts, the upper (i.e., 97.5%) and lower (i.e., 2.5%) limit of the 95% confidence interval (given as dashed lines) of the homoploid and each of the two heteroploid reference samples, respectively, served as thresholds. Seed with a ploidy below (red bars) and above (blue and green bars) these limits were inferred as of homo- and heteroploid origin, respectively. In contrast, seeds having a ploidy in between the limits (white bars) could not be unambiguously inferred.

In the homoploid reference sample, we observed a single peak of embryo-to-standard fluorescence ratios (Electr. Suppl.: Table S4; mean 0.142 ± 0.0022 SD; normality of the distribution was confirmed by a Shapiro-Wilk-test: W = 0.957, *p* = 0.191). The 97.5% confidence limit used as a threshold against embryos of higher ploidy expected from heteroploid fertilizations was 0.146. However, in the heteroploid reference sample of pentaploids, embryo-to-standard fluorescence ratios were non-normally distributed (Shapiro-Wilk, W = 0.925, *p* = 0.032) and showed a tentatively bimodal distribution with 35.1% of the values forming the lower peak (Electr. Suppl.: Table S4; mean ± SD 0.145 ± 0.0033) and the remaining 64.9% constituting the upper peak (0.172 ± 0.0104). Only ploidies of the lower peak overlapped with the embryo ploidies determined for the homoploid reference. We therefore inferred the threshold against progeny of homoploid origin only from seeds belonging to the lower peak, which itself was normally distributed (Shapiro-Wilk, W = 0.976, *p*-value = 0.955). This limit was calculated as 0.138 (Fig. 3).

Nevertheless, 75.7 % of the embryo-to-standard fluorescence ratios in the heteroploid pentaploid reference were higher than the homoploid threshold of 0.146. Sixty-eight out of 641 embryos (10.6%) measured for the mixed-ploidy pollinations, including pentaploids, were inferred to have experienced a change in ploidy in accordance with an heteroploid origin (i.e., had a ploidy higher than the homoploid reference), 130 (20.28%) were derived from homoploid fertilization (ploidies lower than the heteroploid reference), and 443 embryos (69.11%) fell within the range of overlap.

However, under the reasonable assumption that ploidy distribution of successful male gametes formed by the pentaploids was similar in both heteroploid and mixed-ploidy pollinations, the 68 before-mentioned seeds should represent 75.7 % of the total number of seeds of heteroploid origin, which we accordingly calculated to be 90 (N = 90), or 14.4% of the total seeds screened from the mixed-ploidy pollinations involving penta-ploids. We used this percentage as an approximation for the fertilization success of apomicts in the mixed-ploidy treatment.

For the heteroploid heptaploid reference, the 2.5% confidence limit was 0.173 (0.191 ± 0.009; Shapiro-Wilk, W = 0.938, *p* = 0.083), a value allowing for unambiguous inference of paternity of seeds. Twenty-three out of 230 embryos (10.0%) measured for mixed-ploidy pollinations including heptaploids showed ploidies from within the distribution of values observed for the heteroploid reference sample, i.e., were of heteroploid origin, and 207 embryos had values indicative of an homoploid origin (90.0%) (Fig. 3).

## Discussion

Our data indicate for the first time the existence of homo-ploid pollen precedence in *Potentilla puberula*. In line with Dobeš & al. (2018), we found the seed yield in heteroploid crosses to be lower than in homoploid crosses, suggesting that heteroploid pollen negatively affects the reproductive success of sexual individuals. However, our current novel investigations with mixed-ploidy pollinations revealed that these losses of sexual progeny due to heteroploid fertilizations were partially restored when homo- and heteroploid pollen was simultaneously applied onto the recipient flower. Seed yield derived from mixed-ploidy fertilizations was intermediate to those obtained in the homo- and heteroploid crosses, but did not significantly differ from the average of these two treatments, suggesting a similar fertilization success of homo- and heteroploid pollen.

However, this equal performance contrasted with the high proportion of homoploid fertilizations (an estimated 85%–87 %) derived from seeds obtained in the mixed-ploidy crosses. These proportions are much higher than expected under a scenario of equal fertilization success of pollen from sexuals and apomicts after correcting for the differential seed yield observed in the homo- versus heteroploid crosses. Thus, under the hypothetical scenario that homo- and heteroploid pollen tubes fertilized egg cells in equal numbers, and the assumption that observed difference in seed yield among these single-pollen treatments is due to unsuccessful fertilizations (see discussion below), homo- and heteroploid progeny would be expected to be 60% (1410 seeds obtained) and 40% (930 seeds). The seed yield was actually lower than these hypothetical values, which might be explained by mutual developmental inhibition of the two applied pollen types. Negative pollen-pollen interactions can arise at different developmental stages of the male gametophyte after pollination. For instance, pollen germination on *Clarkia unguiculata* Lindl stigmas was higher with both pure self and outcrossed pollen than in mixed-pollen pollinations (Németh & Smith-Huerta, 2002). Likewise, interference among growing pollen tubes could occur if a tube grows into a stylar region already occupied by a previous tube, or if multiple tubes attempt to reach the same space/resources at once (Harder & al., 2016), indicating existence of a quantitative effect by number of pollen limiting tube growth.

Similar to our observation of homoploid pollen precedence, Ramsey & al. (2003), observed that interspecific pollinations between two sister species of monkeyflowers (*Mimulus lewisii* Pursh, *M. cardinalis* Douglas ex Benth.) produced nearly 50% fewer seeds than intraspecific crosses, but in mixed-pollen pollinations of *M. cardinalis*, less than 25% of the progeny were hybrids. Koutecký & al. (2011) obtained the same pattern in diploid *Centaurea pseudophrygia* C.A.Mey and tetraploid *C. jacea* L., with lower success in hetero- compared to homoploid treatments, and mixed-ploidy pollination success closer to homoploid values, suggesting that pollen precedence may enhance reproductive isolation among ploidies. An example for a sexual-apomictic system was provided by Mártonfiová (2006, 2015), who studied fertilization of sexual diploids individuals by pollen of triploid apomicts in *Taraxacum* Wigg sect. *Taraxacum* (Syn: *T.* sect. *Ruderalia* Kirschner & al.), and observed that, when sexual diploid individuals received different mixtures of pollen of diploids and apomictic triploids, only diploid progeny was produced, suggesting homoploid pollen precedence.

Various non-exclusive mechanisms can explain pollen precedence. The gametophytically controlled SI system operative in the Rosaceae allows the female reproductive organ to discriminate self-pollen and, consequently, to reduce inbreeding (Brewbaker, 1957; Kao & McCubbin, 1996; Franklin-Tong & Franklin, 2003; Hiscock & McInnis, 2003). Interspecific reproductive barriers recognizing and rejecting interspecific pollen can be mechanistically linked to the SI system, suggesting a possible overlap and a partially shared genetic basis between both mechanisms of post-mating prezygotic female choice (Baek & al., 2015; Tovar-Méndez & al., 2016; Hamlin & al., 2017). In addition to selectively refusing pollen, ovules can be selective in attracting pollen (Takeuchi & Higashiyama, 2012). Such attraction was found to be related to SI: ovules of SI *Solanum* L. species attracted more conspecific pollen compared to SC species (see Lafon-Placette & al., 2016). In *P. puberula* the SI system is functional in the sexual individuals (likely due to diploidization of its assumed allopolyploid genome: Dobeš & al., 2013) and they may well recognize heteroploid pollen, favouring homoploid fertilization.

The presence of heteroploid apomictic pollen reduced the reproductive success of sexual *Potentilla puberula* by means of two mechanisms: Firstly, the significantly lower seed set in the mixed-ploidy compared to the homoploid crosses suggests loss of progeny due to incompatible heteroploid fertilization. Thus, post-zygotic barriers prevent the development of egg cells fertilized by heteroploid pollen into a mature seed. Secondly, a considerable proportion of the progeny of sexuals (more than one-tenth of the developed embryos were derived from fertilizations by heteroploid pollen) was cytologically transformed despite the high degree of homoploid pollen precedence. In sexually derived seeds, such losses by transformation are considered to cause more serious effects than those resulting from endosperm imbalance in apomictically derived seeds, since apomicts are able to avoid cross-fertilization through selfing.

The impact of foreign pollen on the reproductive success may also depend on the phylogeographic history and the biogeographic context, because plants may evolve local adaptive mechanisms. Arceo-Gómez & al. (2016) found that conspecific pollen performed better in terms of pollen tube growth in populations with historical contact of two *Clarkia* species compared to those without. In our case, sexual individuals of *P. puberula* from lowland regions in the south of the study area (region Raas/Fortezza and region Ossenigo/Scaiola), where mainly pure tetraploid populations occur, suffered from higher losses of seeds upon cross-fertilization. In these lower-elevation regions, seed yield from the mixed-ploidy crosses thus was significantly lower compared to the pooled crosses with homo- and heteroploid pollen, a difference that was not significant for the regions from higher elevations. This pattern may indicate that they are more sensitive to interactions with heteroploid pollen than sexual individuals at higher elevations in the north, where apomicts are more frequent.

Mentor effects, defined as the induction of self-fertilization of otherwise self-incompatible sexual plants upon co-application of self- and foreign pollen, are a protective mechanism to avoid cross-fertilization by both, other species and intra-specific cytotypes, including apomicts (Mráz, 2003; Hörandl & Temsch, 2009). In our study, the SI sexual pollen recipients were emasculated and, therefore, unintended selfing – which we would not have been able to discriminate from homoploid cross-fertilizations without genotyping the progeny – was prevented. Consequently, we are not able to quantify the relative role of pollen precedence vs. mentor effects based on our study design. However, mentor effects have already been totally disproven for the mixed-ploidy pollinations including heptaploids and were at least partly excluded for the pentaploids (Dobeš & al., 2018). Hence, yet there is strong evidence that pollen precedence acts as an alternative protection against cross-fertilization, and plays the dominant role as prezygotic barrier in *P. puberula*.

We analysed seed yield as a measure of reproductive success instead of the more commonly used seed set (i.e., ratio mature seeds to ovules). However, we could remove the inter-individual variation in the number of ovules since all treatments were performed in flowers of each pollen recipient. Still, it has to be assumed that there are no differences in the number of ovules among flowers of the same individual allocated to the different treatments. The absence of such a bias seems likely attributable to a random allocation of flowers for a sufficient number of individuals, underlined for *P. puberula* by Dobeš & al. (2018), suggesting non-structured differences in base line seed set among individuals. However, even in case of a violation of the assumption of equal ovule numbers, it is parsimonious to presume that the rate of seed abortion, and hence the discrepancy between seed yield and seed set, would be higher in heteroploid than in homoploid crosses due to reproductive incompatibilities among ploidy levels. Actually, this would rather accentuate than weaken the higher reproductive success found in homoploid crosses, identifying our results as conservative concerning the uncertainties in the determination of reproductive success. Consequently, estimation of seed set would not necessarily increase the reliability of our results since it also rests on an assumption: namely, that the number of ovules and reproductive success does not differ between flowers used to count both ovules and mature seeds.

In conclusion, we found evidence for precedence of homo-over heteroploid pollen in *P. puberula.* However, a considerable proportion of the progeny of sexual plants was lost or transformed upon cross-pollination by their apomictic conspecifics. This likely affects their co-occurrence, because only a complete reproductive isolation avoids – in the long run – the competitive displacement of sexuals by apomicts, unless the former is favoured by other factors related to their reproductive system. In natural populations, the actual success of sexuals versus apomicts depends on a variety of decisive factors like the activity of additional reproductive barriers such as spatial isolation (Dobeš & al., 2013), shifts in flowering time, or assortative pollination, as well as the relative fertility and vigor of sexuals and apomicts. These factors need to be studied in concert with pollen precedence to allow for a quantitative assessment of the degree of pollen precedence necessary to allow a stable co-existence or even a competitive replacement of apomicts. Nevertheless, the observed precedence of homoploid pollen reduces the pressure upon the sexuals in mixed-ploidy populations.

## Supplementary Material

**Supplementary Material** The Electronic Supplement (Fig. S1; Tables S1–S4) is available from https://doi.org/10.12705/676.9.S

Electronic Supplement

## Figures and Tables

**Table 1:** Reproductive success of sexual tetraploid *Potentilla puberula* Krašan individuals from five regions pollinated by homoploid and heteroploid pollen as well as a pollen mixture containing both, and open controls.

Region	Treatment	Flowers pollinated	Flowers failed	Seed yield	Mean ± SD	Median
Zabernig	Homoploid	14	5	37	2.64 ± 3.10	1.5
	Control	12	6	86	4.77 ± 4.62	5.0
	Heteroploid	14	6	16	1.14 ± 1.35	1.0
	Mixed-ploidy	17	13	23	1.35 ± 3.02	0.0

Obersteiner	Homoploid	37	3	401	10.83 ± 8.18	9.0
	Control	21	5	212	8.15 ± 7.52	7.0
	Heteroploid	24	4	188	7.83 ± 8.09	10.0
	Mixed-ploidy	46	2	411	8.90 ± 6.20	8.5

Ossenigo/Scaiola	Homoploid	29	10	159	5.48 ± 6.84	5.0
	Control	14	11	80	3.20 ± 4.69	1.0
	Heteroploid	58	18	225	3.80 ± 5.42	2.0
	Mixed-ploidy	68	30	299	4.40 ± 7.04	1.5

Raas/Fortezza	Homoploid	31	0	343	11.06 ± 5.65	10.0
	Control	13	9	128	5.82 ± 10.10	1.0
	Heteroploid	61	12	289	4.74 ± 4.77	4.0
	Mixed-ploidy	102	15	715	7.01 ± 6.40	5.0

Bodenalm	Homoploid	35	1	470	13.43 ± 10.76	8.5
	Control	17	8	172	6.88 ± 10.78	2.0
	Heteroploid	36	10	212	5.88 ± 10.30	5.0
	Mixed-ploidy	66	8	676	10.24 ± 8.33	9.0

“Flowers failed” represent the number of flowers not producing a single seed. “Mean” and “Median” refer to the number of seed per flower.

**Table 2:** Fixed effect estimates of zero-inflated Poisson Generalized Linear Mixed Models relating the seed yield of sexual tetraploid *Potentilla puberula* Krašan individuals to three pollination treatments applied in an ex situ crossing experiment.

Region	Sample size	Pollen donors	Comparison of treatments	Estimate ± SE	*z* value	*p* value
Zabernig	45/7	8/11	homo ↔ hetero	–0.38 ± 0.16	–2.340	**0.019**
			mixed ↔ (homo, hetero)	–0.26 ± 0.10	2.458	**0.014**

			mixed ↔ homo	0.89 ± 0.52	1.720	0.085
			mixed ↔ hetero	0.07 ± 0.54	0.140	0.886

Obersteiner	107/12	16/13	homo ↔ hetero	–0.12 ± 0.13	–0.884	0.376
			mixed ↔ (homo, hetero)	–0.02 ± 0.06	–0.401	0.689

			mixed ↔ homo	0.20 ± 0.22	0.906	0.365
			mixed ↔ hetero	–0.04 ± 0.23	–0.180	0.857

Ossenigo/Scaiola	155/12	14/43	homo ↔ hetero	–0.41 ± 0.09	–4.350	**<0.001**
			mixed ↔ (homo, hetero)	–0.02 ± 0.06	–0.273	0.784

			mixed ↔ homo	0.45 ± 0.21	2.137	**0.032**
			mixed ↔ hetero	–0.36 ± 0.18	–1.951	0.051

Raas/Fortezza^a^	194/12	16/39	homo ↔ hetero	–0.39 ± 0.06	–6.417	**<0.001**
			mixed ↔ (homo, hetero)	–0.05 ± 0.03	–1.380	0.168

			mixed ↔ homo	0.55 ± 0.13	4.334	**<0.001**
			mixed ↔ hetero	–0.24 ± 0.13	–1.913	0.055

Bodenalm	137/12	18/12	homo ↔ hetero	–0.26 ± 0.11	–2.444	**0.014**
			mixed ↔ (homo, hetero)	0.05 ± 0.05	1.204	0.228

			mixed ↔ homo	0.09 ± 0.15	0.611	0.541
			mixed ↔ hetero	–0.43 ± 0.19	–2.21	**0.027**

“Sample size” refers to the number of pollinated flowers/number of pollen recipient plants. “Pollen donors” indicate the number of sexual/number of apomictic individuals used for the pollinations. The first treatment given in each comparison was used as baseline. Significant differences (α = 0.05) between the treatments are highlighted by bold *p*-values.

a For the Raas/Fortezza region the zero-inflation term was removed from the model due its non-significance and a numerical problem.

## References

[R1] Abadie P, Roussel G, Dencausse B, Bonnet C, Bertocchi E, Louvet JM, Kremer A, Garnier-Géré P (2012). Strength, diversity and plasticity of postmating reproductive barriers between two hybridizing oak species (*Quercus robur* L. and *Quercus petraea* (Matt) Liebl.). J Evol Biol.

[R2] Arceo-Gómez G, Raguso RA, Geber MA (2016). Can plants evolve tolerance mechanisms to heterospecific pollen effects? An experimental test of the adaptive potential in *Clarkia* species. Oikos.

[R3] Arnold ML, Hamrick JL, Bennet BD (1993). Interspecific pollen competition and reproductive insolation in *Iris*. J Heredity.

[R4] Asker SE, Jerling L (1992). Apomixis in plants.

[R5] Baack EJ (2005). Ecological factors influencing tetraploid establishment in snow buttercups (*Ranunculus adoneus*, Ranunculaceae): Minority cytotype exclusion and barriers to triploid formation. Amer J Bot.

[R6] Baek YS, Covey PA, Petersen JJ, Chetelat RT, McClure B, Bedinger PA (2015). Testing the SI x SC rule: Pollen-pistil interactions in interspecific crosses between members of the tomato clade (*Solanum* section *Lycopersicon*, Solanaceae). Amer J Bot.

[R7] Baker MS, Arrigo N, Baniaga AE, Li Z, Levin DA (2016). On the relative abundance of autopolyploids and allopolyploids. New Phytol.

[R8] Bayer RJ (1997). *Antennaria rosea* (Asteraceae) – A model group for the study of the evolution of polyploid agamic complexes. Opera Bot.

[R9] Behrend A, Gluschak A, Przybyla A, Hohe A (2015). Interploid crosses in heather (*Calluna vulgaris*). Sci Hortic.

[R10] Botes C, Johnson SD, Cowling RM (2008). Coexistence of succulent tree aloes: Partitioning of bird pollinators by floral traits and flowering phenology. Oikos.

[R11] Brewbaker JI (1957). Pollen cytology and self-incompatibility systems in plants. J Heredity.

[R12] Brock MT (2009). Prezygotic barriers to gene flow between *Taraxacum ceratophorum* and the invasive *Taraxacum officinale* (Asteraceae). Oecologia.

[R13] Brooks ME, Kristensen K, Van Benthem KJ, Magnusson A, Berg CW, Nielsen A, Skaug HJ, Maechler M (2017). Modeling zero-inflated count data with glmmTMB. bioRxiv.

[R14] Brown BJ, Mitchell RJ (2001). Competition for pollination: Effects of pollen of an invasive plant on seed set of a native congener. Oecologia.

[R15] Brys R, Vanden Broeck A, Mergeay J, Jacquemyn H (2014). The contribution of mating system variation to reproductive isolation in two closely related *Centaurium* species (Gentianaceae) with a generalized flower morphology. Evolution (Lancaster).

[R16] Campbell DR, Alarcón R, Wu CA (2003). Reproductive isolation and hybrid pollen disadvantage in *Ipomopsis*. J Evol Biol.

[R17] Chapman MA, Forbes DG, Abbot RJ (2005). Pollen competition among two species of *Senecio* (Asteraceae) that form a hybrid zone on Mt. Etna, Sicily. Amer J Bot.

[R18] Comai L (2005). The advantages and disadvantages of being polyploid. Amer J Bot.

[R19] Cosendai A-C, Rodewald J, Hörandl E (2011). Origin and distribution of autopolyploids via apomixis in the alpine species *Ranunculus kuepferi* (Ranunculaceae). Taxon.

[R20] Coyne JA, Orr HA (1998). The evolutionary genetics of speciation. Philos Trans, Ser B.

[R21] Dobeš C (1999). Die Karyogeographie des *Potentilla verna* agg. (Rosaceae) in Österreich – mit ergänzenden Angaben aus Slowenien, Kroatien, der Slowakei und Tschechien. Ann Naturhist Mus Wien, B.

[R22] Dobeš C, Milosevic A, Prohaska D, Scheffknecht S, Sharbel TF, Hülber K (2013). Reproductive differentiation into sexual and apomictic polyploid cytotypes in *Potentilla puberula* (Potentilleae, Rosaceae). Ann Bot (Oxford).

[R23] Dobeš C, Scheffknecht S, Fenko Y, Prohaska D, Sykora C, Hülber K (2018). Asymmetric reproductive interference: The consequences of pollination on reproductive success in sexual – Apomictic populations of *Potentilla puberula* (Rosaceae). Ecol Evol.

[R24] Dobzhansky T (1936). Position effects on genes. Biol Rev Cambridge Philos Soc.

[R25] Doležel J, Greilhuber J, Suda J (2007). Estimation of nuclear DNA content in plants using flow cytometry. Nat Protoc.

[R26] Franklin-Tong VE, Franklin FCH (2003). The different mechanisms of gametophytic self-incompatibility. Philos Trans, Ser B.

[R27] Grant V (1981). Plant speciation.

[R28] Grant V (1994). Modes and origins of mechanical and ethological isolation in angiosperms. Proc Natl Acad Sci USA.

[R29] Greilhuber J, Ebert I (1994). Genome size variation in *Pisum sativum*. Genome.

[R30] Hamlin JAP, Sherman NA, Moyle LC (2017). Two loci contribute epistastically to heterospecific pollen rejection, a postmating isolating barrier between species. G3: Genes Genomes Genet.

[R31] Harder LD, Aizen MA, Richards SA, Joseph MA, Busch JW (2016). Diverse ecological relations of male gametophyte populations in stylar environments. Amer J Bot.

[R32] Hersh E, Grimm J, Whitton J (2016). Attack of the clones: Reproductive interference between sexuals and asexuals in the *Crepis* agamic complex. Ecol Evol.

[R33] Hiscock S, McInnis S (2003). The diversity of self-incompatibility system in flowering plants. Pl Biol (Stuttgart).

[R34] Hojsgaard D, Schegg E, Valls JFM, Martínez EJ, Quarin CL (2008). Sexuality, apomixis, ploidy levels, and genomic relationships among four *Paspalum* species of the subgenus *Anachyris* (Poaceae). Flora.

[R35] Hörandl E, Hojsgaard D (2012). The evolution of apomixis in angiosperms: A reappraisal. Pl Biosyst.

[R36] Hörandl E, Temsch EM (2009). Introgression of apomixis into sexual species is inhibited by mentor effects and ploidy barriers in the *Ranunculus auricomus* complex. Ann Bot (Oxford).

[R37] Howard DJ (1999). Conspecific sperm and pollen precedence and speciation. Annual Rev Ecol Evol Syst.

[R38] Hülber K, Sonnleitner M, Flatscher R, Berger A, Dobrovsky R, Niessner S, Nigl T, Schneeweiss GM, Kubešová M, Rauchová J, Suda J (2009). Ecological segregation drives fine-scale cytotype distribution of *Senecio carniolicus* in the Eastern Alps. Preslia.

[R39] Hülber K, Scheffknecht S, Dobeš C, Kroh A, Berning B, Haring E, Harzhauser M, Sattmann H, Walochnik J, Zimmermann D, Zuschin M (2013). Partitioning the factors explaining the eco-geography in the amphi-apomictic species *Potentilla puberula* (Rosaceae).

[R40] Husband BC, Schemske DW (2000). Ecological mechanisms of reproductive isolation between diploid and tetraploid *Chamerion angustifolium*. J Ecol.

[R41] Husband BC, Schemske DW, Burton TL, Goodwillie C (2002). Pollen competition as a unilateral reproductive barrier between sympatric diploid and tetraploid *Chamerion angustifolium*. Proc Roy Soc London, Ser B Biol Sci.

[R42] Joshi A, Moody ME (1995). Male gamete output of asexuals and the dynamics of populations polymorphic for reproductive mode. J Theor Biol.

[R43] Joshi A, Moody ME (1998). The cost of sex revisited: Effects of male gamete output of hermaphrodites that are asexual in their female capacity. J Theor Biol.

[R44] Kao TH, McCubbin AG (1996). How flowering plants discriminate between self and non-self pollen to prevent inbreeding. Proc Natl Acad Sci USA.

[R45] Kay KM (2006). Reproductive isolation between two closely related hummingbird-pollinated neotropical gingers. Evolution (Lancaster).

[R46] Kolár F, Čertner M, Suda J, Schönswetter P, Husband BC (2017). Mixed-ploidy species: Progress and opportunities in polyploid research. Trends Pl Sci.

[R47] Koltunow AM, Grossniklaus U (2003). Apomixis: A developmental perspective. Annual Rev Pl Biol.

[R48] Koutecký P, Badurová T, Štech M, Košnar J, Karásek J (2017). Hybridization between diploid *Centaurea pseudophrygia* and tetraploid *C. jacea* (Asteraceae): The role of mixed pollination, unreduced gametes, and mentor effects. Biol J Linn Soc.

[R49] Kurtto A, Lampinen R, Junikka L (2004). Atlas Florae Europaeae: Distribution of vascular plants in Europe, vol 13, Rosaceae (Spiraea to Fragaria, excl. Rubus.

[R50] Lafon-Placette C, Vallejo-Marín M, Parisod C, Abbott RJ, Köhler C (2016). Current plant speciation research: Unravelling the processes and mechanisms behind the evolution of reproductive isolation barriers. New Phytol.

[R51] Lamont BB, He T, Enright NJ, Krauss SL, Miller BP (2003). Anthropogenic disturbance promotes hybridization between *Banksia* species by altering their biology. J Evol Biol.

[R52] Landis JB, Soltis DE, Li Z, Marx HE, Barker MS, Tank DC, Soltis PS (2018). Impact of whole-genome duplication events on diversification rates in angiosperms. Amer J Bot.

[R53] Lepais O, Roussel G, Hubert F, Kremer A, Gerber S (2013). Strength and variability of postmating reproductive isolating barriers between four European white oak species. Tree Genet Genomes.

[R54] Levin DA (1975). Minority cytotype exclusion in local plant populations. Taxon.

[R55] Lin BY (1984). Ploidy barrier to endosperm development in maize. Genetics.

[R56] Lowry DB, Modliszewski JL, Wright KM, Wu CA, Willis JH (2008). The strength and genetic basis of reproductive isolating barriers in flowering plants. Philos Trans, Ser B.

[R57] Magnusson A, Skaug H, Nielsen A, Berg C, Kristensen K, Maechler M, Van Bentham K, Bolker B, Brooks M (2017). glmmTMB: Generalized Linear Mixed Models using Template Model Builder R package, version 0.1.1. https://cran.r-project.org/web/packages/glmmTMB/index.html.

[R58] Mártonfiová L (2006). Possible pathways of the gene flow in *Taraxacum* sect. *Ruderalia*. Folia Geobot.

[R59] Mártonfiová L (2015). Hybridization in natural mixed populations of sexual diploid and apomictic triploid dandelions (*Taraxacum* sect. *Taraxacum*): Why are the diploid sexuals not forced out?. Folia Geobot.

[R60] Matzk F, Meister A, Schubert I (2000). An efficient screen for reproductive pathways using mature seeds of monocots and dicots. Plant J.

[R61] Mráz P (2003). Mentor effects in the genus *Hieracium* s.str. (Compositae, Lactuceae). Folia Geobot.

[R62] Muller HJ, Jones DF (1932). Further studies on the nature and causes of gene mutations.

[R63] Nardi FD, Dobeš C, Müller D, Grasegger T, Myllynen T, Alonso-Marcos H, Tribsch A (2018). Sexual intraspecific recombination but not *de novo* origin governs the genesis of new apomictic genotypes in *Potentilla puberula* (Rosaceae). Taxon.

[R64] Németh MB, Smith-Huerta NL (2002). Effects on pollen load composition and deposition pattern on pollen performance in *Clarkia unguiculata* (Onagraceae). Int J Pl Sci.

[R65] Nogler GA, Johri BM (1984). Gametophytic apomixis. Embriology of angiosperms.

[R66] Otto FJ (1990). DAPI staining of fixed cells for high-resolution flow cytometry of nuclear DNA. Meth Cell Biol.

[R67] Otto SP, Whitton J (2000). Polyploid incidence and evolution. Annual Rev Genet.

[R68] Ozias-Akins P, Van Dijk PJ (2007). Mendelian genetics of apomixis in plants. Annual Rev Genet.

[R69] Peterson R, Slovin JP, Chen C (2010). A simplified method for differential staining of aborted and non-aborted pollen grains. Int J Pl Biol.

[R70] Petit C, Lesbros P, Ge X, Thompson JD (1997). Variation in flowering phenology and selfing rate across a contact zone between diploid and tetraploid *Arrhenatherum elatius* (Poaceae). Heredity.

[R71] Quantum Analysis (2014). FloMax: Software for multiparameter data analysis, instrument control, and data acquisition for flow cytometry for Windows.

[R72] R Development Core Team (2017). A language and environment for statistical computing.

[R73] Ramsey J, Bradshaw HD, Schemske DW (2003). Components of reproductive isolations between the monkeyflowers *Mimulus lewisii* and *M. cardinalis* (Phrymaceae). Evolution (Lancaster).

[R74] Rieseberg LH, Desrochers AM, Youn SJ (1995). Interspecific pollen competition as a reproductive barrier between sympatric species of *Helianthus* (Asteraceae). Amer J Bot.

[R75] Soják J (2010). Origin of *Potentilla crantzii, P. verna* and *P. puberula* (Rosaceae) with a note on the nomenclature of *P. pusilla*. Feddes Repert.

[R76] Soltis PS, Soltis DE (2016). Ancient WGD events as drivers of key innovations in angiosperms. Curr Opin Pl Biol.

[R77] Soltis DE, Soltis PS, Schemske DW, Hancock JF, Thompson JN, Husband BC, Judd WS (2007). Autopolyploidy in angiosperms: Have we grossly underestimated the number of species?. Taxon.

[R78] Sonnleitner M, Flatscher R, Garcia PE, Rauchova J, Suda J, Schneeweiss GM, Hülber K, Schönswetter P (2010). Distribution and habitat segregation on different spatial scales among diploid, tetraploid and hexaploid cytotypes of *Senecio carniolicus* (Asteraceae) in the Eastern Alps. Ann Bot (Oxford).

[R79] Stebbins GL (1947). Types of polyploids: Their classification and significance. Advances Genet.

[R80] Sutherland BL, Galloway LF (2017). Postzygotic isolation varies by ploidy level within a polyploid complex. New Phytol.

[R81] Takeuchi H, Higashiyama T (2012). A species-specific cluster of defensin-like genes encodes diffusible pollen tube attractants in *Arabidopsis*. PLoS Biol.

[R82] Tribsch A, Schönswetter P (2003). Patterns of endemism and comparative phylogeography confirm paleoenvironmental evidence for Pleistocene refugia in the Eastern Alps. Taxon.

[R83] Trunschke J, Stöcklin J (2017). Plasticity of flower longevity in alpine plants is increased in populations from high elevation compared to low elevation populations. Alpine Bot.

[R84] Tovar-Méndez A, Lu L, Mcclure B (2016). HT proteins contribute to S-RNAse-independent pollen rejection in *Solanum*. Plant J.

[R85] Wyatt R, Boyles SB, Derda GS (1992). Environmental influences on nectar production in milkweeds (*Asclepias syriaca* and *A. exaltata*). Amer J Bot.

