# Supplementary material for: Pollen precedence in sexual Potentilla puberula and its role as a protective reproductive barrier against apomictic cytotypes
Source: Taxon. Author manuscript; Available in PMC 2019 Feb 9. (PMC6368848; doi:10.12705/676.9)
Supplement: Electronic Supplement [file NIHMS81461-supplement-Electronic_Supplement.pdf]

Vol. 67 (6) • December 2018

TAXON

International Journal of Taxonomy, Phylogeny and Evolution

Electronic Supplement to

Pollen precedence in sexual *Potentilla puberula* and its role as a protective reproductive barrier against apomictic cytotypes

Henar Alonso-Marcos, Karl Hülber, Tuuli Myllynen, Patricia Pérez Rodríguez & Christoph Dobeš

***Taxon* 67: 1132–1142 (https://doi.org/10.12705/676.9)**

https://doi.org/10.12705/676.9.S

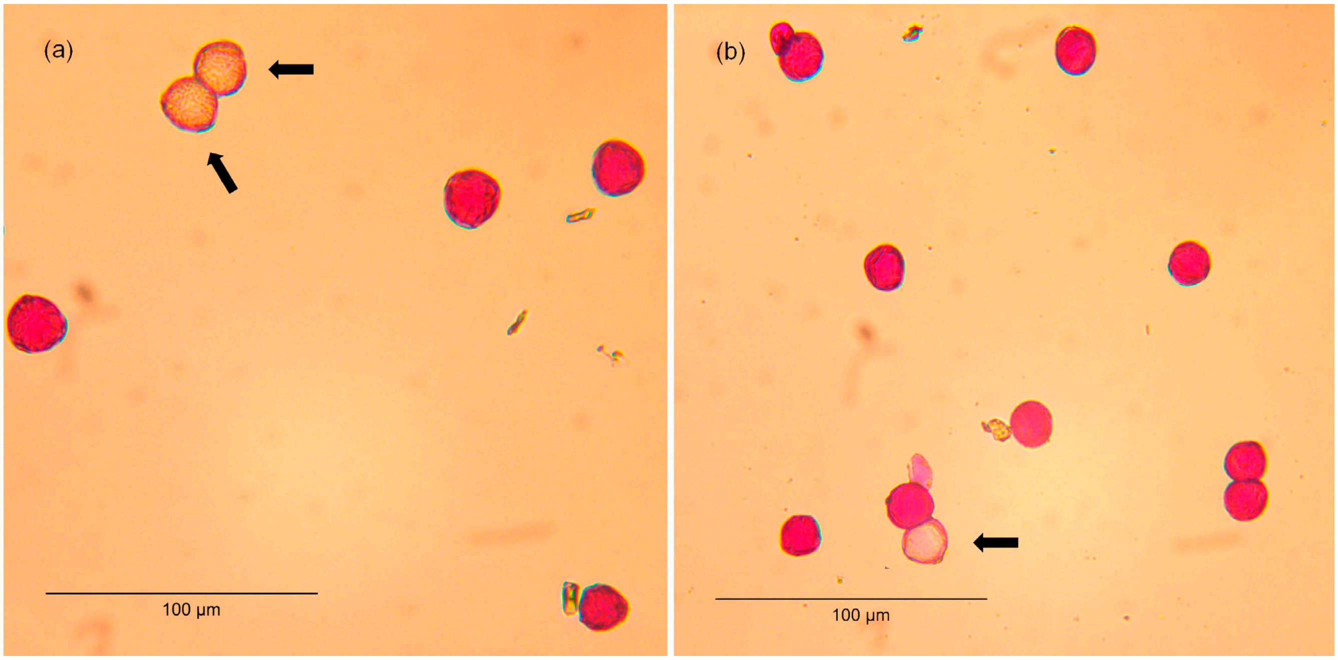

Fig. S1. Peterson's vitality stain used to differentiate vital (i.e., stained, red grains) and aborted pollen (non-stained grains, indicated by arrows) of *Potentilla puberula* Krašan exemplified with **(A)** a heptaploid individual from region Raas/Fortezza (119-72) and **(B)** a pentaploid individual from Bodenalm (144-25), Laborlux S microscope, 400×.

Table S1. Descriptive statistics of a controlled ex situ crossing experiment carried out on sexual and apomictic individuals of *Potentilla puberula* Krašan from five local regions from East Tyrol, Austria, and South Tyrol and the Trentino/Lombardia, Italy. Fifty-five sexual tetraploid individuals served as pollen recipient which were pollinated by A) 72 sexual tetraploid ("Treatment" IA), 74 apomictic pentaploid (IE 5*x*) and additionally in two regions 44 apomictic heptaploid pollen donors (IE 7x), and B) mixtures of equivalent amounts of pollen from sexual and apomictic donors (MIX). In the regions Ossenigo/Scaiola and Raas/Fortezza, which comprise two populations, the first mentioned population furnished the sexual cytotypes, the second mentioned the apomictic pollen donors. The initial letter in front of each donor indicates from which of the two populations is the individual. For each manipulation, the pollen "Recipient", the "Donor" and the obtained "Seed yield" is provided.

A) Homo- and heteroploid crosses

Region	Recipient	Donor	Treatment	Seed yield
Zabernig	119	57	IE 5*x*	2
	119	61	IE 5*x*	0
	119	130	IA	0
	119	136	IA	1
	125	69	IE 5*x*	1
	125	105	IE 5*x*	0
	125	130	IA	6
	125	136	IA	0
	125	136	IA	6
	137	56	IE 5*x*	0
	137	71	IE 5*x*	1
	137	88	IE 5*x*	1
	137	119	IA	2
	137	130	IA	0
	137	136	IA	4
	140	56	IE 5*x*	3
	140	106	IE 5*x*	1
	140	111	IA	0
	142	57	IE 5*x*	0
	142	57	IE 5*x*	4
	142	110	IE 5*x*	0
	142	119	IA	5
	142	147	IA	2
	142	148	IA	1
	147	94	IE 5*x*	0
	147	120	IA	0
	148	60	IE 5*x*	3
	148	129	IA	10
Obersteiner	56	38	IE 5*x*	15
	56	43	IA	18
	56	48	IA	1
	56	57	IE 5*x*	3
	103	36	IE 5*x*	10
	103	43	IA	13
	103	44	IA	17
	103	46	IA	3
	103	145	IE 5*x*	11
	103	148	IE 5*x*	4
	104	44	IA	7
Obersteiner *(continued)*	104	54	IE 5*x*	0
	104	58	IE 5*x*	0
	104	127	IA	1
	104	140	IA	19
	104	145	IE 5*x*	13
	105	38	IE 5*x*	3
	105	41	IE 5*x*	12
	105	47	IA	17
	105	106	IA	0
	105	126	IA	23
	106	35	IA	5
	106	46	IA	10
	106	48	IA	1
	106	121	IE 5*x*	12
	109	41	IE 5*x*	13
	109	48	IA	5
	109	103	IA	13
	109	138	IE 5*x*	19
	109	139	IA	12
	110	36	IE 5*x*	1
	110	47	IA	6
	110	58	IE 5*x*	10
	110	106	IA	11
	113	48	IA	1
	113	96	IA	0
	113	106	IA	1
	113	125	IE 5*x*	0
	113	133	IE 5*x*	0
	116	35	IA	5
	116	45	IA	21
	116	58	IE 5*x*	8
	116	103	IA	9
	116	139	IA	13
	116	148	IE 5*x*	4
	126	45	IA	7
	126	140	IA	20
	126	140	IA	16
	143	43	IA	39
	143	45	IA	26

Table S1. Continued.

A) Homo- and heteroploid crosses

Region	Recipient	Donor	Treatment	Seed yield
Obersteiner *(continued)*	143	48	IA	0
	143	48	IA	9
	143	121	IE 5*x*	16
	143	133	IE 5*x*	2
	146	31	IA	3
	146	36	IE 5*x*	14
	146	41	IE 5*x*	9
	146	104	IA	11
	146	111	IE 5*x*	9
	146	127	IA	14
	146	127	IA	24
Ossenigo/ Scaiola	14	O 20	IA	32
	14	S 5	IE 7*x*	8
	14	S 5	IE 7*x*	7
	14	S 13	IE 7*x*	22
	14	S 30	IE 5*x*	9
	14	S 30	IE 5*x*	9
	14	S 37	IE 5*x*	17
	14	S 37	IE 5*x*	23
	14	S 71	IE 7*x*	16
	14	S 117	IE 5*x*	16
	15	O 4	IA	0
	15	O 14	IA	0
	15	O 20	IA	0
	15	O 55	IA	1
	15	S 10	IE 7*x*	0
	15	S 22	IE 5*x*	0
	15	S 37	IE 5*x*	0
	16	O 13	IA	0
	16	O 34	IA	0
	16	O 49	IA	0
	16	S 22	IE 5*x*	1
	16	S 36	IE 5*x*	3
	16	S 91	IE 5*x*	1
	17	O 12	IA	6
	17	O 49	IA	5
	17	O 55	IA	11
	17	S 6	IE 7*x*	2
	17	S 7	IE 5*x*	0
	17	S 29	IE 5*x*	1
	17	S 34	IE 5*x*	2
	17	S 34	IE 5*x*	6
	17	S 42	IE 7*x*	3
	18	O 12	IA	12

Region	Recipient	Donor	Treatment	Seed yield
Ossenigo/ Scaiola *(continued)*	18	O 14	IA	9
	18	O 17	IA	10
	18	S 35	IE 5*x*	4
	18	S 41	IE 5*x*	3
	18	S 48	IE 7*x*	1
	18	S 80	IE 7*x*	7
	18	S 84	IE 7*x*	5
	20	O 18	IA	1
	20	O 21	IA	5
	20	O 55	IA	4
	20	S 16	IE 7*x*	7
	20	S 16	IE 7*x*	0
	20	S 27	IE 5*x*	3
	20	S 32	IE 5*x*	4
	20	S 45	IE 7*x*	1
	20	S 45	IE 7*x*	0
	20	S 57	IE 7*x*	1
	20	S 90	IE 5*x*	1
	27	O 16	IA	0
	27	O 20	IA	9
	27	O 32	IA	15
	27	S 16	IE 7*x*	8
	27	S 21	IE 5*x*	2
	27	S 31	IE 5*x*	2
	27	S 33	IE 5*x*	0
	27	S 100	IE 7*x*	3
	27	S 100	IE 7*x*	8
	29	O 4	IA	8
	29	O 32	IA	5
	29	S 8	IE 7*x*	2
	36	O 12	IA	0
	36	S 35	IE 5*x*	0
	36	S 38	IE 5*x*	7
	36	S 54	IE 7*x*	0
	36	S 63	IE 7*x*	1
	42	O 18	IA	0
	42	S 57	IE 7*x*	0
	42	S 90	IE 5*x*	0
	44	O 13	IA	5
	44	O 22	IA	0
	44	O 34	IA	3
	44	S 27	IE 5*x*	0
	44	S 30	IE 5*x*	0
	44	S 55	IE 7*x*	2

Table S1. Continued.

A) Homo- and heteroploid crosses

Region	Recipient	Donor	Treatment	Seed yield
	44	S 70	IE 7*x*	2
	44	S 95	IE 5*x*	0
	48	O 13	IA	6
	48	O 55	IA	12
	48	S 29	IE 5*x*	3
	48	S 46	IE 7*x*	0
	48	S 65	IE 5*x*	0
	48	S 74	IE 7*x*	0
	48	S 78	IE 7*x*	2
	48	S 86	IE 5*x*	0
Raas/ Fortezza	16	F 48	IE 5*x*	0
	16	F 59	IE 7*x*	0
	16	F 60	IE 5*x*	1
	18	F 12	IE 5*x*	6
	18	F 13	IE 5*x*	2
	18	F 19	IE 5*x*	0
	18	F 43	IE 7*x*	0
	18	F 59	IE 7*x*	7
	18	R 29	IA	7
	18	R 31	IA	15
	18	R 33	IA	3
	20	F 3	IE 5*x*	6
	20	F 18	IE 7*x*	24
	20	F 18	IE 7*x*	18
	20	F 24	IE 5*x*	16
	20	F 60	IE 5*x*	4
	20	R 3	IA	15
	20	R 8	IA	18
	20	R 8	IA	6
	20	R 10	IA	21
	22	F 14	IE 7*x*	2
	22	F 25	IE 5*x*	4
	22	F 27	IE 7*x*	0
	22	F 28	IE 5*x*	1
	22	F 33	IE 5*x*	2
	22	R 3	IA	9
	22	R 6	IA	9
	22	R 52	IA	11
	26	F 9	IE 7*x*	2
	26	F 11	IE 7*x*	2
	26	F 28	IE 5*x*	0
	26	F 41	IE 7*x*	3
	26	F 44	IE 5*x*	7
	26	F 47	IE 5*x*	4

Region	Recipient	Donor	Treatment	Seed yield
	26	R 2	IA	10
	26	R 10	IA	12
	26	R 11	IA	9
	27	F 8	IE 5*x*	9
	27	F 16	IE 7*x*	3
	27	F 48	IE 5*x*	5
	27	F 49	IE 7*x*	5
	27	F 60	IE 5*x*	3
	27	R 2	IA	5
	27	R 6	IA	22
	27	R 9	IA	24
	30	F 19	IE 5*x*	1
	30	F 31	IE 5*x*	7
	30	F 32	IE 5*x*	5
	30	F 67	IE 7*x*	10
	30	F 73	IE 7*x*	0
	30	F 73	IE 7*x*	5
	30	R 12	IA	20
	30	R 13	IA	11
	30	R 52	IA	13
	36	F 1	IE 7*x*	4
	36	F 12	IE 5*x*	10
	36	F 23	IE 7*x*	12
	36	F 26	IE 7*x*	6
	36	F 29	IE 7*x*	8
	36	F 33	IE 5*x*	5
	36	F 47	IE 5*x*	3
	36	R 5	IA	8
	36	R 12	IA	11
	36	R 29	IA	2
	37	F 8	IE 5*x*	3
	37	F 13	IE 5*x*	4
	37	F 23	IE 7*x*	4
	37	F 51	IE 7*x*	2
	37	F 53	IE 7*x*	4
	37	R 5	IA	11
	37	R 11	IA	9
	37	R 13	IA	7
	38	F 8	IE 5*x*	0
	38	F 12	IE 5*x*	0
	40	F 25	IE 5*x*	13
	40	F 32	IE 5*x*	8
	40	F 50	IE 7*x*	6
	40	F 63	IE 7*x*	1

Table S1. Continued.

A) Homo- and heteroploid crosses

Region	Recipient	Donor	Treatment	Seed yield
Ossenigo/ Scaiola *(continued)*	40	F 65	IE 7*x*	5
	40	F 66	IE 5*x*	5
	40	R 3	IA	9
	40	R 6	IA	18
	40	R 8	IA	6
	43	F 3	IE 5*x*	0
	43	F 4	IE 7*x*	2
	43	F 16	IE 7*x*	0
	43	F 31	IE 5*x*	11
	43	F 37	IE 7*x*	0
	43	F 66	IE 5*x*	9
	43	R 31	IA	8
	43	R 35	IA	4
	43	R 44	IA	10
Bodenalm	29	2	IE 5*x*	0
	29	8	IA	40
	29	28	IA	28
	29	28	IA	27
	31	4	IA	11
	31	5	IE 5*x*	1
	31	6	IA	2
	31	8	IA	4
	31	10	IE 5*x*	20
	31	26	IE 5*x*	1
	33	20	IA	15
	33	21	IA	31
	33	21	IA	9
	33	22	IA	18
	33	25	IE 5*x*	11
	33	27	IE 5*x*	0
	33	96	IE 5*x*	0
	37	2	IE 5*x*	22
	37	10	IE 5*x*	9
	37	30	IA	31
	37	50	IA	10
	37	77	IA	34
	37	99	IE 5*x*	24
	47	9	IA	30
	47	15	IE 5*x*	32
	47	18	IA	4
	47	27	IE 5*x*	17
	47	30	IA	13
	47	69	IE 5*x*	0

Region	Recipient	Donor	Treatment	Seed yield
Bodenalm *(continued)*	49	15	IE 5*x*	2
	49	37	IA	8
	49	47	IA	1
	49	50	IA	12
	49	96	IE 5*x*	1
	49	100	IE 5*x*	3
	49	100	IE 5*x*	0
	50	25	IE 5*x*	8
	50	37	IA	12
	50	49	IA	23
	50	77	IA	6
	50	100	IE 5*x*	5
	50	100	IE 5*x*	4
	51	4	IA	14
	51	6	IA	17
	51	15	IE 5*x*	1
	51	18	IA	5
	51	26	IE 5*x*	1
	51	97	IE 5*x*	9
	51	100	IE 5*x*	2
	56	2	IE 5*x*	0
	56	2	IE 5*x*	0
	56	2	IE 5*x*	6
	56	28	IA	0
	56	29	IA	13
	56	47	IA	10
	56	69	IE 5*x*	0
	56	97	IE 5*x*	4
	56	97	IE 5*x*	4
	57	5	IE 5*x*	2
	57	9	IA	4
	57	26	IE 5*x*	2
	57	27	IE 5*x*	2
	57	36	IA	3
	57	50	IA	5
	58	29	IA	20
	60	9	IA	1
	60	22	IA	8
	60	25	IE 5*x*	19
	60	35	IE 5*x*	0
	60	47	IA	1
	60	97	IE 5*x*	0

Table S1. Continued.

B) Mixed crosses

Population	Recipient	Donor 1	Donor 2	Treatment	Seed yield
Zabernig	119	120	57	MIX 4*x* + 5*x*	0
	137	136	88	MIX 4*x* + 5*x*	0
	137	130	56	MIX 4*x* + 5*x*	0
	137	119	71	MIX 4*x* + 5*x*	0
	137	56	130	MIX 5*x* + 4*x*	0
	137	71	119	MIX 5*x* + 4*x*	0
	140	111	106	MIX 4*x* + 5*x*	4
	140	106	111	MIX 5*x* + 4*x*	0
	142	147	97	MIX 4*x* + 5*x*	0
	142	147	97	MIX 4*x* + 5*x*	2
	142	147	97	MIX 4*x* + 5*x*	11
	142	137	99	MIX 4*x* + 5*x*	0
	142	97	147	MIX 5*x* + 4*x*	0
	142	94	136	MIX 5*x* + 4*x*	6
	147	136	94	MIX 4*x* + 5*x*	0
	147	94	136	MIX 5*x* + 4*x*	0
	148	60	136	MIX 5*x* + 4*x*	0
Obersteiner	103	36	43	MIX 5*x* + 4*x*	5
	103	43	36	MIX 4*x* + 5*x*	7
	103	44	145	MIX 4*x* + 5*x*	12
	103	145	44	MIX 5*x* + 4*x*	17
	104	44	58	MIX 4*x* + 5*x*	13
	104	54	127	MIX 5*x* + 4*x*	8
	104	58	44	MIX 5*x* + 4*x*	3
	104	127	54	MIX 4*x* + 5*x*	14
	104	140	145	MIX 4*x* + 5*x*	15
	104	145	140	MIX 5*x* + 4*x*	9
	105	38	47	MIX 5*x* + 4*x*	7
	105	41	106	MIX 5*x* + 4*x*	13
	105	47	38	MIX 4*x* + 5*x*	2
	105	106	41	MIX 4*x* + 5*x*	18
	106	35	121	MIX 4*x* + 5*x*	3
	106	121	35	MIX 5*x* + 4*x*	5
	109	41	43	MIX 5*x* + 4*x*	4
	109	43	41	MIX 4*x* + 5*x*	11
	109	103	138	MIX 4*x* + 5*x*	14
	109	138	103	MIX 5*x* + 4*x*	16
	110	36	106	MIX 5*x* + 4*x*	12
	110	47	58	MIX 4*x* + 5*x*	4
	110	58	47	MIX 5*x* + 4*x*	6

Population	Recipient	Donor 1	Donor 2	Treatment	Seed yield
Obersteiner *(continued)*	110	106	36	MIX 4*x* + 5*x*	1
	110	127	145	MIX 4*x* + 5*x*	3
	110	145	127	MIX 5*x* + 4*x*	1
	113	46	125	MIX 4*x* + 5*x*	4
	113	106	111	MIX 4*x* + 5*x*	1
	113	125	46	MIX 5*x* + 4*x*	3
	116	35	58	MIX 4*x* + 5*x*	1
	116	45	148	MIX 4*x* + 5*x*	17
	116	58	35	MIX 5*x* + 4*x*	0
	116	148	45	MIX 5*x* + 4*x*	10
	126	36	103	MIX 5*x* + 4*x*	17
	126	103	36	MIX 4*x* + 5*x*	18
	143	43	121	MIX 4*x* + 5*x*	18
	143	45	57	MIX 4*x* + 5*x*	14
	143	45	133	MIX 4*x* + 5*x*	3
	143	121	43	MIX 5*x* + 4*x*	18
	143	133	45	MIX 5*x* + 4*x*	5
	146	31	36	MIX 4*x* + 5*x*	9
	146	36	31	MIX 5*x* + 4*x*	9
	146	41	127	MIX 5*x* + 4*x*	0
	146	104	111	MIX 4*x* + 5*x*	12
	146	111	104	MIX 5*x* + 4*x*	23
	146	127	41	MIX 4*x* + 5*x*	6
Ossenigo/ Scaiola	14	O 18	S 30	MIX 4*x* + 5*x*	12
	14	O 20	S 71	MIX 4*x* + 7*x*	20
	14	O 20	S 117	MIX 4*x* + 5*x*	16
	14	S 30	O 18	MIX 5*x* + 4*x*	24
	14	S 37	O 43	MIX 5*x* + 4*x*	38
	14	O 43	S 37	MIX 4*x* + 5*x*	2
	14	S 71	O 20	MIX 7*x* + 4*x*	14
	14	S 117	O 20	MIX 5*x* + 4*x*	13
	15	S 4	O 22	MIX 5*x* + 4*x*	0
	15	O 12	S 37	MIX 4*x* + 5*x*	0
	15	O 12	S 78	MIX 4*x* + 7*x*	0
	15	O 22	S 4	MIX 4*x* + 5*x*	0
	15	S 37	O 12	MIX 5*x* + 4*x*	0
	15	S 78	O 12	MIX 7*x* + 4*x*	0
	16	O 13	S 88	MIX 4*x* + 7*x*	0
	16	O 13	S 91	MIX 4*x* + 5*x*	0
	16	S 22	O 49	MIX 5*x* + 4*x*	0

Table S1. Continued.

B) Mixed crosses

Population	Recipient	Donor 1	Donor 2	Treatment	Seed yield
Ossenigo/ Scaiola *(continued)*	16	O 34	S 36	MIX 4*x* + 5*x*	0
	16	O 34	S 87	MIX 4*x* + 7*x*	0
	16	S 36	O 34	MIX 5*x* + 4*x*	2
	16	O 49	S 22	MIX 4*x* + 5*x*	0
	16	S 87	O 34	MIX 7*x* + 4*x*	0
	16	S 91	O 13	MIX 5*x* + 4*x*	0
	17	S 6	O 55	MIX 7*x* + 4*x*	6
	17	S 7	O 55	MIX 5*x* + 4*x*	0
	17	O 12	S 29	MIX 4*x* + 5*x*	3
	17	O 12	S 42	MIX 4*x* + 7*x*	0
	17	S 29	O 12	MIX 5*x* + 4*x*	0
	17	S 34	O 49	MIX 5*x* + 4*x*	4
	17	S 42	O 12	MIX 7*x* + 4*x*	0
	17	O 49	S 34	MIX 4*x* + 5*x*	5
	17	O 55	S 6	MIX 4*x* + 7*x*	8
	17	O 55	S 7	MIX 4*x* + 5*x*	2
	18	O 12	S 35	MIX 4*x* + 5*x*	0
	18	S 35	O 12	MIX 5*x* + 4*x*	0
	20	S 16	O 18	MIX 7*x* + 4*x*	3
	20	O 18	S 16	MIX 4*x* + 7*x*	10
	20	O 18	S 95	MIX 4*x* + 5*x*	2
	20	O 21	S 32	MIX 4*x* + 5*x*	1
	20	O 21	S 57	MIX 4*x* + 7*x*	0
	20	S 32	O 21	MIX 5*x* + 4*x*	2
	20	S 57	O 21	MIX 7*x* + 4*x*	0
	20	S 95	O 18	MIX 5*x* + 4*x*	2
	27	O 12	S 33	MIX 4*x* + 5*x*	11
	27	O 12	S 120	MIX 4*x* + 7*x*	16
	27	O 16	S 100	MIX 4*x* + 7*x*	0
	27	O 32	S 16	MIX 4*x* + 7*x*	14
	27	O 32	S 16	MIX 4*x* + 7*x*	16
	27	S 100	O 16	MIX 7*x* + 4*x*	2
	27	S 120	O 12	MIX 7*x* + 4*x*	11
	36	O 12	S 35	MIX 4*x* + 5*x*	3
	36	S 35	O 12	MIX 5*x* + 4*x*	0
	42	O 21	S 57	MIX 4*x* + 7*x*	0
	44	O 13	S 30	MIX 4*x* + 5*x*	0
	44	O 13	S 70	MIX 4*x* + 7*x*	0
	44	O 22	S 27	MIX 4*x* + 5*x*	1
	44	S 27	O 22	MIX 5*x* + 4*x*	2

Population	Recipient	Donor 1	Donor 2	Treatment	Seed yield
Ossenigo/ Scaiola *(continued)*	44	O 34	S 55	MIX 4*x* + 7*x*	10
	44	S 55	O 34	MIX 7*x* + 4*x*	3
	44	S 70	O 13	MIX 7*x* + 4*x*	0
	48	O 12	S 29	MIX 4*x* + 5*x*	0
	48	O 12	S 46	MIX 4*x* + 7*x*	1
	48	O 27	S 74	MIX 4*x* + 7*x*	6
	48	S 29	O12	MIX 5*x* + 4*x*	1
	48	S 29	O 43	MIX 5*x* + 4*x*	5
	48	O 43	S 29	MIX 4*x* + 5*x*	0
	48	S 46	O 12	MIX 7*x* + 4*x*	4
	48	S 74	O 27	MIX 7*x* + 4*x*	4
	16	31	48	MIX 4*x* + 5*x*	2
Raas/ Fortezza	16	48	31	MIX 5*x* + 4*x*	1
	18	12	31	MIX 5*x* + 4*x*	10
	18	13	33	MIX 5*x* + 4*x*	4
	18	19	29	MIX 5*x* + 4*x*	11
	18	29	19	MIX 4*x* + 5*x*	0
	18	31	12	MIX 4*x* + 5*x*	0
	18	33	13	MIX 4*x* + 5*x*	3
	18	52	53	MIX 4*x* + 7*x*	3
	18	53	52	MIX 7*x* + 4*x*	11
	20	3	3	MIX 4*x* + 5*x*	5
	20	3	3	MIX 5*x* + 4*x*	1
	20	3	10	MIX 4*x* + 7*x*	14
	20	10	3	MIX 7*x* + 4*x*	14
	20	10	24	MIX 4*x* + 5*x*	14
	20	24	10	MIX 5*x* + 4*x*	1
	20	31	50	MIX 4*x* + 7*x*	19
	20	31	50	MIX 4*x* + 7*x*	20
	20	31	60	MIX 4*x* + 5*x*	2
	20	50	31	MIX 7*x* + 4*x*	5
	20	60	31	MIX 5*x* + 4*x*	16
	22	3	14	MIX 4*x* + 7*x*	9
	22	6	27	MIX 4*x* + 7*x*	11
	22	6	28	MIX 4*x* + 5*x*	1
	22	10	52	MIX 7*x* + 4*x*	5
	22	14	3	MIX 7*x* + 4*x*	6
	22	25	52	MIX 5*x* + 4*x*	0
	22	27	6	MIX 7*x* + 4*x*	3
	22	28	6	MIX 5*x* + 4*x*	1

Table S1. Continued.

B) Mixed crosses

Population	Recipient	Donor 1	Donor 2	Treatment	Seed yield
Raas/ Fortezza *(continued)*	22	52	10	MIX 4*x* + 7*x*	5
	22	52	25	MIX 4*x* + 5*x*	2
	26	2	9	MIX 4*x* + 7*x*	24
	26	2	47	MIX 4*x* + 5*x*	8
	26	9	2	MIX 7*x* + 4*x*	25
	26	10	11	MIX 4*x* + 7*x*	0
	26	10	28	MIX 4*x* + 5*x*	7
	26	11	10	MIX 7*x* + 4*x*	3
	26	11	41	MIX 4*x* + 7*x*	0
	26	11	44	MIX 4*x* + 5*x*	3
	26	28	10	MIX 5*x* + 4*x*	0
	26	41	11	MIX 7*x* + 4*x*	0
	26	44	11	MIX 5*x* + 4*x*	7
	26	47	2	MIX 5*x* + 4*x*	2
	27	2	46	MIX 4*x* + 7*x*	0
	27	2	48	MIX 4*x* + 5*x*	22
	27	6	8	MIX 4*x* + 5*x*	10
	27	6	49	MIX 4*x* + 7*x*	12
	27	8	6	MIX 5*x* + 4*x*	7
	27	8	6	MIX 5*x* + 4*x*	6
	27	46	2	MIX 7*x* + 4*x*	13
	27	49	6	MIX 7*x* + 4*x*	8
	30	12	19	MIX 4*x* + 5*x*	3
	30	12	22	MIX 4*x* + 7*x*	14
	30	13	31	MIX 4*x* + 5*x*	12
	30	13	67	MIX 4*x* + 7*x*	10
	30	19	12	MIX 5*x* + 4*x*	0
	30	31	52	MIX 5*x* + 4*x*	10
	30	32	13	MIX 5*x* + 4*x*	8
	30	52	31	MIX 4*x* + 5*x*	0
	30	52	73	MIX 4*x* + 7*x*	16
	30	67	13	MIX 7*x* + 4*x*	8
	30	73	52	MIX 7*x* + 4*x*	8
	36	2	64	MIX 4*x* + 7*x*	6
	36	5	26	MIX 4*x* + 7*x*	10
	36	12	12	MIX 4*x* + 5*x*	1
	36	12	12	MIX 5*x* + 4*x*	4
	36	26	5	MIX 7*x* + 4*x*	4
	36	29	29	MIX 4*x* + 7*x*	1
	36	29	29	MIX 7*x* + 4*x*	13
Raas/ Fortezza *(continued)*	36	29	47	MIX 4*x* + 5*x*	7
	36	47	29	MIX 5*x* + 4*x*	1
	36	64	12	MIX 7*x* + 4*x*	10
	37	5	8	MIX 4*x* + 5*x*	1
	37	5	52	MIX 7*x* + 4*x*	0
	37	5	53	MIX 4*x* + 7*x*	5
	37	8	5	MIX 5*x* + 4*x*	4
	37	11	24	MIX 4*x* + 5*x*	6
	37	13	13	MIX 4*x* + 5*x*	7
	37	13	13	MIX 5*x* + 4*x*	10
	37	13	51	MIX 4*x* + 7*x*	3
	37	24	11	MIX 5*x* + 4*x*	8
	37	51	13	MIX 7*x* + 4*x*	4
	37	52	5	MIX 4*x* + 7*x*	4
	37	53	5	MIX 7*x* + 4*x*	3
	40	3	66	MIX 4*x* + 5*x*	7
	40	6	25	MIX 4*x* + 5*x*	2
	40	6	32	MIX 4*x* + 5*x*	17
	40	6	32	MIX 4*x* + 5*x*	26
	40	32	6	MIX 5*x* + 4*x*	16
	40	66	3	MIX 5*x* + 4*x*	18
	43	3	33	MIX 5*x* + 4*x*	14
	43	4	33	MIX 7*x* + 4*x*	0
	43	12	16	MIX 4*x* + 7*x*	0
	43	12	16	MIX 4*x* + 7*x*	3
	43	16	12	MIX 7*x* + 4*x*	1
	43	16	12	MIX 7*x* + 4*x*	3
	43	31	31	MIX 4*x* + 5*x*	13
	43	31	31	MIX 5*x* + 4*x*	0
	43	33	3	MIX 4*x* + 5*x*	17
	43	33	4	MIX 4*x* + 7*x*	0
	43	44	66	MIX 4*x* + 5*x*	16
	43	66	44	MIX 5*x* + 4*x*	5
	29	2	8	MIX 5*x* + 4*x*	0
Bodenalm	29	8	2	MIX 4*x* + 5*x*	31
	29	99	20	MIX 5*x* + 4*x*	0
	31	4	5	MIX 4*x* + 5*x*	9
	31	5	4	MIX 5*x* + 4*x*	9
	31	6	10	MIX 4*x* + 5*x*	1
	31	8	26	MIX 4*x* + 5*x*	3

Table S1. Continued.

B) Mixed crosses

Population	Recipient	Donor 1	Donor 2	Treatment	Seed yield
Bodenalm *(continued)*	31	10	6	MIX 5*x* + 4*x*	18
	31	26	8	MIX 5*x* + 4*x*	1
	33	20	25	MIX 4*x* + 5*x*	0
	33	21	96	MIX 4*x* + 5*x*	0
	33	22	27	MIX 4*x* + 5*x*	0
	33	25	20	MIX 5*x* + 4*x*	6
	33	27	22	MIX 5*x* + 4*x*	15
	33	96	21	MIX 5*x* + 4*x*	11
	37	2	77	MIX 5*x* + 4*x*	13
	37	10	50	MIX 5*x* + 4*x*	22
	37	30	99	MIX 4*x* + 5*x*	13
	37	50	10	MIX 4*x* + 5*x*	30
	37	77	2	MIX 4*x* + 5*x*	2
	37	99	30	MIX 5*x* + 4*x*	10
	47	9	27	MIX 4*x* + 5*x*	14
	47	15	18	MIX 5*x* + 4*x*	28
	47	18	15	MIX 4*x* + 5*x*	24
	47	27	9	MIX 5*x* + 4*x*	23
	47	30	69	MIX 4*x* + 5*x*	17
	47	69	30	MIX 5*x* + 4*x*	0
	49	15	47	MIX 5*x* + 4*x*	13
	49	37	100	MIX 4*x* + 5*x*	2
	49	47	15	MIX 4*x* + 5*x*	3
	49	50	96	MIX 4*x* + 5*x*	4
	49	96	50	MIX 5*x* + 4*x*	5
	49	100	37	MIX 5*x* + 4*x*	3
	50	25	49	MIX 5*x* + 4*x*	13
	50	35	37	MIX 5*x* + 4*x*	9
	50	37	35	MIX 4*x* + 5*x*	5
	50	49	25	MIX 4*x* + 5*x*	25

Population	Recipient	Donor 1	Donor 2	Treatment	Seed yield
Bodenalm *(continued)*	50	77	100	MIX 4*x* + 5*x*	18
	50	100	77	MIX 4*x* + 5*x*	17
	51	4	26	MIX 4*x* + 5*x*	18
	51	6	15	MIX 4*x* + 5*x*	12
	51	18	97	MIX 4*x* + 5*x*	0
	51	26	4	MIX 5*x* + 4*x*	13
	51	97	18	MIX 5*x* + 4*x*	6
	56	2	29	MIX 5*x* + 4*x*	19
	56	28	97	MIX 4*x* + 5*x*	22
	56	29	2	MIX 4*x* + 5*x*	18
	56	47	99	MIX 4*x* + 5*x*	9
	56	97	28	MIX 5*x* + 4*x*	11
	56	99	47	MIX 5*x* + 4*x*	21
	57	5	9	MIX 5*x* + 4*x*	4
	57	9	5	MIX 4*x* + 5*x*	4
	57	27	77	MIX 5*x* + 4*x*	3
	57	50	26	MIX 4*x* + 5*x*	1
	57	77	27	MIX 4*x* + 5*x*	6
	58	21	100	MIX 4*x* + 5*x*	7
	58	21	100	MIX 4*x* + 5*x*	0
	58	29	96	MIX 4*x* + 5*x*	16
	58	96	29	MIX 5*x* + 4*x*	10
	58	100	21	MIX 5*x* + 4*x*	4
	60	9	35	MIX 4*x* + 5*x*	8
	60	22	25	MIX 4*x* + 5*x*	6
	60	25	22	MIX 5*x* + 4*x*	9
	60	35	9	MIX 5*x* + 4*x*	1
	60	47	97	MIX 4*x* + 5*x*	14
	60	97	47	MIX 5*x* + 4*x*	17

Table S2. Fixed effect estimates of zero-inflated Poisson Generalized Linear Mixed Models relating the seed yield of sexual tetraploid *Potentilla puberula* individuals to three pollination treatments applied in *a*n ex situ pollination experiment. The first treatment given in the comparison of treatments was used as baseline. Per each comparison, models were calculated both with the data pooled and unpooled. Significant differences ($\alpha = 0.05$) between the treatments are highlighted by bold *p*-values.

Comparison of treatments	Data	Estimate	±SE	z value	p value
Homoploid ↔ Heteroploid	pooled	–0.33	0.04	–7.827	**<0.001**
Homoploid ↔ Heteroploid pentaploid	unpooled	–0.6194	0.1001	–6.189	**<0.001**
Homoploid ↔ Heteroploid heptaploid	unpooled	–0.841	0.133	–6.324	**<0.001**
Mixed-ploidy ↔ Homoploid	pooled	0.31	0.08	3.73	**<0.001**
Mixed-ploidy pentaploid ↔ Homoploid	unpooled	0.33905	0.09901	3.424	**<0.001**
Mixed-ploidy heptaploid ↔ Homoploid	unpooled	0.3233	0.1212	2.668	**0.007**
Heteroploid pentaploid ↔ Heteroploid heptaploid	unpooled	–0.01182	0.17468	–0.068	0.946
Mixed-ploidy pentaploid ↔ Mixed-ploidy heptaploid	unpooled	–0.3062	0.1749	–1.751	0.08
Mixed-ploidy ↔ Heteroploid	pooled	–0.35	0.08	–4.34	**<0.001**
Mixed-ploidy pentaploid ↔ Heteroploid pentaploid	unpooled	–0.1842	0.1043	–1.766	0.077
Mixed-ploidy heptaploid ↔ Heteroploid heptaploid	unpooled	–0.4715	0.1558	–3.026,000	**0.002**
Mixed-ploidy ↔ (Homoploid, Heteroploid)	pooled	0.01	0.02	0.33	0.738
Mixed-ploidy pentaploid ↔ (Homoploid, Heteroploid pentaploid)	unpooled	–0.0175	0.0249	–0.703	0.482
Mixed-ploidy heptaploid ↔ (Homoploid, Heteroploid heptaploid)	unpooled	0.01788	0.0489	0.365	0.715

Table S3. Descriptive statistics of pollen grains stained and counted. Four counts were made in 2 µl aliquot of water for two anthers of the same individual. The values were averaged for the eight counts for the stained and non-stained pollen grains and calculated for the initial volume of 50 µl. The number of stained and non-stained pollen grains per flower was then multiplied by the number of anthers of the same individual, and the pollen quality percentage calculated.

Population	Individual	Ploidy	Number anthers/ flower	Stained grains	Non-stained grains	Total counted grains	Average stained grains in 2 µl	Average non-stained grains in 2 µl	Stained grains per anther in 50 µl	Non-stained grains per anther in 50 µl	Stained grains per flower	Non-stained grains per flower	Pollen quality (%)
Zabernig	56	5	18	388	74	462	48.5	9.25	1,212.5	231.25	21,825	41,62.5	83.98
	57	5	18	392	10	402	49	1.25	1,225	31.25	22,050	562.5	97.51
	60	5	na	na	na	na	na	na	na	na	na	na	na
	61	5	18	107	6	113	13.375	0.75	334.375	18.75	6,018.75	337.5	94.69
	69	5	20	268	36	304	33.5	4.5	837.5	112.5	16,750	2,250	88.16
	71	5	na	na	na	na	na	na	na	na	na	na	na
	88	5	18	693	17	710	86.625	2.125	2,165.625	53.125	38,981.25	956.25	97.61
	94	5	na	na	na	na	na	na	na	na	na	na	na
	105	5	18	184	17	201	23	2.125	575	53.125	10,350	956.25	91.54
	106	5	na	na	na	na	na	na	na	na	na	na	na
	110	5	20	89	24	113	11.125	3	278.125	75	5,562.5	1500	78.76
	111	4	na	na	na	na	na	na	na	na	na	na	na
	119	4	20	590	89	679	73.75	11.125	1,843.75	278.125	36,875	5,562.5	86.89
	120	4	na	na	na	na	na	na	na	na	na	na	na
	129	4	na	na	na	na	na	na	na	na	na	na	na
	130	4	24	483	30	513	60.375	3.75	1,509.375	93.75	36,225	2,250	94.15
	136	4	18	798	13	811	99.75	1.625	2,493.75	40.625	44,887.5	731.25	98.40
	147	4	18	798	5	803	99.75	0.625	2,493.75	15.625	44,887.5	281.25	99.38
	148	4	18	1,085	330	1415	135.625	41.25	3,390.625	1031.25	61,031.25	18,562.5	76.68
Obersteiner	31	4	na	na	na	na	na	na	na	na	na	na	na
	35	4	18	211	3	214	26.375	0.375	659.375	9.375	11,868.75	168.75	98.60
	36	5	20	268	71	339	33.5	8.875	837.5	221.875	16,750	4,437.5	79.06
	38	5	16	139	25	164	17.375	3.125	434.375	78.125	6,950	1,250	84.76
	41	5	18	379	11	390	47.375	1.375	1,184.375	34.375	21,318.75	618.75	97.18
	43	4	16	265	6	271	33.125	0.75	828.125	18.75	13,250	300	97.79
	44	4	18	502	2	504	62.75	0.25	1,568.75	6.25	28,237.5	112.5	99.60
	45	4	18	342	19	361	42.75	2.375	1,068.75	59.375	19,237.5	1,068.75	94.74
	46	4	20	740	10	750	92.5	1.25	2,312.5	31.25	46,250	625	98.67
	47	4	16	576	1	577	72	0.125	1,800	3.125	28,800	50	99.83
	48	4	20	802	17	819	100.25	2.125	2,506.25	53.125	50,125	1,062.5	97.92
	54	5	16	139	0	139	17.375	0	434.375	0	6,950	0	100.00

Table S3. Continued.

Population	Individual	Ploidy	Number anthers/ flower	Stained grains	Non-stained grains	Total counted grains	Average stained grains in 2 µl	Average non-stained grains in 2 µl	Stained grains per anther in 50 µl	Non-stained grains per anther in 50 µl	Stained grains per flower	Non-stained grains per flower	Pollen quality (%)
Obersteiner *(continued)*	57	5	na	na	na	na	na	na	na	na	na	na	na
	58	5	16	289	11	300	36.125	1.375	903.125	34.375	14,450	550	96.33
	96	4	na	na	na	na	na	na	na	na	na	na	na
	103	4	16	408	32	440	51	4	1,275	100	20,400	1,600	92.73
	104	4	na	na	na	na	na	na	na	na	na	na	na
	106	4	20	326	9	335	40.75	1.125	1,018.75	28.125	20,375	562.5	97.31
	111	5	22	358	40	398	44.75	5	1,118.75	125	24,612.5	2,750	89.95
	121	5	20	153	2	155	19.125	0.25	478.125	6.25	9,562.5	125	98.71
	125	5	18	212	17	229	26.5	2.125	662.5	53.125	11,925	956.25	92.58
	126	4	14	234	9	243	29.25	1.125	731.25	28.125	10,237.5	393.75	96.30
	127	4	20	408	7	415	51	0.875	1,275	21.875	25,500	437.5	98.31
	133	5	14	294	14	308	36.75	1.75	918.75	43.75	12,862.5	612.5	95.45
	138	5	na	na	na	na	na	na	na	na	na	na	na
	139	4	22	106	0	106	13.25	0	331.25	0	7,287.5	0	100.00
	140	4	16	1,030	6	1,036	128.75	0.75	3,218.75	18.75	51,500	300	99.42
	145	5	18	501	6	507	62.625	0.75	1,565.625	18.75	28,181.25	337.5	98.82
	148	5	16	246	9	255	30.75	1.125	768.75	28.125	12,300	450	96.47
Ossenigo/ Scaiola	4	4	20	347	3	350	43.375	0.375	1,084.375	9.375	21,687.5	187.5	99.14
	12	4	20	485	113	598	60.625	14.125	1,515.625	353.125	30,312.5	7,062.5	81.10
	13	4	20	699	16	715	87.375	2	2,184.375	50	43,687.5	1,000	97.76
	14	4	na	na	na	na	na	na	na	na	na	na	na
	16	4	20	712	100	812	89	12.5	2,225	312.5	44,500	6,250	87.68
	17	4	na	na	na	na	na	na	na	na	na	na	na
	18	4	20	1,334	1	1,335	166.75	0.125	4,168.75	3.125	83,375	62.5	99.93
	20	4	20	309	30	339	38.625	3.75	965.625	93.75	19,312.5	1,875	91.15
	21	4	16	483	193	676	60.375	24.125	1,509.375	603.125	24,150	9,650	71.45
	22	4	na	na	na	na	na	na	na	na	na	na	na
	32	4	18	593	1	594	74.125	0.125	1,853.125	3.125	33,356.25	56.25	99.83
	34	4	18	610	120	730	76.25	15	1,906.25	375	34,312.5	6,750	83.56
	49	4	20	207	3	210	43.125	0.375	1,078.125	9.375	21,562.5	187.5	99.14
	55	4	na	na	na	na	na	na	na	na	na	na	na
	5	7	20	196	15	211	24.5	1.875	612.5	46.875	12,250	937.5	92.89
	6	7	18	20	3	23	2.5	0.375	62.5	9.375	1,125	168.75	86.96

Table S3. Continued.

Population	Individual	Ploidy	Number anthers/ flower	Stained grains	Non-stained grains	Total counted grains	Average stained grains in 2 µl	Average non-stained grains in 2 µl	Stained grains per anther in 50 µl	Non-stained grains per anther in 50 µl	Stained grains per flower	Non-stained grains per flower	Pollen quality (%)
Ossenigo/ Scaiola *(continued)*	7	5	18	135	104	239	16.875	13	421.875	325	7,593.75	5,850	56.49
	8	7	20	888	33	921	111	4.125	2,775	103.125	55,500	2,062.5	96.42
	10	7	18	122	25	147	15.25	3.125	381.25	78.125	6,862.5	1,406.25	82.99
	13	7	20	96	11	107	12	1.375	300	34.375	6,000	687.5	89.72
	16	7	18	318	4	322	39.75	0.5	993.75	12.5	17,887.5	225	98.76
	21	5	20	134	14	148	16.75	1.75	418.75	43.75	8,375	875	90.54
	22	5	18	478	13	491	59.75	1.625	1,493.75	40.625	26,887.5	731.25	97.35
	27	5	18	84	10	94	10.5	1.25	262.5	31.25	4,725	562.5	89.36
	29	5	16	65	22	87	8.125	2.75	203.125	68.75	3,250	1,100	74.71
	30	5	18	169	1	170	21.125	0.125	528.125	3.125	9,506.25	56.25	99.41
	31	5	18	203	1	204	25.375	0.125	634.375	3.125	11,418.75	56.25	99.51
	32	5	na	na	na	na	na	na	na	na	na	na	na
	33	5	20	240	13	253	30	1.625	750	40.625	15,000	812.5	94.86
	34	5	18	223	11	234	27.875	1.375	696.875	34.375	12,543.75	618.75	95.30
	35	5	18	168	15	183	21	1.875	525	46.875	9,450	843.75	91.80
	36	5	na	na	na	na	na	na	na	na	na	na	na
	37	5	20	356	8	364	44.5	1	1,112.5	25	22,250	500	97.80
	38	5	18	99	9	108	12.375	1.125	309.375	28.125	5,568.75	506.25	91.67
	41	5	18	108	5	113	13.5	0.625	337.5	15.625	6,075	281.25	95.58
	42	7	na	na	na	na	na	na	na	na	na	na	na
	45	7	18	287	56	343	35.875	7	896.875	175	16,143.75	3,150	83.67
	46	7	18	354	3	357	44.25	0.375	1,106.25	9.375	19,912.5	168.75	99.16
	48	7	na	na	na	na	na	na	na	na	na	na	na
	54	7	18	329	11	340	41.125	1.375	1,028.125	34.375	18,506.25	618.75	96.76
	55	7	22	490	18	508	61.25	2.25	1,531.25	56.25	33,687.5	1,237.5	96.46
	57	7	20	527	28	555	65.875	3.5	1,646.875	87.5	32,937.5	1,750	94.95
	63	7	na	na	na	na	na	na	na	na	na	na	na
	65	5	na	na	na	na	na	na	na	na	na	na	na
	70	7	22	190	5	195	23.75	0.625	593.75	15.625	13,062.5	343.75	97.44
	71	7	18	24	6	30	3	0.75	75	18.75	1,350	337.5	80.00
	74	7	16	77	28	105	9.625	3.5	240.625	87.5	3,850	1,400	73.33
	78	7	20	546	13	559	68.25	1.625	1,706.25	40.625	34,125	812.5	97.67
	80	7	22	87	30	117	10.875	3.75	271.875	93.75	5,981.25	2,062.5	74.36

Table S3. Continued.

Population	Individual	Ploidy	Number anthers/ flower	Stained grains	Non-stained grains	Total counted grains	Average stained grains in 2 µl	Average non-stained grains in 2 µl	Stained grains per anther in 50 µl	Non-stained grains per anther in 50 µl	Stained grains per flower	Non-stained grains per flower	Pollen quality (%)
Ossenigo/ Scaiola *(continued)*	84	7	22	808	24	832	101	3	2,525	75	55,550	1,650	97.12
	86	5	16	219	1	220	27.375	0.125	684.375	3.125	10,950	50	99.55
	90	5	16	337	39	376	42.125	4.875	1,053.125	121.875	16,850	1,950	89.63
	91	5	16	210	53	263	26.25	6.625	656.25	165.625	10,500	2,650	79.85
	95	5	na	na	na	na	na	na	na	na	na	na	na
	100	7	20	120	2	122	15	0.25	375	6.25	7,500	125	98.36
	117	5	18	252	55	307	31.5	6.875	787.5	171.875	14,175	3,093.75	82.08
	120	5	na	na	na	na	na	na	na	na	na	na	na
Raas/ Fortezza	1	7	21	1	0	1	0.125	0	3.125	0	65.625	0	100.00
	3	5	17	142	102	244	17.75	12.75	443.75	318.75	7,543.75	5,418.75	58.20
	4	7	18	113	40	153	14.125	5	353.125	125	6,356.25	2,250	73.86
	8	5	16	122	34	156	15.25	4.25	381.25	106.25	6,100	1,700	78.21
	9	7	21	52	95	147	6.5	11.875	162.5	296.875	3,412.5	6,234.375	35.37
	11	7	14	45	13	58	5.625	1.625	140.625	40.625	1,968.75	568.75	77.59
	12	5	19	448	120	568	56	15	1,400	375	26,600	7,125	78.87
	13	5	18	657	30	687	82.125	3.75	2,053.125	93.75	36,956.25	1,687.5	95.63
	14	7	20	186	66	252	23.25	8.25	581.25	206.25	11,625	4,125	73.81
	16	7	19	46	8	54	5.75	1	143.75	25	2,731.25	475	85.19
	18	7	na	na	na	na	na	na	na	na	na	na	na
	19	5	17	312	132	444	39	16.5	975	412.5	16,575	7,012.5	70.27
	23	7	19	498	8	506	62.25	1	1,556.25	25	29,568.75	475	98.42
	24	5	18	270	85	355	33.75	10.625	843.75	265.625	15,187.5	4,781.25	76.06
	25	5	20	486	28	514	60.75	3.5	1,518.75	87.5	30,375	1,750	94.55
	26	7	18	411	3	414	51.375	0.375	1,284.375	9.375	23,118.75	168.75	99.28
	27	7	18	108	20	128	13.5	2.5	337.5	62.5	6,075	1,125	84.38
	28	5	19	51	13	64	6.375	1.625	159.375	40.625	3,028.125	771.875	79.69
	29	7	18	199	90	289	24.875	11.25	621.875	281.25	11,193.75	5,062.5	68.86
	31	5	20	133	8	141	16.625	1	415.625	25	8,312.5	500	94.33
	32	5	19	435	44	479	54.375	5.5	1,359.375	137.5	25,828.125	2,612.5	90.81
	33	5	18	461	12	473	57.625	1.5	1,440.625	37.5	25,931.25	675	97.46
	37	7	18	75	2	77	9.375	0.25	234.375	6.25	4,218.75	112.5	97.40
	41	7	20	69	14	83	8.625	1.75	215.625	43.75	4,312.5	875	83.13
	43	7	23	124	50	174	15.5	6.25	387.5	156.25	8,912.5	3,593.75	71.26

Table S3. Continued.

Population	Individual	Ploidy	Number anthers/ flower	Stained grains	Non-stained grains	Total counted grains	Average stained grains in 2 µl	Average non-stained grains in 2 µl	Stained grains per anther in 50 µl	Non-stained grains per anther in 50 µl	Stained grains per flower	Non-stained grains per flower	Pollen quality (%)
Raas/ Fortezza *(continued)*	44	5	20	575	75	650	71.875	9.375	1,796.875	234.375	35,937.5	4,687.5	88.46
	47	5	20	769	16	785	96.125	2	2,403.125	50	48,062.5	1,000	97.96
	48	5	20	755	24	779	94.375	3	2,359.375	75	47,187.5	1,500	96.92
	49	7	20	11	0	11	1.375	0	34.375	0	687.5	0	100.00
	50	7	18	86	74	160	10.75	9.25	268.75	231.25	4,837.5	4,162.5	53.75
	51	7	16	128	2	130	16	0.25	400	6.25	6,400	100	98.46
	53	7	22	73	30	103	9.125	3.75	228.125	93.75	5,018.75	2,062.5	70.87
	59	7	20	106	32	138	13.25	4	331.25	100	6,625	2,000	76.81
	60	5	19	420	62	482	52.5	7.75	1,312.5	193.75	24,937.5	3,681.25	87.14
	63	7	18	205	36	241	25.625	4.5	640.625	112.5	11,531.25	2,025	85.06
	65	7	16	100	13	113	12.5	1.625	312.5	40.625	5,000	650	88.50
	66	5	20	627	34	661	78.375	4.25	1,959.375	106.25	39,187.5	2,125	94.86
	67	7	15	80	0	80	10	0	250	0	3,750	0	100.00
	73	7	16	76	8	84	9.5	1	237.5	25	3,800	400	90.48
	2	4	18	1,010	14	1,024	126.25	1.75	3,156.25	43.75	56,812.5	787.5	98.63
	3	4	18	947	10	957	118.375	1.25	2,959.375	31.25	53,268.75	562.5	98.96
	5	4	18	684	12	696	85.5	1.5	2,137.5	37.5	38,475	675	98.28
	6	4	22	785	6	791	98.125	0.75	2,453.125	18.75	53,968.75	412.5	99.24
	8	4	na	na	na	na	na	na	na	na	na	na	na
	9	4	na	na	na	na	na	na	na	na	na	na	na
	10	4	16	509	110	619	63.625	13.75	1,590.625	343.75	25,450	5,500	82.23
	11	4	na	na	na	na	na	na	na	na	na	na	na
	12	4	18	513	13	526	64.125	1.625	1,603.125	40.625	28,856.25	731.25	97.53
	13	4	20	514	8	522	64.25	1	1,606.25	25	32,125	500	98.47
	29	4	18	482	4	486	60.25	0.5	1,506.25	12.5	27,112.5	225	99.18
	31	4	na	na	na	na	na	na	na	na	na	na	na
	33	4	20	505	20	525	63.125	2.5	1,578.125	62.5	31,562.5	1,250	96.19
	35	4	na	na	na	na	na	na	na	na	na	na	na
	44	4	na	na	na	na	na	na	na	na	na	na	na
	52	4	18	739	22	761	92.375	2.75	2,309.375	68.75	41,568.75	1,237.5	97.11
Bodenalm	2	5	14	387	38	425	48.375	4.75	1,209.375	118.75	16,931.25	1,662.5	91.06
	4	4	na	na	na	na	na	na	na	na	na	na	na
	5	5	18	158	1	159	19.75	0.125	493.75	3.125	8,887.5	56.25	99.37

Table S3. Continued.

Population	Individual	Ploidy	Number anthers/ flower	Stained grains	Non-stained grains	Total counted grains	Average stained grains in 2 µl	Average non-stained grains in 2 µl	Stained grains per anther in 50 µl	Non-stained grains per anther in 50 µl	Stained grains per flower	Non-stained grains per flower	Pollen quality (%)
Bodenalm *(continued)*	6	4	20	141	33	174	17.625	4.125	440.625	103.125	8,812.5	2,062.5	81.03
	8	4	18	404	2	406	50.5	0.25	1,262.5	6.25	22,725	112.5	99.51
	9	4	16	57	12	69	7.125	1.5	178.125	37.5	2,850	600	82.61
	10	5	14	577	105	682	72.125	13.125	1,803.125	328.125	25,243.75	4,593.75	84.60
	15	5	16	224	1	225	28	0.125	700	3.125	11,200	50	99.56
	18	4	18	109	3	112	13.625	0.375	340.625	9.375	6,131.25	168.75	97.32
	20	4	16	502	22	524	62.75	2.75	1,568.75	68.75	25,100	1,100	95.80
	21	4	16	583	3	586	72.875	0.375	1,821.875	9.375	29,150	150	99.49
	22	4	20	121	34	155	15.125	4.25	378.125	106.25	7,562.5	2,125	78.06
	25	5	18	130	5	135	16.25	0.625	406.25	15.625	7,312.5	281.25	96.30
	26	5	20	210	0	210	26.25	0	656.25	0	13,125	0	100.00
	27	5	18	790	14	804	98.75	1.75	2,468.75	43.75	44,437.5	787.5	98.26
	28	4	19	96	11	107	12	1.375	300	34.375	5,700	653.125	89.72
	29	4	18	356	7	363	44.5	0.875	1,112.5	21.875	20,025	393.75	98.07
	30	4	14	437	4	441	54.625	0.5	1,365.625	12.5	19,118.75	175	99.09
	35	5	18	177	1	178	22.125	0.125	553.125	3.125	9,956.25	56.25	99.44
	36	4	na	na	na	na	na	na	na	na	na	na	na
	37	4	18	232	3	235	29	0.375	725	9.375	13,050	168.75	98.72
	47	4	16	253	10	263	31.625	1.25	790.625	31.25	12,650	500	96.20
	49	4	14	141	3	144	17.625	0.375	440.625	9.375	6,168.75	131.25	97.92
	50	4	na	na	na	na	na	na	na	na	na	na	na
	69	5	18	391	57	448	48.875	7.125	1,221.875	178.125	21,993.75	3,206.25	87.28
	77	4	20	182	6	188	22.75	0.75	568.75	18.75	11,375	375	96.81
	96	5	18	455	18	473	56.875	2.25	1,421.875	56.25	25,593.75	1,012.5	96.19
	97	5	20	214	3	217	26.75	0.375	668.75	9.375	13,375	187.5	98.62
	99	5	18	892	20	912	111.5	2.5	2,787.5	62.5	50,175	1,125	97.81
	100	5	16	533	28	561	66.625	3.5	1,665.625	87.5	26,650	1,400	95.01

Table S4. Descriptive statistics of the flow cytometric measurement of seeds of *Potentilla puberula* Krašan. For each “pollen recipient” and “pollen donor” used in the crosses the “Population”, and “Individual” number is provided. The internal biological standard was for all measurements *Pisum sativum* ‘Kleine Rheinländerin’. “Count”, “Mean” and “CV” are the number of particles registered, the mean fluorescence, and the variation coefficient, respectively, calculated for the internal “Standard” and the “Embryo”. A) 35 seeds from homoploid tetraploid fertilizations, B) 67 seeds from heteroploid fertilizations and C) 871 seeds from mixed-ploidy fertilizations. Population numbers refer to the studied regions as follows: Zabernig 10, Obersteiner 45, Ossenigo/Scaiola 64/68, Raas/Fortezza 121/119 and Bodenalm 144.

A) Homoploid tetraploid fertilization seeds

Pollen recipient			Pollen donor			Standard			Embryo			Embryo : Standard
Population	Individual	Ploidy [*x*]	Population	Individual	Ploidy [*x*]	Count	Mean	CV	Count	Mean	CV	ratio
10	125	4	10	142	4	573	705.98	3.07	413	99.96	5.21	0.141590
45	103	4	45	43	4	389	715.67	2.79	701	102.17	5.29	0.142761
45	103	4	45	43	4	343	704.00	2.95	946	102.62	6.31	0.145767
45	103	4	45	44	4	785	712.7	1.9	373	102.02	5.08	0.143145
45	103	4	45	44	4	846	711.87	2.26	770	101.4	3.82	0.142442
45	104	4	45	140	4	211	707.76	3.80	964	100.54	4.42	0.142054
45	104	4	45	140	4	539	727.67	2.74	929	102.96	5.60	0.141493
45	116	4	45	45	4	806	712.47	2.62	616	100.06	4.81	0.140441
45	116	4	45	45	4	437	724.71	2.80	753	103.53	5.23	0.142857
45	116	4	45	45	4	637	709.69	2.76	454	100.28	5.96	0.141301
45	116	4	45	45	4	397	709.52	2.80	778	101.77	6.91	0.143435
45	146	4	45	127	4	747	710.55	1.8	1512	101.27	4.48	0.142523
45	146	4	45	127	4	517	727.33	1.93	715	101.79	3.34	0.139950
64	18	4	64	14	4	587	692.65	2.46	710	97.44	4.52	0.140677
64	18	4	64	14	4	293	713.84	2.93	926	99.55	4.62	0.139457
64	18	4	64	12	4	532	706.47	1.98	1132	99.98	4.06	0.141520
64	18	4	64	12	4	402	709.84	2.01	1170	100.05	3.54	0.140947
64	18	4	64	12	4	302	707.03	1.97	986	98.97	3.19	0.139979
64	27	4	64	20	4	1052	715.16	2.45	1032	103,68	4,05	0.144974
64	48	4	64	55	4	653	715.2	2.36	718	101.98	4.36	0.142589
64	48	4	64	55	4	214	719.33	3.67	1425	104.26	5.18	0.144940
121	20	4	121	10	4	409	707.22	2.56	869	104.16	6.24	0.147281
121	20	4	121	10	4	463	714.43	2.88	1075	101.18	4.54	0.141623
121	20	4	121	10	4	266	714.13	2.60	612	100.47	3.90	0.140689
121	22	4	121	52	4	600	710.64	2.19	1716	99.74	3.88	0.140352
121	22	4	121	52	4	635	705.71	2.45	927	100.07	3.73	0.141800
121	40	4	121	3	4	514	721.40	2.82	678	100.13	4.41	0.138800
121	40	4	121	6	4	685	715.03	1.92	702	100.1	4.11	0.139994
121	40	4	121	6	4	551	713.26	2.42	417	101.44	4.75	0.142220
121	40	4	121	3	4	328	724.60	3.17	847	102.67	5.48	0.141692
144	29	4	144	6	4	604	713.67	6.21	805	102.23	6.67	0.143245
144	29	4	144	20	4	780	728.66	3.17	459	98.14	5.16	0.134686
144	29	4	144	20	4	810	727.18	3.09	1358	101.61	5.22	0.139732
144	37	4	144	30	4	755	708.84	1.67	463	99.51	3.68	0.140384
144	37	4	144	30	4	727	729.86	1.8	917	101.01	3.98	0.13839

Table S4. Continued,

B) Heteroploid fertilizations seeds

Pollen recipient			Pollen donor			Standard			Embryo			Embryo : Standard ratio
Population	Individual	Ploidy [*x*]	Population	Individual	Ploidy [*x*]	Count	Mean	CV	Count	Mean	CV	
10	140	4	10	56	5	185	735.79	2.53	334	103.81	4.25	0.141086
10	140	4	10	56	5	723	699.04	1.58	951	100.69	4.88	0.144040
45	103	4	45	143	5	687	730.13	1.94	592	104.03	4.26	0.142481
45	103	4	45	145	5	287	716.87	3.58	627	107.18	4.94	0.149511
45	103	4	45	145	5	444	721.06	3.52	356	117.97	7.03	0.163606
45	105	4	45	41	5	1147	709.25	3.94	1026	109.14	4.53	0.153881
45	106	4	45	121	5	649	718.41	2.89	925	117.07	4.71	0.162957
45	106	4	45	121	5	606	725.01	2.75	898	136.81	3.56	0.188701
45	109	4	45	41	5	674	716.1	2.9	1103	119.14	3.56	0.166373
45	109	4	45	41	5	672	722.84	3.05	476	128.16	4.36	0.177301
45	116	4	45	36	5	538	698.03	1.77	861	96.85	3.72	0.138747
45	116	4	45	58	5	1627	716.72	2.84	592	104.88	3.57	0.146333
45	116	4	45	58	5	400	700.61	2.42	550	114.51	3.82	0.163443
45	143	4	45	121	5	571	691.24	2.25	1332	99.69	5.7	0.144219
45	143	4	45	121	5	531	719.75	2.86	593	114.6	5.69	0.159222
45	146	4	45	111	5	248	721.46	1.96	1176	101.5	3.89	0.140687
45	146	4	45	111	5	1145	708.02	2.42	678	103.16	3.35	0.14570
64	14	4	68	37	5	319	717.87	2.03	865	106.07	4.61	0.147756
64	14	4	68	37	5	1196	714.01	1.62	821	106.94	3.64	0.149774
64	14	4	68	37	5	649	722.31	1.89	734	120.9	4.08	0.167379
64	14	4	68	117	5	742	721.54	3.17	407	132.54	3.31	0.183690
64	14	4	68	117	5	662	716.69	1.79	618	138.81	4.39	0.193682
64	14	4	68	117	5	671	726.33	2.53	654	141.07	3.45	0.194223
121	36	4	119	12	5	520	744.59	2.16	694	130.36	3.33	0.175076
121	37	4	119	13	5	763	716.84	1.62	576	122.13	3.15	0.170373
121	40	4	119	25	5	327	724.27	1.87	858	103.83	3.48	0.143358
121	40	4	119	25	5	286	708.34	1.43	788	120.24	3.41	0.169749
121	40	4	119	25	5	263	699.52	2.79	897	123.49	4.99	0.176535
121	40	4	119	32	5	508	716.59	1.62	912	126.57	3.74	0.176628
121	43	4	119	31	5	851	707.9	2.27	963	119.44	3.46	0.168724
121	43	4	119	31	5	588	726.71	1.84	379	128.19	3.16	0.176397
144	31	4	144	10	5	689	722.24	1.9	487	105.01	4.15	0.145395
144	31	4	144	10	5	493	726.61	2.1	578	124.15	3.43	0.170862
144	37	4	144	10	5	328	730.1	1.83	310	125.97	4.82	0.172538
144	47	4	144	27	5	219	706.91	2.11	505	113.58	4.37	0.160671
144	47	4	144	27	5	472	719.45	2.06	1225	122.16	3.51	0.169796
144	50	4	144	100	5	350	709.01	2.19	922	115.56	4.25	0.162988
64	14	4	68	5	7	453	727.6	1.65	720	134.11	3.67	0.184318

Table S4. Continued,

B) Heteroploid fertilizations seeds

Pollen recipient			Pollen donor			Standard			Embryo			Embryo : Standard ratio
Population	Individual	Ploidy [*x*]	Population	Individual	Ploidy [*x*]	Count	Mean	CV	Count	Mean	CV	
64	14	4	68	5	7	487	726.23	1.75	1170	135.73	3.51	0.186896
64	14	4	68	13	7	179	701.85	1.88	703	136.34	2.99	0.194258
64	14	4	68	13	7	576	739.91	2	962	137	3.63	0.185157
64	14	4	68	13	7	438	700.65	1.8	1769	130.87	3.85	0.186783
64	14	4	68	13	7	787	711.09	1.79	1088	126.59	3.27	0.178022
64	14	4	68	71	7	448	718.58	1.79	616	143.37	4.15	0.199518
64	14	4	68	71	7	1108	712.43	1.97	288	141.83	3.62	0.199079
64	27	4	68	100	7	327	776.33	2.25	626	156.69	3.31	0.201834
64	27	4	68	100	7	452	732.43	2.21	594	139.85	3.47	0.190939
64	27	4	68	100	7	566	722.78	1.58	838	140.65	2.98	0.194596
121	20	4	119	18	7	655	713.59	2.05	726	126.66	3.86	0.177497
121	20	4	119	18	7	647	742.62	3.53	621	146.27	4.66	0.196965
121	20	4	119	18	7	555	730.22	2.15	1245	133.86	3.37	0.183314
121	20	4	119	18	7	659	703.64	1.76	1260	138.57	3.79	0.196933
121	20	4	119	18	7	817	717.12	2.35	645	147.84	3.59	0.206158
121	20	4	119	18	7	583	713.55	2.74	878	126.82	3.59	0.177731
121	20	4	119	18	7	617	716.07	1.99	818	129.63	3.2	0.181029
121	20	4	119	18	7	566	714.18	2.08	1069	145.34	3.54	0.203506
121	27	4	119	16	7	541	702.29	1.89	952	133.88	3.61	0.190633
121	27	4	119	16	7	1134	708.88	1.98	1249	142.76	3.84	0.201388
121	36	4	119	23	7	301	713.51	1.94	1602	125.79	4.73	0.176297
121	36	4	119	23	7	356	713.27	2.43	777	141.15	3.97	0.197891
121	36	4	119	29	7	920	712.75	1.97	1189	125.78	3.5	0.176471
121	36	4	119	29	7	1453	710.42	1.82	1579	142.47	3.46	0.200543
121	36	4	119	29	7	488	715.47	2.07	713	141.64	3.48	0.197967
121	37	4	119	53	7	342	700.76	2.2	891	128.26	4.14	0.183029
121	37	4	119	53	7	375	713.46	1.75	690	145.37	3.25	0.203753
121	37	4	119	53	7	273	711.08	2	798	134.35	3.48	0.188938
121	40	4	119	63	7	521	704.76	1.8	864	131.07	4.16	0.185978

C) Mixed-ploidy fertilizations seeds

Pollen recipient			Pollen donor 1			Pollen donor 2			Standard			Embryo			Embryo:Standard ratio
Population	Individual	Ploidy [*x*]	Population	Individual	Ploidy [*x*]	Population	Individual	Ploidy [*x*]	Count	Mean	CV	Count	Mean	CV	
10	140	4	10	111	4	10	106	5	758	684.58	3.34	638	96.22	5.63	0.140553332
10	140	4	10	111	4	10	106	5	744	712.85	3.22	1320	98.35	6.14	0.137967314
10	140	4	10	111	4	10	106	5	707	722.38	3.01	1442	100.66	5.54	0.139344943
10	142	4	10	147	4	10	97	5	1166	725.35	3.48	986	101.20	5.38	0.139518853
10	142	4	10	147	4	10	97	5	728	722.16	3.23	1120	102.58	5.80	0.142046084

Table S4. Continued,

C) Mixed-ploidy fertilizations seeds

Pollen recipient			Pollen donor 1			Pollen donor 2			Standard			Embryo			Embryo:Standard ratio
Population	Individual	Ploidy [*x*]	Population	Individual	Ploidy [*x*]	Population	Individual	Ploidy [*x*]	Count	Mean	CV	Count	Mean	CV	
10	142	4	10	147	4	10	97	5	1759	703.71	2.84	1674	97.89	6.74	0.139105597
10	142	4	10	147	4	10	97	5	1071	675.64	4.26	1392	100.44	6.30	0.148659049
10	142	4	10	147	4	10	97	5	1067	703.81	3.48	1679	98.57	6.43	0.140052003
10	142	4	10	147	4	10	97	5	734	726.38	3.35	1509	102.13	5.57	0.140601338
10	142	4	10	147	4	10	97	5	782	723.23	3.16	927	98.16	5.76	0.135724458
10	142	4	10	147	4	10	97	5	1085	708.16	3.20	534	136.60	5.44	0.192894261
10	142	4	10	147	4	10	97	5	1145	706.67	2.96	854	119.51	4.91	0.169117127
10	142	4	10	147	4	10	97	5	1042	710.32	3.01	1241	99.03	5.85	0.139416038
10	142	4	10	147	4	10	97	5	940	702.78	3.00	1209	99.30	6.53	0.141295996
10	142	4	10	147	4	10	97	5	1064	703.96	3.11	1394	96.89	5.55	0.137635661
45	103	4	45	43	4	45	36	5	1062	712.54	3.79	2099	101.01	6.03	0.141760463
45	103	4	45	44	4	45	145	5	1414	683.93	3.72	1818	96.38	5.96	0.140920854
45	103	4	45	44	4	45	145	5	685	709.74	3.27	1212	101.19	5.35	0.142573337
45	103	4	45	44	4	45	145	5	1145	703.32	3.01	1541	99.71	5.26	0.14177046
45	103	4	45	44	4	45	145	5	1308	682.98	3.19	1588	93.87	4.92	0.137441799
45	103	4	45	44	4	45	145	5	191	713.22	2.58	967	103.73	4.86	0.145438995
45	103	4	45	44	4	45	145	5	1679	671.50	3.11	1974	93.06	5.63	0.138585257
45	103	4	45	44	4	45	145	5	1139	729.05	2.20	1262	105.70	5.31	0.144983197
45	103	4	45	44	4	45	145	5	674	687.09	3.83	1863	102.46	7.59	0.149121658
45	103	4	45	36	5	45	43	4	1999	697.19	3.24	1370	98.46	5.91	0.141224057
45	103	4	45	36	5	45	43	4	1390	724.09	2.79	857	112.09	6.36	0.154801199
45	103	4	45	36	5	45	43	4	1974	712.14	3.43	839	101.67	5.62	0.142766872
45	103	4	45	36	5	45	43	4	1258	717.38	2.81	822	104.33	5.34	0.145431989
45	103	4	45	36	5	45	43	4	1162	719.21	4.11	2108	104.54	5.45	0.14535393
45	103	4	45	145	5	45	44	4	1136	700.30	2.96	2584	98.72	6.11	0.140968157
45	103	4	45	145	5	45	44	4	988	753.29	3.00	909	109.09	5.23	0.144818065
45	103	4	45	145	5	45	44	4	2193	720.54	3.22	1145	101.37	6.38	0.140686152
45	103	4	45	145	5	45	44	4	182	718.46	2.88	1940	101.21	6.24	0.140870751
45	103	4	45	44	4	45	145	5	887	743.04	3.51	1591	103.61	5.06	0.139440676
45	103	4	45	44	4	45	145	5	1795	713.29	2.45	1295	101.29	5.24	0.142003954
45	103	4	45	44	4	45	145	5	268	723.22	3.33	1045	145.27	4.63	0.200865573
45	103	4	45	44	4	45	145	5	1347	714.86	2.21	1516	101.18	4.97	0.141538203
45	103	4	45	145	5	45	44	4	289	724.15	3.08	1359	102.27	4.85	0.141227646
45	103	4	45	145	5	45	44	4	2004	710.57	2.66	975	100.75	4.19	0.141787579
45	103	4	45	145	5	45	44	4	450	714.68	2.71	2642	99.36	5.69	0.139027257
45	103	4	45	145	5	45	44	4	615	708.02	3.13	875	98.25	3.86	0.138767266
45	104	4	45	44	4	45	58	5	186	692.68	2.67	1416	96.90	4.60	0.139891436
45	104	4	45	44	4	45	58	5	598	743.59	2.33	1618	107.61	5.28	0.144716847

Table S4. Continued,

C) Mixed-ploidy fertilizations seeds

Pollen recipient			Pollen donor 1			Pollen donor 2			Standard			Embryo			Embryo:Standard ratio
Population	Individual	Ploidy [*x*]	Population	Individual	Ploidy [*x*]	Population	Individual	Ploidy [*x*]	Count	Mean	CV	Count	Mean	CV	
45	104	4	45	44	4	45	58	5	160	712.50	3.06	989	127.27	3.73	0.178624561
45	104	4	45	127	4	45	54	5	499	707.18	2.95	1658	103.02	4.74	0.145677197
45	104	4	45	127	4	45	54	5	917	716.15	3.18	1661	102.62	4.49	0.143294003
45	104	4	45	127	4	45	54	5	768	751.18	2.26	1642	105.74	4.79	0.140765196
45	104	4	45	140	4	45	145	5	239	709.76	3.48	1400	130.24	4.49	0.183498647
45	104	4	45	140	4	45	145	5	477	735.69	2.28	1523	105.00	4.04	0.142723158
45	104	4	45	140	4	45	145	5	283	718.05	2.46	1061	123.71	4.46	0.172286053
45	104	4	45	140	4	45	145	5	561	712.50	2.62	1531	120.15	4.38	0.168631579
45	104	4	45	58	5	45	44	4	702	722.69	2.96	1641	104.62	4.99	0.144764699
45	104	4	45	58	5	45	44	4	880	741.93	2.88	1649	107.09	4.94	0.144339763
45	104	4	45	54	5	45	127	4	693	729.41	3.05	1986	104.73	5.35	0.143581799
45	104	4	45	54	5	45	127	4	252	748.01	2.53	1135	106.92	4.24	0.142939266
45	104	4	45	54	5	45	127	4	210	721.05	2.55	1085	103.61	4.90	0.143693225
45	104	4	45	54	5	45	127	4	138	724.80	3.13	2461	104.77	5.09	0.144550221
45	104	4	45	54	5	45	127	4	632	748.72	2.50	1099	105.93	4.60	0.141481462
45	104	4	45	145	5	45	140	4	623	737.47	2.71	2772	105.19	5.24	0.142636311
45	104	4	45	145	5	45	140	4	1236	718.35	3.31	1758	101.85	4.95	0.141783253
45	104	4	45	145	5	45	140	4	1587	727.07	2.61	1862	102.62	4.61	0.141141843
45	105	4	45	106	4	45	41	5	1343	689.49	3.81	2288	96.04	6.15	0.13929136
45	105	4	45	106	4	45	41	5	926	706.67	2.44	1005	101.63	4.04	0.143815359
45	105	4	45	106	4	45	41	5	992	680.29	3.34	2174	95.62	6.16	0.140557703
45	105	4	45	106	4	45	41	5	669	729.16	2.63	865	102.81	3.47	0.140997861
45	105	4	45	47	4	45	38	5	364	700.24	3.17	1395	93.91	5.15	0.134111162
45	105	4	45	47	4	45	38	5	454	713.68	3.08	1652	99.29	5.25	0.139123977
45	105	4	45	41	5	45	106	4	207	720.27	2.53	2316	99.94	5.09	0.138753523
45	105	4	45	41	5	45	106	4	971	732.29	2.63	1271	102.70	5.43	0.140244985
45	105	4	45	41	5	45	106	4	344	713.07	2.64	1397	100.30	4.23	0.140659402
45	105	4	45	41	5	45	106	4	1551	770.66	2.61	821	108.67	4.83	0.141009005
45	105	4	45	38	5	45	47	4	299	715.68	4.50	2221	97.12	5.61	0.135703108
45	105	4	45	38	5	45	47	4	984	712.40	3.07	793	101.05	4.21	0.141844469
45	105	4	45	38	5	45	47	4	575	693.29	3.86	2618	92.90	6.87	0.13399876
45	105	4	45	38	5	45	47	4	1105	716.15	2.86	1646	101.41	4.94	0.141604412
45	106	4	45	46	4	45	33	5	767	707.91	4.11	2277	100.39	5.33	0.141811812
45	106	4	45	46	4	45	33	5	362	748.27	3.55	948	105.93	4.50	0.141566547
45	106	4	45	121	5	45	35	4	843	754.70	3.24	2038	103.29	5.05	0.136862329
45	106	4	45	121	5	45	35	4	990	755.12	2.77	1227	105.32	4.48	0.139474521
45	106	4	45	121	5	45	35	4	429	763.55	3.97	1633	115.14	5.63	0.150795626
45	106	4	45	121	5	45	35	4	1745	805.80	3.22	1284	118.45	4.96	0.146996773

Table S4. Continued,

C) Mixed-ploidy fertilizations seeds

Pollen recipient			Pollen donor 1			Pollen donor 2			Standard			Embryo			Embryo:Standard ratio
Population	Individual	Ploidy [*x*]	Population	Individual	Ploidy [*x*]	Population	Individual	Ploidy [*x*]	Count	Mean	CV	Count	Mean	CV	
45	106	4	45	139	5	45	48	4	423	734.99	3.44	1870	104.56	5.85	0.142260439
45	106	4	45	139	5	45	48	4	1367	746.94	2.73	1452	105.04	4.32	0.140627092
45	106	4	45	139	5	45	48	4	278	723.72	3.88	1446	104.15	4.84	0.143909247
45	106	4	45	139	5	45	48	4	1317	762.55	2.35	1327	105.36	4.33	0.138167989
45	109	4	45	139	5	45	48	4	1012	799.12	4.40	1206	117.35	5.03	0.146849034
45	109	4	45	43	4	45	45	5	626	718.42	3.01	1112	100.37	4.18	0.139709362
45	109	4	45	43	4	45	45	5	765	727.33	3.14	1997	100.27	4.47	0.137860393
45	109	4	45	43	4	45	45	5	1133	780.69	2.84	727	112.82	4.04	0.144513187
45	109	4	45	48	4	45	139	5	544	715.44	5.21	1737	103.88	4.97	0.145197361
45	109	4	45	48	4	45	139	5	600	671.76	2.94	1036	97.22	5.03	0.144724306
45	109	4	45	48	4	45	139	5	515	717.80	4.56	1795	103.67	4.84	0.144427417
45	109	4	45	48	4	45	139	5	612	761.44	2.95	340	104.38	3.82	0.13708237
45	109	4	45	103	4	45	138	5	684	687.27	5.61	1340	113.02	4.05	0.164447743
45	109	4	45	103	4	45	138	5	836	764.85	3.03	1198	107.00	4.37	0.139896712
45	109	4	45	103	4	45	138	5	697	711.28	3.70	1135	100.40	4.35	0.141153976
45	109	4	45	103	4	45	138	5	1224	758.24	3.17	1219	157.57	3.34	0.207810192
45	109	4	45	41	5	45	43	4	469	728.34	2.85	1668	100.30	4.56	0.13771041
45	109	4	45	41	5	45	43	4	606	759.61	2.67	1226	104.74	3.90	0.137886547
45	109	4	45	41	5	45	43	4	805	704.52	6.70	1251	103.95	4.16	0.147547266
45	109	4	45	139	5	45	48	4	1715	726.15	3.33	451	103.13	4.90	0.142022998
45	109	4	45	139	5	45	48	4	2421	747.74	3.00	1056	103.98	4.51	0.139059031
45	109	4	45	139	5	45	48	4	1775	721.71	3.47	1011	99.97	5.09	0.138518241
45	109	4	45	138	5	45	103	4	859	676.14	6.49	542	112.29	3.93	0.166075073
45	109	4	45	138	5	45	103	4	539	731.11	2.93	2302	101.39	5.23	0.138679542
45	109	4	45	138	5	45	103	4	796	634.79	6.37	1836	110.61	4.09	0.174246601
45	109	4	45	138	5	45	103	4	335	750.50	3.89	554	106.80	5.35	0.142305130
45	110	4	45	127	4	45	145	5	371	730.03	3.69	2779	104.93	5.31	0.143733819
45	110	4	45	127	4	45	145	5	1864	729.28	2.93	1135	101.34	4.42	0.138958973
45	110	4	45	106	4	45	36	5	239	738.38	3.98	2198	107.87	5.53	0.146090089
45	110	4	45	47	4	45	58	5	791	707.85	2.82	2508	99.58	4.88	0.140679522
45	110	4	45	47	4	45	58	5	978	731.39	2.66	1817	105.46	5.46	0.144191198
45	110	4	45	47	4	45	58	5	854	710.79	2.40	1513	101.50	4.53	0.142798858
45	110	4	45	47	4	45	58	5	1657	710.56	4.73	1409	100.16	5.95	0.140959243
45	110	4	45	47	4	45	58	5	1223	723.10	2.81	1119	103.21	4.99	0.142732679
45	110	4	45	145	5	45	127	4	1523	698.62	2.98	1161	99.25	5.49	0.142065787
45	110	4	45	36	5	45	106	4	1754	716.92	2.58	1734	99.71	4.09	0.139081069
45	110	4	45	36	5	45	106	4	1391	723.61	3.30	423	99.92	4.23	0.138085433
45	110	4	45	36	5	45	106	4	1478	678.14	3.38	1055	95.51	5.84	0.140841124

Table S4. Continued,

C) Mixed-ploidy fertilizations seeds

Pollen recipient			Pollen donor 1			Pollen donor 2			Standard			Embryo			Embryo:Standard ratio
Population	Individual	Ploidy [*x*]	Population	Individual	Ploidy [*x*]	Population	Individual	Ploidy [*x*]	Count	Mean	CV	Count	Mean	CV	
45	110	4	45	58	5	45	47	4	1123	735.64	2.79	1364	102.11	4.61	0.138804306
45	110	4	45	58	5	45	47	4	1478	703.36	2.87	554	99.34	5.03	0.141236351
45	110	4	45	58	5	45	47	4	1551	722.68	2.73	1883	101.16	4.65	0.139978967
45	110	4	45	58	5	45	47	4	2182	694.92	2.87	1099	96.63	4.86	0.139051977
45	113	4	45	106	4	45	111	5	1533	731.68	2.54	1973	100.54	4.09	0.137409797
45	113	4	45	46	4	45	125	5	889	714.70	3.11	1601	98.36	4.89	0.137624178
45	113	4	45	46	4	45	125	5	1171	763.78	2.85	1869	105.38	5.43	0.137971667
45	113	4	45	46	4	45	125	5	1200	736.14	3.15	2443	102.38	4.84	0.139076806
45	113	4	45	46	4	45	125	5	737	684.68	2.46	1088	99.69	5.01	0.145600865
45	113	4	45	125	5	45	46	4	2284	726.30	3.63	1036	100.44	4.84	0.138289963
45	113	4	45	125	5	45	46	4	1473	720.47	3.77	1895	100.54	4.29	0.139547795
45	116	4	45	45	4	45	148	5	1333	708.51	3.18	892	100.40	5.30	0.141705833
45	116	4	45	45	4	45	148	5	1599	748.64	3.33	535	104.06	6.40	0.138998718
45	116	4	45	45	4	45	148	5	1580	743.72	3.00	1706	106.66	5.13	0.143414188
45	116	4	45	45	4	45	148	5	1018	734.87	3.10	518	100.01	4.36	0.136092098
45	116	4	45	103	4	45	139	5	1463	732.72	2.87	1691	103.38	5.39	0.14109073
45	116	4	45	103	4	45	139	5	1156	720.27	3.49	1145	101.25	4.96	0.140572285
45	116	4	45	103	4	45	139	5	1301	715.03	3.29	1137	99.86	5.00	0.139658476
45	116	4	45	103	4	45	139	5	1246	746.08	4.25	1247	105.20	5.45	0.141003646
45	116	4	45	103	4	45	139	5	1674	738.38	3.33	901	102.91	5.04	0.139372681
45	116	4	45	148	5	45	45	4	1186	718.87	3.46	1475	98.83	5.96	0.137479656
45	116	4	45	148	5	45	45	4	1161	731.44	2.65	1260	105.17	6.01	0.143784863
45	116	4	45	148	5	45	45	4	1606	701.91	2.84	2074	96.61	5.02	0.137638729
45	116	4	45	148	5	45	45	4	1693	748.98	3.20	558	105.97	5.29	0.141485754
45	126	4	45	103	4	45	36	5	1747	730.90	3.96	1120	100.95	5.44	0.138117390
45	126	4	45	103	4	45	36	5	949	718.34	2.76	731	99.58	5.04	0.138625164
45	126	4	45	103	4	45	36	5	1449	723.95	2.99	1421	101.41	5.49	0.140078735
45	126	4	45	103	4	45	36	5	1758	732.59	2.86	1026	105.39	5.67	0.143859458
45	126	4	45	103	4	45	36	5	1361	696.04	2.58	1050	99.28	6.19	0.142635481
45	126	4	45	103	4	45	36	5	1382	739.60	2.60	1498	103.82	4.94	0.140373175
45	126	4	45	103	4	45	36	5	2483	713.14	3.04	399	99.57	5.34	0.139621954
45	126	4	45	36	5	45	103	4	1008	733.32	3.69	228	101.44	5.22	0.138329788
45	126	4	45	36	5	45	103	4	517	744.77	2.90	2487	103.83	5.23	0.139412168
45	126	4	45	36	5	45	103	4	1900	733.64	3.38	1163	105.24	4.92	0.143449103
45	126	4	45	36	5	45	103	4	987	774.55	3.37	946	109.88	5.15	0.141863017
45	126	4	45	36	5	45	103	4	1629	710.74	2.77	257	98.80	4.18	0.139010046
45	126	4	45	36	5	45	103	4	2166	721.57	2.63	1230	103.86	6.14	0.143936139
45	126	4	45	36	5	45	103	4	1632	709.47	2.77	696	98.94	4.60	0.139456214

Table S4. Continued,

C) Mixed-ploidy fertilizations seeds

Pollen recipient			Pollen donor 1			Pollen donor 2			Standard			Embryo			Embryo:Standard ratio
Population	Individual	Ploidy [*x*]	Population	Individual	Ploidy [*x*]	Population	Individual	Ploidy [*x*]	Count	Mean	CV	Count	Mean	CV	
45	126	4	45	36	5	45	103	4	1216	715.90	2.82	1358	98.36	4.85	0.137393491
45	143	4	45	45	4	45	133	5	542	730.09	3.30	1096	100.63	5.03	0.137832322
45	143	4	45	45	4	45	133	5	1029	763.28	3.00	1600	108.63	4.66	0.142319987
45	143	4	45	45	4	45	133	5	1062	751.13	2.48	1586	107.64	5.41	0.143304089
45	143	4	45	45	4	45	133	5	1237	730.88	3.00	1862	103.41	4.77	0.141486975
45	143	4	45	43	4	45	121	5	1260	744.85	3.01	1583	104.05	4.68	0.139692556
45	143	4	45	43	4	45	121	5	1676	704.45	3.16	763	91.57	6.45	0.129987934
45	143	4	45	43	4	45	121	5	1221	724.45	2.60	2110	100.82	5.73	0.139167644
45	143	4	45	43	4	45	121	5	1203	762.45	3.80	1428	107.07	5.63	0.140428881
45	143	4	45	45	4	45	57	5	1259	716.34	2.82	889	101.66	5.02	0.141915850
45	143	4	45	45	4	45	57	5	1118	720.82	2.76	1949	105.26	5.94	0.146028135
45	143	4	45	45	4	45	57	5	1062	737.61	2.88	1568	106.49	5.25	0.144371687
45	143	4	45	45	4	45	57	5	1378	733.96	3.30	1508	105.82	5.67	0.144176794
45	143	4	45	133	5	45	45	4	1333	746.88	3.02	1160	103.52	4.37	0.138603256
45	143	4	45	133	5	45	45	4	1558	732.67	4.00	2171	103.91	5.34	0.141823741
45	143	4	45	133	5	45	45	4	746	753.34	3.20	1762	124.87	5.04	0.165755170
45	143	4	45	133	5	45	45	4	976	734.27	3.84	1562	103.37	4.49	0.140779277
45	143	4	45	121	5	45	43	4	2228	733.12	3.66	775	103.30	5.07	0.140904627
45	143	4	45	121	5	45	43	4	1432	741.46	3.48	1681	104.09	4.45	0.140385186
45	143	4	45	121	5	45	43	4	1316	742.08	3.19	1270	103.82	4.56	0.139904053
45	143	4	45	121	5	45	43	4	1633	742.87	3.15	1689	104.62	4.69	0.140832178
45	143	4	45	121	5	45	43	4	1509	708.82	2.88	1256	98.64	4.69	0.139160859
45	146	4	45	127	4	45	41	5	1480	732.51	2.68	1541	103.78	4.44	0.141677247
45	146	4	45	127	4	45	41	5	879	754.62	2.91	2312	106.41	4.91	0.141011370
45	146	4	45	127	4	45	41	5	1802	733.66	2.68	1399	121.65	4.48	0.165812502
45	146	4	45	127	4	45	41	5	1416	742.61	3.46	2150	104.11	4.36	0.140194719
45	146	4	45	104	4	45	111	5	2649	725.52	3.07	634	99.49	5.48	0.137129231
45	146	4	45	104	4	45	111	5	1357	733.81	3.71	953	99.94	5.05	0.136193293
45	146	4	45	104	4	45	111	5	1282	733.79	3.32	1734	103.75	5.10	0.141389226
45	146	4	45	31	4	45	36	5	1180	737.32	3.10	1340	101.39	4.38	0.137511528
45	146	4	45	31	4	45	36	5	1106	733.64	3.28	2249	102.97	5.19	0.140354942
45	146	4	45	31	4	45	36	5	1478	739.56	2.29	1281	103.81	4.94	0.140367245
45	146	4	45	31	4	45	36	5	861	729.09	3.49	1905	102.98	5.03	0.141244565
45	146	4	45	111	5	45	104	4	1074	713.94	3.47	862	98.24	5.07	0.137602600
45	146	4	45	111	5	45	104	4	1115	726.05	2.90	1584	101.17	5.26	0.139343020
45	146	4	45	111	5	45	104	4	2176	748.19	3.78	411	102.05	4.41	0.136395835
45	146	4	45	111	5	45	104	4	908	764.60	3.59	1677	111.04	6.59	0.145226262
45	146	4	45	36	5	45	31	4	1113	692.78	2.99	1110	120.40	4.00	0.173792546

Table S4. Continued,

C) Mixed-ploidy fertilizations seeds

Pollen recipient			Pollen donor 1			Pollen donor 2			Standard			Embryo			Embryo:Standard ratio
Population	Individual	Ploidy [*x*]	Population	Individual	Ploidy [*x*]	Population	Individual	Ploidy [*x*]	Count	Mean	CV	Count	Mean	CV	
45	146	4	45	36	5	45	31	4	1274	713.18	3.37	1857	120.87	4.59	0.169480356
45	146	4	45	36	5	45	31	4	691	713.59	2.59	1109	115.84	5.13	0.162334113
45	146	4	45	36	5	45	31	4	1395	751.54	3.51	1550	125.43	4.65	0.166897304
64	14	4	64	20	4	68	117	5	491	694.48	3.23	761	126.40	5.01	0.182006681
64	14	4	64	20	4	68	117	5	571	688.97	3.24	893	95.15	5.25	0.138104707
64	14	4	64	20	4	68	117	5	585	730.05	3.21	810	133.56	5.72	0.182946374
64	14	4	64	20	4	68	117	5	615	720.30	3.13	822	98.90	4.42	0.137303901
64	14	4	64	20	4	68	117	5	527	714.12	3.27	1654	97.54	5.41	0.136587688
64	14	4	64	20	4	68	117	5	866	697.67	3.00	2157	92.93	4.87	0.133200510
64	14	4	64	43	4	68	37	5	756	709.06	5.52	879	100.10	6.42	0.141172820
64	14	4	64	43	4	68	37	5	562	723.08	6.90	1703	103.78	7.34	0.143524921
64	14	4	64	18	4	68	30	5	907	699.95	7.25	1300	97.98	8.07	0.139981427
64	14	4	64	18	4	68	30	5	976	716.10	3.24	1205	95.83	5.81	0.133822092
64	14	4	64	18	4	68	30	5	370	713.65	3.74	678	101.41	5.51	0.142100469
64	14	4	64	18	4	68	30	5	356	716.78	3.40	1343	98.69	6.08	0.137685203
64	14	4	68	37	5	64	43	4	848	709.10	3.39	1282	98.35	5.70	0.138696940
64	14	4	68	37	5	64	43	4	581	692.69	3.69	704	95.95	5.06	0.138517952
64	14	4	68	37	5	64	43	4	573	709.13	3.41	1067	99.46	4.98	0.140256370
64	14	4	68	37	5	64	43	4	662	687.59	3.38	979	95.94	5.36	0.139530825
64	14	4	68	37	5	64	43	4	287	714.42	3.35	857	97.99	5.21	0.137160214
64	14	4	68	37	5	64	43	4	362	712.49	3.43	1410	97.43	5.25	0.136745779
64	14	4	68	37	5	64	43	4	460	692.28	4.18	845	98.05	5.18	0.141633443
64	14	4	68	37	5	64	43	4	785	725.54	4.22	1272	103.48	6.13	0.142624804
64	14	4	68	30	5	64	18	4	658	704.78	4.33	682	98.95	4.91	0.140398422
64	14	4	68	30	5	64	18	4	435	713.50	4.72	564	96.66	6.30	0.135473020
64	14	4	68	30	5	64	18	4	707	715.63	4.28	1000	100.54	5.68	0.140491595
64	14	4	68	30	5	64	18	4	661	692.13	3.49	1055	95.43	4.85	0.137878722
64	14	4	68	30	5	64	18	4	615	727.02	3.93	1102	102.53	5.79	0.141027757
64	14	4	68	30	5	64	18	4	511	696.07	4.92	1011	99.65	5.12	0.143160889
64	14	4	68	30	5	64	18	4	559	695.10	4.78	932	97.43	5.85	0.140166882
64	14	4	68	30	5	64	18	4	591	695.99	4.23	1363	97.17	6.09	0.139614075
64	14	4	64	49	4	68	34	5	491	688.05	5.81	1006	98.37	7.44	0.142969261
64	17	4	64	49	4	68	34	5	529	707.84	3.89	1889	100.89	6.78	0.142532211
64	17	4	64	49	4	68	34	5	730	725.70	4.65	1813	105.10	6.11	0.144825686
64	17	4	64	49	4	68	34	5	539	724.17	3.45	746	100.81	6.47	0.139207645
64	17	4	64	49	4	68	34	5	636	704.27	4.56	1083	100.08	5.43	0.142104591
64	17	4	64	12	4	68	29	5	523	706.50	5.27	1418	99.20	6.47	0.140410474
64	17	4	64	12	4	68	29	5	810	693.69	3.50	1781	98.09	6.09	0.141403220

Table S4. Continued,

C) Mixed-ploidy fertilizations seeds

Pollen recipient			Pollen donor 1			Pollen donor 2			Standard			Embryo			Embryo:Standard ratio
Population	Individual	Ploidy [*x*]	Population	Individual	Ploidy [*x*]	Population	Individual	Ploidy [*x*]	Count	Mean	CV	Count	Mean	CV	
64	17	4	64	12	4	68	29	5	315	695.13	4.55	1029	97.11	5.55	0.139700488
64	17	4	64	55	4	68	7	5	505	723.95	3.57	606	103.18	6.90	0.142523655
64	17	4	68	34	5	64	49	4	551	709.77	3.40	1743	99.19	6.15	0.139749496
64	17	4	68	34	5	64	49	4	433	696.32	5.09	1737	95.16	6.65	0.136661305
64	17	4	68	34	5	64	49	4	475	694.87	4.30	1413	96.35	7.37	0.138659030
64	17	4	64	21	4	68	68	5	285	722.98	4.66	1025	101.67	5.83	0.140626297
64	20	4	68	32	5	64	21	4	286	727.80	4.72	1237	104.26	5.89	0.143253641
64	20	4	68	32	5	64	21	4	400	726.65	4.34	1253	101.84	6.32	0.140150003
64	20	4	68	32	5	64	21	4	568	692.82	3.62	1760	101.90	6.98	0.147080050
64	20	4	68	32	5	64	21	4	507	708.52	3.84	666	101.77	5.39	0.143637441
64	27	4	64	12	4	68	33	5	652	708.75	2.84	1271	97.47	5.48	0.137523810
64	27	4	64	12	4	68	33	5	526	700.88	3.51	1063	111.51	4.65	0.159099989
64	27	4	64	12	4	68	33	5	305	703.06	3.34	748	96.78	6.04	0.137655392
64	27	4	64	12	4	68	33	5	575	695.26	3.42	1226	94.66	6.31	0.136150505
64	27	4	64	12	4	68	33	5	557	718.63	3.52	743	123.53	4.97	0.171896525
64	27	4	64	12	4	68	33	5	433	705.74	3.39	1014	98.18	5.88	0.139116388
64	27	4	64	12	4	68	33	5	378	703.84	3.13	1164	96.54	6.57	0.137161855
64	27	4	64	12	4	68	33	5	283	715.39	3.05	495	98.37	3.84	0.137505417
64	36	4	64	12	4	68	35	5	316	695.03	2.31	1600	95.23	5.64	0.137015668
64	36	4	64	12	4	68	35	5	262	703.68	3.31	1538	97.04	6.75	0.137903593
64	36	4	64	12	4	68	35	5	201	719.49	3.57	915	98.47	5.31	0.136860832
64	44	4	64	22	4	68	27	5	1000	715.62	3.38	1728	99.62	6.60	0.139207960
64	44	4	68	27	5	64	22	4	875	708.01	3.05	791	101.89	5.34	0.143910397
64	44	4	68	27	5	64	22	4	944	711.95	2.99	2451	98.12	5.88	0.137818667
64	48	4	68	29	5	64	43	4	162	696.64	3.96	658	98.48	5.31	0.141364263
64	48	4	68	29	5	64	43	4	799	696.13	3.13	846	96.76	5.43	0.138997026
64	48	4	68	29	5	64	43	4	470	708.40	3.23	1358	98.62	5.74	0.139215133
64	48	4	68	29	5	64	43	4	524	714.52	3.13	1044	98.28	6.41	0.137546885
64	48	4	68	29	5	64	43	4	604	698.46	4.53	2022	95.35	6.23	0.136514618
64	48	4	68	29	5	64	12	4	654	714.14	3.10	781	95.93	4.92	0.134329403
64	14	4	64	20	4	68	71	7	515	687.73	3.06	340	109.10	6.60	0.158637838
64	14	4	64	20	4	68	71	7	201	698.02	3.56	155	111.42	6.76	0.159622933
64	14	4	68	71	7	64	20	4	320	697.42	2.90	410	96.20	5.31	0.137936968
64	14	4	68	71	7	64	20	4	558	704.30	2.58	410	132.86	4.30	0.188641204
64	14	4	68	71	7	64	20	4	461	687.95	3.15	623	93.67	5.23	0.136158151
64	14	4	68	71	7	64	20	4	617	695.21	3.27	976	96.13	6.89	0.138274766
64	14	4	68	71	7	64	20	4	839	709.37	2.92	667	97.52	5.95	0.137474097
64	14	4	68	71	7	64	20	4	448	699.09	3.12	1098	96.63	5.54	0.138222546

Table S4. Continued,

C) Mixed-ploidy fertilizations seeds

Pollen recipient			Pollen donor 1			Pollen donor 2			Standard			Embryo			Embryo:Standard ratio
Population	Individual	Ploidy [*x*]	Population	Individual	Ploidy [*x*]	Population	Individual	Ploidy [*x*]	Count	Mean	CV	Count	Mean	CV	
64	14	4	68	71	7	64	20	4	1816	693.90	3.15	367	130.40	7.40	0.187923332
64	14	4	68	71	7	64	20	4	2155	713.35	2.53	647	136.41	4.55	0.191224504
64	14	4	68	71	7	64	20	4	551	692.19	3.09	1466	95.69	5.42	0.138242390
64	14	4	68	71	7	64	20	4	732	702.96	3.42	705	97.76	6.30	0.139069079
64	14	4	68	71	7	64	20	4	1114	700.30	2.74	188	163.36	5.64	0.233271455
64	17	4	68	6	7	64	55	4	262	694.63	3.16	781	148.01	5.64	0.213077466
64	17	4	68	6	7	64	55	4	351	706.63	2.84	1299	96.74	5.69	0.136903330
64	17	4	68	6	7	64	55	4	192	702.11	3.17	1548	100.09	6.03	0.142556010
64	17	4	68	6	7	64	55	4	702	699.92	2.89	1872	99.08	6.10	0.141559035
64	17	4	68	6	7	64	55	4	363	696.86	3.20	1055	96.42	5.84	0.138363516
64	17	4	68	6	7	64	55	4	526	705.40	3.05	1548	95.75	6.50	0.135738588
64	20	4	64	18	4	68	16	7	765	689.09	3.06	903	93.78	5.61	0.136092528
64	20	4	64	18	4	68	16	7	454	697.54	3.48	228	94.60	5.82	0.135619463
64	20	4	64	18	4	68	16	7	339	697.56	3.19	2205	96.70	5.99	0.138626068
64	20	4	68	16	7	64	18	4	557	693.32	3.60	1209	94.32	7.00	0.136041078
64	20	4	68	16	7	64	18	4	444	696.38	2.76	2264	94.53	5.63	0.135744852
64	27	4	64	32	4	68	16	7	452	698.65	3.32	897	96.02	5.17	0.137436485
64	27	4	64	32	4	68	16	7	496	693.75	3.74	1109	97.85	6.16	0.141045045
64	27	4	64	32	4	68	16	7	1667	706.71	5.02	681	94.37	5.18	0.133534264
64	27	4	64	32	4	68	16	7	801	686.43	5.09	952	92.98	6.28	0.135454453
64	27	4	64	32	4	68	16	7	479	704.45	3.28	478	97.38	5.20	0.138235503
64	27	4	68	100	7	68	16	7	517	687.17	3.89	1160	100.45	5.07	0.146179257
64	27	4	68	100	7	68	16	7	231	691.07	3.73	1522	97.57	6.30	0.141186855
64	27	4	64	32	4	68	16	7	866	697.60	3.50	1063	94.75	5.92	0.135822821
64	27	4	64	32	4	68	16	7	179	704.26	3.98	300	136.00	4.89	0.193110499
64	27	4	64	32	4	68	16	7	1106	702.63	3.37	926	97.13	5.34	0.138237764
64	27	4	64	32	4	68	16	7	1038	683.84	3.78	844	97.55	5.13	0.142650328
64	27	4	64	32	4	68	16	7	749	681.56	3.41	1456	96.64	5.43	0.141792359
64	27	4	64	32	4	68	16	7	589	700.50	3.30	538	95.48	7.04	0.136302641
64	27	4	64	32	4	68	16	7	1024	706.39	3.74	2504	96.26	7.10	0.136270332
64	27	4	64	32	4	68	16	7	476	725.27	4.07	1228	98.04	6.14	0.135177244
64	27	4	64	32	4	68	16	7	852	715.40	4.05	861	98.77	5.63	0.138062622
64	27	4	68	120	7	64	12	4	1336	702.43	3.87	2058	98.56	6.32	0.140312914
64	27	4	68	120	7	64	12	4	1338	702.98	3.81	790	95.58	5.24	0.135964039
64	27	4	68	120	7	64	12	4	958	682.00	3.76	2502	96.32	5.84	0.141231672
64	27	4	68	120	7	64	12	4	529	702.81	3.37	833	95.48	5.23	0.135854641
64	27	4	68	120	7	64	12	4	548	687.00	3.79	928	131.87	5.71	0.191950509
64	27	4	68	120	7	64	12	4	810	702.54	3.29	1003	96.79	6.34	0.137771515

Table S4. Continued,

C) Mixed-ploidy fertilizations seeds

Pollen recipient			Pollen donor 1			Pollen donor 2			Standard			Embryo			Embryo:Standard ratio
Population	Individual	Ploidy [*x*]	Population	Individual	Ploidy [*x*]	Population	Individual	Ploidy [*x*]	Count	Mean	CV	Count	Mean	CV	
64	27	4	68	120	7	64	12	4	267	696.15	4.55	1130	95.91	6.77	0.137772032
64	27	4	68	120	7	64	12	4	1575	695.71	3.35	1863	96.96	6.06	0.139368415
64	44	4	64	34	4	68	55	7	408	697.70	4.19	1007	95.74	5.51	0.137222302
64	44	4	64	34	4	68	55	7	862	691.32	4.16	1354	96.27	7.29	0.139255338
64	44	4	64	34	4	68	55	7	798	711.05	3.83	1023	98.31	5.88	0.138260319
64	44	4	64	34	4	68	55	7	602	691.54	3.65	1011	94.28	5.42	0.136333401
64	44	4	64	34	4	68	55	7	794	703.91	3.83	836	94.17	5.32	0.133781307
64	44	4	64	34	4	68	55	7	260	688.14	4.77	359	91.69	6.04	0.133243235
64	44	4	64	34	4	68	55	7	598	685.39	3.77	1011	95.64	5.94	0.139540991
64	44	4	68	55	7	64	34	4	1142	698.53	3.22	2367	99.43	6.56	0.142341775
64	44	4	68	55	7	64	34	4	932	697.70	2.92	1076	96.85	6.44	0.138813244
64	48	4	64	12	4	68	46	7	575	678.30	7.75	783	92.92	6.26	0.136989533
64	48	4	64	27	4	68	74	7	615	695.60	4.06	702	127.87	5.50	0.183826912
64	48	4	64	27	4	68	74	7	650	697.59	2.87	771	121.11	5.11	0.173612007
64	48	4	64	27	4	68	74	7	912	689.08	3.48	1563	93.55	5.67	0.135760724
64	48	4	64	27	4	68	74	7	668	693.72	3.18	914	123.83	5.23	0.178501413
64	48	4	64	27	4	68	74	7	522	700.70	3.24	1236	94.84	5.29	0.135350364
64	48	4	64	27	4	68	74	7	646	685.73	3.14	500	180.11	5.81	0.262654398
64	48	4	68	46	7	64	12	4	440	701.54	3.35	814	144.12	5.12	0.205433760
64	48	4	68	46	7	64	12	4	605	713.13	3.37	463	97.59	5.59	0.136847419
64	48	4	68	46	7	64	12	4	692	707.50	3.50	1363	98.68	4.79	0.139477032
64	48	4	68	74	7	64	27	4	437	695.22	3.53	1529	97.04	5.85	0.139581715
64	48	4	68	74	7	64	27	4	717	695.37	3.66	1045	99.60	4.93	0.143233099
121	16	4	119	48	5	121	31	4	356	709.50	3.67	881	108.68	5.45	0.153178295
121	18	4	121	33	4	119	13	5	293	734.14	3.23	713	103.22	5.10	0.140599886
121	18	4	121	33	4	119	13	5	377	712.93	3.28	1189	101.45	5.76	0.142300086
121	18	4	121	33	4	119	13	5	639	703.36	2.77	2174	99.34	5.68	0.141236351
121	18	4	119	19	5	121	29	4	767	714.20	3.15	1227	101.62	5.68	0.142285074
121	18	4	119	19	5	121	29	4	106	708.59	4.01	855	98.42	5.62	0.138895553
121	18	4	119	19	5	121	29	4	407	749.75	3.56	981	103.72	4.95	0.138339446
121	18	4	119	19	5	121	29	4	1004	722.79	2.63	633	99.19	4.85	0.137232114
121	18	4	119	19	5	121	29	4	433	721.06	2.84	892	102.81	4.51	0.142581755
121	18	4	119	19	5	121	29	4	362	697.52	5.81	1505	103.20	6.87	0.147952747
121	18	4	119	19	5	121	29	4	546	779.88	3.14	1852	112.66	6.13	0.144458122
121	18	4	119	19	5	121	29	4	260	705.60	3.18	831	99.64	5.41	0.141213152
121	18	4	119	19	5	121	29	4	263	706.63	3.87	704	99.47	5.87	0.140766738
121	18	4	119	12	5	121	31	4	290	695.38	4.52	1769	95.13	7.05	0.136802899
121	18	4	119	12	5	121	31	4	496	706.13	3.19	1195	100.10	6.82	0.141758600

Table S4. Continued,

C) Mixed-ploidy fertilizations seeds

Pollen recipient			Pollen donor 1			Pollen donor 2			Standard			Embryo			Embryo:Standard ratio
Population	Individual	Ploidy [*x*]	Population	Individual	Ploidy [*x*]	Population	Individual	Ploidy [*x*]	Count	Mean	CV	Count	Mean	CV	
121	18	4	119	12	5	121	31	4	331	694.77	3.89	570	105.72	5.05	0.152165465
121	18	4	119	12	5	121	31	4	534	742.66	3.64	865	105.00	5.19	0.141383675
121	18	4	119	12	5	121	31	4	393	703.74	3.33	833	98.00	4.91	0.139255975
121	18	4	119	12	5	121	31	4	432	740.24	3.15	837	102.97	5.49	0.139103534
121	18	4	119	12	5	121	31	4	777	732.17	3.60	837	106.64	5.57	0.145649234
121	18	4	119	12	5	121	31	4	396	704.88	3.36	1893	99.29	6.27	0.140860856
121	18	4	119	12	5	121	31	4	332	693.79	3.32	904	98.34	5.40	0.141743179
121	18	4	119	13	5	121	33	4	321	716.05	3.11	1567	103.78	5.07	0.144934013
121	18	4	119	13	5	121	33	4	517	721.56	3.31	1685	102.71	5.83	0.142344365
121	18	4	119	13	5	121	33	4	313	721.03	3.61	1144	103.42	5.27	0.143433699
121	20	4	121	10	4	119	24	5	466	712.33	3.82	706	103.34	4.97	0.145073210
121	20	4	121	10	4	119	24	5	468	713.19	3.45	635	103.27	5.15	0.144800123
121	20	4	121	10	4	119	24	5	179	716.40	3.44	1026	104.12	6.03	0.145337800
121	20	4	121	10	4	119	24	5	409	711.85	3.74	820	100.84	4.82	0.141659057
121	20	4	121	10	4	119	24	5	461	707.23	3.49	1048	102.73	6.27	0.145256847
121	20	4	121	10	4	119	24	5	479	707.31	3.05	868	119.82	5.15	0.169402384
121	20	4	121	10	4	119	24	5	683	705.63	3.00	870	99.57	4.96	0.141107946
121	20	4	121	10	4	119	24	5	271	680.34	4.96	333	124.45	8.14	0.182923244
121	20	4	121	31	4	119	60	5	284	731.55	3.00	1002	129.13	4.80	0.176515618
121	20	4	121	31	4	119	60	5	246	709.76	3.17	1756	98.98	5.55	0.139455591
121	20	4	121	31	4	119	60	5	455	734.96	3.47	1272	116.82	5.64	0.158947426
121	20	4	121	31	4	119	60	5	467	727.35	3.53	899	105.81	5.86	0.145473293
121	20	4	121	31	4	119	60	5	345	706.82	3.72	1039	100.85	5.68	0.142681305
121	20	4	119	3	5	121	3	4	546	699.37	3.71	1302	100.73	5.65	0.144029627
121	20	4	119	60	5	121	31	4	650	711.41	5.76	745	123.25	6.74	0.173247494
121	20	4	119	60	5	121	31	4	379	762.95	3.18	436	106.86	5.29	0.140061603
121	20	4	119	60	5	121	31	4	306	718.79	4.11	512	101.09	5.59	0.140639130
121	22	4	121	52	4	119	25	5	464	730.59	3.07	2825	101.97	5.65	0.139572127
121	22	4	121	52	4	119	25	5	359	711.36	3.43	690	102.09	6.49	0.143513833
121	22	4	121	6	4	119	28	5	334	741.77	3.58	1759	110.62	5.95	0.149129784
121	22	4	119	28	5	121	6	4	691	733.55	3.03	1209	108.07	4.90	0.147324654
121	26	4	121	11	4	119	44	5	543	744.40	3.24	1932	106.66	4.94	0.143283181
121	26	4	121	11	4	119	44	5	191	731.74	2.95	859	101.12	6.31	0.138191161
121	26	4	121	11	4	119	44	5	357	720.17	3.25	2326	101.06	6.45	0.140327978
121	26	4	121	10	4	119	28	5	353	734.67	3.39	487	102.63	4.69	0.139695373
121	26	4	121	10	4	119	28	5	288	731.36	3.55	1254	103.05	5.61	0.140901881
121	26	4	121	10	4	119	28	5	500	723.77	3.65	846	125.92	5.30	0.173977921
121	26	4	121	10	4	119	28	5	1141	719.71	3.65	2127	99.07	6.10	0.137652666

Table S4. Continued,

C) Mixed-ploidy fertilizations seeds

Pollen recipient			Pollen donor 1			Pollen donor 2			Standard			Embryo			Embryo:Standard ratio
Population	Individual	Ploidy [*x*]	Population	Individual	Ploidy [*x*]	Population	Individual	Ploidy [*x*]	Count	Mean	CV	Count	Mean	CV	
121	26	4	121	10	4	119	28	5	389	732.65	3.46	949	103.89	4.84	0.141800314
121	26	4	121	10	4	119	28	5	541	707.98	3.82	883	136.20	5.83	0.192378316
121	26	4	121	2	4	119	47	5	297	715.13	3.19	638	119.30	4.01	0.166822815
121	26	4	121	2	4	119	47	5	1326	714.82	2.68	1118	101.21	3.83	0.141588092
121	26	4	121	2	4	119	47	5	520	721.63	3.00	647	97.91	4.45	0.135678949
121	26	4	121	2	4	119	47	5	457	716.98	2.83	863	98.54	4.76	0.137437585
121	26	4	121	2	4	119	47	5	399	717.17	3.12	1250	97.87	4.57	0.136466946
121	26	4	119	44	5	121	11	4	516	709.52	2.78	960	118.08	3.86	0.166422370
121	26	4	119	44	5	121	11	4	301	699.40	2.78	1072	96.83	4.22	0.138447240
121	26	4	119	44	5	121	11	4	966	705.97	2.89	895	121.54	3.61	0.172160290
121	26	4	119	44	5	121	11	4	410	705.84	2.82	753	95.17	3.32	0.134832257
121	26	4	119	44	5	121	11	4	650	724.59	3.00	575	100.59	4.87	0.138823335
121	26	4	119	47	5	121	2	4	949	733.20	3.12	685	102.38	4.06	0.139634479
121	26	4	119	47	5	121	2	4	566	711.78	2.99	467	101.25	3.62	0.142249010
121	27	4	121	9	4	119	1	5	479	722.21	2.89	609	98.50	3.58	0.136386923
121	27	4	121	9	4	119	1	5	727	726.12	3.17	846	103.60	4.31	0.142676142
121	27	4	121	9	4	119	1	5	536	725.01	3.39	814	101.68	4.32	0.140246341
121	27	4	121	9	4	119	1	5	658	687.10	3.49	605	93.49	4.37	0.136064619
121	27	4	121	9	4	119	1	5	956	726.00	3.33	1025	102.56	4.09	0.141267218
121	27	4	121	2	4	119	48	5	803	722.15	3.18	682	101.10	4.21	0.139998615
121	27	4	121	2	4	119	48	5	379	724.46	3.83	589	98.57	4.38	0.136059962
121	27	4	121	2	4	119	48	5	1199	705.57	3.47	1774	98.15	4.72	0.139107388
121	27	4	121	2	4	119	48	5	933	695.55	3.58	1849	97.86	4.69	0.140694414
121	27	4	121	6	4	119	8	5	1048	708.37	3.58	2961	97.40	4.49	0.137498765
121	27	4	121	6	4	119	8	5	1151	708.15	4.23	2877	100.29	4.57	0.141622538
121	27	4	121	6	4	119	8	5	3483	708.52	4.51	2020	99.77	4.60	0.140814656
121	27	4	121	6	4	119	8	5	1558	727.96	3.97	2714	103.09	4.76	0.141614924
121	27	4	119	1	5	121	9	4	1010	713.41	3.95	1697	101.52	4.93	0.142302463
121	27	4	119	1	5	121	9	4	543	707.11	4.35	1068	99.27	4.79	0.140388341
121	27	4	119	1	5	121	9	4	1257	721.01	3.99	2043	99.37	4.74	0.137820557
121	27	4	119	1	5	121	9	4	785	706.67	3.66	1673	98.75	4.34	0.139739907
121	27	4	119	8	5	121	6	4	329	726.95	3.27	1260	98.11	4.19	0.134961139
121	27	4	119	8	5	121	6	4	510	708.20	4.02	1559	94.70	3.93	0.133719288
121	27	4	119	8	5	121	6	4	359	682.47	3.36	1126	92.67	4.69	0.135786188
121	30	4	121	12	4	119	19	5	995	723.46	3.64	2721	98.69	4.30	0.136413900
121	30	4	121	12	4	119	19	5	1009	712.65	3.61	2763	99.90	4.57	0.140181015
121	30	4	121	13	4	119	31	5	485	714.91	3.30	2113	98.49	4.19	0.137765593
121	30	4	121	13	4	119	31	5	334	694.39	3.80	1858	93.21	5.01	0.134232924

Table S4. Continued,

C) Mixed-ploidy fertilizations seeds

Pollen recipient			Pollen donor 1			Pollen donor 2			Standard			Embryo			Embryo:Standard ratio
Population	Individual	Ploidy [*x*]	Population	Individual	Ploidy [*x*]	Population	Individual	Ploidy [*x*]	Count	Mean	CV	Count	Mean	CV	
121	30	4	119	31	5	121	52	4	1006	727.54	3.29	3228	102.39	4.62	0.140734530
121	30	4	119	31	5	121	52	4	922	713.16	2.98	2454	102.11	3.55	0.143179651
121	30	4	119	31	5	121	52	4	1008	700.13	3.41	3656	98.09	4.46	0.140102552
121	30	4	119	31	5	121	52	4	1071	712.90	3.87	3942	94.75	5.16	0.132907841
121	30	4	119	31	5	121	52	4	1014	724.32	3.47	4610	97.06	5.77	0.134001546
121	30	4	119	31	5	121	52	4	1504	700.41	3.82	2981	97.61	4.37	0.139361231
121	30	4	119	32	5	121	13	4	1507	701.50	3.65	4204	95.76	5.15	0.136507484
121	30	4	119	32	5	121	13	4	1827	698.62	3.61	3233	95.66	4.36	0.136927085
121	30	4	119	32	5	121	13	4	1798	694.79	3.81	2874	97.02	4.47	0.139639315
121	30	4	119	32	5	121	13	4	1579	710.42	2.76	3656	97.82	4.47	0.137693196
121	30	4	119	32	5	121	13	4	1876	703.94	3.50	3854	97.55	5.04	0.138577151
121	30	4	119	32	5	121	13	4	2064	708.15	3.69	3618	98.67	5.04	0.139334887
121	36	4	121	12	4	119	12	5	818	707.38	2.95	3528	98.73	4.71	0.139571376
121	36	4	121	5	4	119	1	5	1288	698.23	3.05	6072	94.26	5.34	0.134998496
121	36	4	121	5	4	119	1	5	824	701.96	2.85	3827	93.85	5.15	0.133697077
121	36	4	121	5	4	119	1	5	2192	699.47	3.43	3998	94.71	5.15	0.135402519
121	36	4	121	5	4	119	1	5	1370	694.95	3.41	2471	92.33	4.62	0.132858479
121	36	4	121	5	4	119	1	5	1311	701.71	3.37	3576	93.79	4.74	0.133659204
121	36	4	121	5	4	119	1	5	1680	705.69	3.47	3798	96.66	4.89	0.136972325
121	36	4	119	12	5	121	12	4	832	695.41	2.87	2200	95.95	3.95	0.137976158
121	36	4	119	12	5	121	12	4	1753	704.04	3.54	3577	96.32	4.68	0.136810408
121	36	4	119	12	5	121	12	4	981	701.89	3.05	3116	94.13	4.41	0.134109333
121	36	4	119	12	5	121	12	4	955	697.65	3.47	3068	95.08	4.35	0.136286103
121	36	4	119	1	5	121	5	4	1313	706.84	3.11	4130	96.37	4.92	0.136339200
121	36	4	119	1	5	121	5	4	649	706.67	3.17	3204	94.59	4.54	0.133853142
121	36	4	119	47	5	121	29	4	1116	698.50	3.45	1917	96.23	3.70	0.137766643
121	37	4	121	5	4	119	8	5	806	695.29	3.54	1987	97.66	4.00	0.140459377
121	37	4	121	13	4	119	13	5	802	694.19	3.22	2635	120.89	4.25	0.174145407
121	37	4	121	13	4	119	13	5	1534	701.96	3.79	3858	97.97	5.14	0.139566357
121	37	4	121	13	4	119	13	5	835	704.91	3.62	3607	97.96	5.01	0.138968095
121	37	4	121	13	4	119	13	5	527	712.09	2.81	1974	97.10	4.56	0.136359168
121	37	4	121	11	4	119	24	5	803	708.82	3.30	3722	97.02	5.19	0.136875370
121	37	4	121	11	4	119	24	5	1236	713.52	3.48	4323	96.25	4.95	0.134894607
121	37	4	121	11	4	119	24	5	1833	708.76	3.58	1901	98.44	4.23	0.138890457
121	37	4	121	11	4	119	24	5	887	692.35	4.07	1441	94.28	4.86	0.136173900
121	37	4	119	8	5	121	5	4	752	720.68	3.47	3051	98.47	4.90	0.136634845
121	37	4	119	8	5	121	5	4	399	692.67	3.43	3385	96.27	4.80	0.138983932
121	37	4	119	8	5	121	5	4	779	704.91	3.46	2973	97.96	4.70	0.138968095

Table S4. Continued,

C) Mixed-ploidy fertilizations seeds

Pollen recipient			Pollen donor 1			Pollen donor 2			Standard			Embryo			Embryo:Standard ratio
Population	Individual	Ploidy [*x*]	Population	Individual	Ploidy [*x*]	Population	Individual	Ploidy [*x*]	Count	Mean	CV	Count	Mean	CV	
121	37	4	119	8	5	121	5	4	415	705.94	3.21	3134	97.58	4.60	0.138227045
121	37	4	119	13	5	121	13	4	995	708.63	3.37	3551	97.57	4.57	0.137688215
121	37	4	119	13	5	121	13	4	925	714.14	3.13	4513	98.41	4.58	0.137802112
121	37	4	119	13	5	121	13	4	696	711.30	3.36	3245	98.27	4.35	0.138155490
121	37	4	119	13	5	121	13	4	448	682.23	4.11	2930	86.74	5.00	0.127141873
121	37	4	119	24	5	121	11	4	1543	704.27	3.71	6367	93.47	6.18	0.132718986
121	37	4	119	24	5	121	11	4	1275	693.02	3.71	4238	93.57	4.76	0.135017748
121	40	4	121	6	4	119	25	5	1548	692.10	3.25	4821	95.75	5.55	0.138347060
121	40	4	121	6	4	119	25	5	896	722.89	3.15	2691	100.26	5.18	0.138693301
121	40	4	121	3	4	119	66	5	1546	728.50	3.56	4555	97.78	5.52	0.134221002
121	40	4	121	3	4	119	66	5	1383	726.20	3.54	2853	100.55	4.27	0.138460479
121	40	4	121	3	4	119	66	5	1315	713.65	2.87	2543	99.11	4.55	0.138877601
121	40	4	121	3	4	119	66	5	742	702.02	3.09	3231	97.29	4.50	0.138585795
121	40	4	121	3	4	119	66	5	1084	710.48	3.44	1440	120.06	4.42	0.168984349
121	40	4	121	6	4	119	32	5	1850	703.36	3.99	3415	98.17	4.87	0.139572907
121	40	4	121	6	4	119	32	5	650	714.99	3.16	3400	100.49	4.91	0.140547420
121	40	4	121	6	4	119	32	5	661	723.70	3.08	3161	102.28	4.11	0.141329280
121	40	4	121	6	4	119	32	5	870	722.00	3.11	3168	100.40	4.19	0.139058172
121	40	4	121	6	4	119	32	5	610	709.56	3.36	3073	98.03	4.44	0.138156040
121	40	4	119	66	5	121	3	4	1104	697.16	3.28	3566	98.69	4.29	0.141560044
121	40	4	119	66	5	121	3	4	619	706.49	3.38	2463	99.64	4.79	0.141035259
121	40	4	119	66	5	121	3	4	1203	707.55	3.75	3633	99.39	4.80	0.140470638
121	40	4	119	66	5	121	3	4	814	697.03	3.29	2562	100.76	4.01	0.144556188
121	40	4	121	32	4	119	6	5	545	708.39	2.89	2933	98.64	4.61	0.139245331
121	40	4	121	32	4	119	6	5	675	713.02	2.99	2579	100.76	4.84	0.141314409
121	40	4	121	32	4	119	6	5	1802	700.49	3.53	2346	98.10	4.51	0.140044826
121	40	4	121	32	4	119	6	5	1050	712.88	3.03	2155	100.20	3.92	0.140556615
121	43	4	121	44	4	119	66	5	1300	700.82	3.06	2615	99.19	4.17	0.141534203
121	43	4	121	44	4	119	66	5	2174	700.84	2.69	3530	97.17	4.97	0.138647908
121	43	4	121	44	4	119	66	5	1370	712.37	3.35	3659	99.87	4.72	0.140194000
121	43	4	121	44	4	119	66	5	1462	709.72	3.15	3157	100.24	4.09	0.141238798
121	43	4	121	44	4	119	66	5	854	715.57	2.98	3280	99.63	4.84	0.139231661
121	43	4	121	31	4	119	31	5	1694	714.02	3.13	2607	100.01	4.45	0.140066105
121	43	4	121	31	4	119	31	5	669	700.43	2.97	3372	97.42	4.77	0.139085990
121	43	4	121	31	4	119	31	5	912	689.83	3.05	3133	97.97	4.65	0.142020498
121	43	4	121	31	4	119	31	5	851	695.73	3.42	3127	97.82	4.55	0.140600520
121	43	4	121	31	4	119	31	5	245	686.82	3.55	521	121.86	4.43	0.177426400
121	43	4	121	33	4	119	3	5	838	687.09	3.35	3918	95.94	4.80	0.139632363

Table S4. Continued,

C) Mixed-ploidy fertilizations seeds

Pollen recipient			Pollen donor 1			Pollen donor 2			Standard			Embryo			Embryo:Standard ratio
Population	Individual	Ploidy [*x*]	Population	Individual	Ploidy [*x*]	Population	Individual	Ploidy [*x*]	Count	Mean	CV	Count	Mean	CV	
121	43	4	121	33	4	119	3	5	1142	684.41	3.30	3983	100.27	5.03	0.146505749
121	43	4	121	33	4	119	3	5	1024	697.04	3.27	3711	96.80	5.07	0.138872948
121	43	4	121	33	4	119	3	5	534	705.09	3.05	2185	96.58	4.64	0.136975422
121	43	4	119	3	5	121	33	4	675	704.96	3.14	2511	97.59	4.79	0.138433386
121	43	4	119	3	5	121	33	4	1739	699.36	3.39	2945	98.99	4.04	0.141543697
121	43	4	119	3	5	121	33	4	607	711.89	2.72	1812	99.44	4.46	0.139684502
121	43	4	119	3	5	121	33	4	1004	723.22	3.14	3315	101.06	4.47	0.139736180
121	43	4	119	3	5	121	33	4	433	716.18	3.09	1926	101.22	4.76	0.141333184
121	18	4	121	52	4	119	53	7	955	715.66	3.70	1104	130.74	5.81	0.182684515
121	18	4	121	52	4	119	53	7	571	717.15	3.25	867	102.03	5.43	0.142271491
121	18	4	121	52	4	119	52	7	912	697.57	3.43	1178	99.13	5.60	0.142107602
121	18	4	119	53	7	121	52	4	1411	685.15	3.59	2093	99.53	6.55	0.145267460
121	18	4	119	53	7	121	52	4	1390	700.01	3.51	1786	96.90	6.39	0.138426594
121	18	4	119	53	7	121	52	4	801	695.39	3.26	1283	97.43	6.15	0.140108428
121	18	4	119	53	7	121	52	4	538	689.91	3.27	2568	93.28	7.05	0.135206041
121	18	4	119	53	7	121	52	4	992	705.87	3.62	1063	99.80	6.05	0.141385808
121	18	4	119	53	7	121	52	4	709	702.77	4.12	1094	97.22	6.82	0.138338290
121	18	4	119	53	7	121	52	4	691	699.79	3.71	649	94.76	6.37	0.135412052
121	18	4	119	53	7	121	52	4	807	697.71	4.04	475	95.91	5.64	0.137463989
121	18	4	119	53	7	121	52	4	552	697.41	3.98	1052	100.78	6.02	0.144506101
121	20	4	121	31	4	119	50	7	824	687.44	3.39	802	96.66	5.25	0.140608635
121	20	4	121	31	4	119	50	7	1004	693.05	3.49	2071	97.93	5.80	0.141302936
121	20	4	121	31	4	119	50	7	511	686.94	3.13	1646	94.31	5.90	0.137290011
121	20	4	121	31	4	119	50	7	607	712.53	3.53	817	97.25	5.42	0.136485481
121	20	4	121	31	4	119	50	7	776	698.85	3.09	1667	99.96	5.48	0.143034986
121	20	4	121	3	4	119	10	7	522	720.21	3.49	1246	98.45	6.27	0.136696241
121	20	4	121	3	4	119	10	7	1042	675.47	3.48	2255	92.48	5.65	0.136912076
121	20	4	121	3	4	119	10	7	378	706.90	3.52	1076	96.51	5.93	0.136525675
121	20	4	121	3	4	119	10	7	453	700.13	3.68	350	100.15	7.68	0.143044863
121	20	4	121	3	4	119	10	7	606	673.02	6.31	1054	92.24	6.39	0.137053877
121	20	4	119	50	7	121	31	4	645	690.46	3.70	2166	95.66	6.18	0.138545318
121	20	4	119	50	7	121	31	4	1008	698.76	3.26	1757	99.43	5.32	0.142294922
121	20	4	119	50	7	121	31	4	358	704.62	3.29	1026	97.52	4.98	0.138400840
121	20	4	119	50	7	121	31	4	1086	697.14	3.43	1657	100.11	6.22	0.143600998
121	20	4	119	10	7	121	3	4	515	698.53	3.15	692	95.56	4.66	0.136801569
121	20	4	119	10	7	121	3	4	281	696.24	4.84	737	95.70	4.94	0.137452603
121	20	4	119	10	7	121	3	4	470	694.18	3.26	1630	95.31	5.45	0.137298683
121	20	4	119	10	7	121	3	4	286	700.96	3.37	1510	96.53	5.05	0.137711139

Table S4. Continued,

C) Mixed-ploidy fertilizations seeds

Pollen recipient			Pollen donor 1			Pollen donor 2			Standard			Embryo			Embryo:Standard ratio
Population	Individual	Ploidy [*x*]	Population	Individual	Ploidy [*x*]	Population	Individual	Ploidy [*x*]	Count	Mean	CV	Count	Mean	CV	
121	22	4	121	3	4	119	14	7	291	677.73	3.52	831	90.01	5.05	0.132811001
121	22	4	121	3	4	119	14	7	519	710.59	3.28	834	95.55	4.98	0.134465726
121	22	4	121	3	4	119	14	7	700	701.25	3.28	888	94.00	5.93	0.134046346
121	22	4	121	3	4	119	14	7	459	686.03	3.66	1534	92.67	5.35	0.135081556
121	22	4	121	6	4	119	27	7	651	689.85	3.46	1654	94.56	6.97	0.137073277
121	22	4	121	6	4	119	27	7	624	688.87	3.26	1330	95.58	5.86	0.138748966
121	22	4	121	6	4	119	27	7	236	683.61	3.43	1270	96.50	4.45	0.141162359
121	22	4	121	6	4	119	27	7	741	700.10	4.11	936	92.53	6.70	0.132166833
121	22	4	121	6	4	119	27	7	250	718.74	3.82	442	95.20	5.14	0.132454017
121	22	4	121	52	4	119	10	7	636	704.28	3.30	945	98.41	5.07	0.139731357
121	22	4	121	52	4	119	10	7	479	706.58	3.60	1461	96.98	6.05	0.137252682
121	22	4	121	52	4	119	10	7	457	703.00	3.56	683	120.25	5.26	0.171052632
121	22	4	119	14	7	121	3	4	278	696.09	3.30	794	95.53	5.47	0.137238001
121	22	4	119	14	7	121	3	4	254	697.35	2.74	353	86.94	6.51	0.124671972
121	22	4	119	14	7	121	3	4	629	705.23	2.89	2029	95.19	5.64	0.134977241
121	22	4	119	27	7	121	6	4	931	709.07	3.34	1114	95.80	5.65	0.135106548
121	22	4	119	27	7	121	6	4	646	711.35	3.45	1135	96.00	7.32	0.134954664
121	22	4	119	27	7	121	6	4	376	692.70	3.45	1140	92.76	5.63	0.133910784
121	22	4	119	27	7	121	6	4	161	702.24	3.52	480	91.86	6.25	0.130809979
121	26	4	121	2	4	119	9	7	1144	731.91	3.80	1524	100.92	5.14	0.137885806
121	26	4	121	2	4	119	9	7	284	721.04	3.89	1216	98.48	5.32	0.136580495
121	26	4	121	2	4	119	9	7	481	718.63	3.08	617	96.01	6.33	0.133601436
121	26	4	121	2	4	119	9	7	564	698.13	3.02	1058	95.13	5.96	0.136264020
121	26	4	121	2	4	119	9	7	1203	684.77	3.02	1819	93.20	5.33	0.136104093
121	26	4	121	2	4	119	9	7	831	701.04	2.95	581	93.17	4.14	0.132902545
121	26	4	121	2	4	119	9	7	507	706.09	3.12	372	94.78	6.18	0.134232180
121	26	4	121	2	4	119	9	7	1051	704.73	2.97	600	98.24	4.30	0.139400905
121	26	4	119	11	7	121	10	4	254	699.99	3.24	998	98.01	5.96	0.140016286
121	26	4	119	11	7	121	10	4	397	712.16	2.84	1004	98.60	4.50	0.138452033
121	26	4	119	9	7	121	2	4	415	687.94	2.81	1296	92.76	5.26	0.134837340
121	26	4	119	9	7	121	2	4	677	701.25	2.72	674	92.62	4.54	0.132078431
121	26	4	119	9	7	121	2	4	742	700.46	3.02	504	94.60	5.82	0.135054107
121	26	4	119	9	7	121	2	4	1018	687.77	2.96	1173	91.24	5.44	0.132660628
121	26	4	119	9	7	121	2	4	895	698.87	2.74	922	124.89	6.05	0.178702763
121	26	4	119	9	7	121	2	4	314	712.34	3.94	279	92.86	6.51	0.130359098
121	26	4	119	9	7	121	2	4	895	688.61	2.82	1203	91.16	5.43	0.132382626
121	26	4	119	9	7	121	2	4	902	688.14	2.86	1199	91.31	5.42	0.132691022
121	26	4	119	9	7	121	2	4	465	698.22	3.44	460	94.50	5.50	0.135344161

Table S4. Continued,

C) Mixed-ploidy fertilizations seeds

Pollen recipient			Pollen donor 1			Pollen donor 2			Standard			Embryo			Embryo:Standard ratio
Population	Individual	Ploidy [*x*]	Population	Individual	Ploidy [*x*]	Population	Individual	Ploidy [*x*]	Count	Mean	CV	Count	Mean	CV	
121	27	4	121	6	4	119	49	7	369	689.44	2.65	287	91.92	4.29	0.133325598
121	27	4	121	6	4	119	49	7	547	697.42	3.04	621	96.79	5.38	0.138782943
121	27	4	121	6	4	119	49	7	683	689.34	3.28	645	94.99	5.25	0.137798474
121	27	4	121	6	4	119	49	7	291	689.08	2.87	383	92.69	4.34	0.134512684
121	27	4	121	6	4	119	49	7	805	698.06	3.00	507	93.75	4.72	0.134300776
121	27	4	119	46	7	121	2	4	316	695.80	3.74	1162	96.73	5.60	0.139019833
121	27	4	119	46	7	121	2	4	603	703.79	2.99	774	131.55	4.58	0.186916552
121	27	4	119	46	7	121	2	4	1287	700.55	2.69	1456	95.18	4.85	0.135864678
121	27	4	119	46	7	121	2	4	861	694.81	2.71	414	92.09	4.71	0.132539831
121	27	4	119	46	7	121	2	4	886	711.49	3.27	237	94.91	6.45	0.133396112
121	27	4	119	46	7	121	2	4	1223	692.08	3.15	950	95.47	5.53	0.137946480
121	27	4	119	46	7	121	2	4	999	701.51	2.80	852	99.19	5.74	0.141394991
121	27	4	119	46	7	121	2	4	397	701.61	2.96	584	92.93	4.49	0.132452502
121	27	4	119	46	7	121	2	4	862	684.99	2.91	819	92.90	4.80	0.135622418
121	27	4	119	46	7	121	2	4	1133	688.34	2.70	1029	90.92	5.77	0.132085888
121	27	4	119	46	7	121	2	4	404	699.11	2.79	669	93.54	4.81	0.133798687
121	27	4	121	49	4	119	6	7	412	709.21	2.46	473	99.05	5.89	0.139662441
121	27	4	121	49	4	119	6	7	467	687.31	2.64	1002	93.15	4.94	0.135528364
121	27	4	121	49	4	119	6	7	659	691.62	2.85	984	92.77	4.99	0.134134351
121	27	4	121	49	4	119	6	7	713	694.32	2.91	1525	94.85	5.66	0.136608480
121	27	4	121	49	4	119	6	7	714	699.51	3.05	1503	94.23	4.90	0.134708582
121	30	4	121	12	4	119	22	7	592	712.93	4.15	1303	97.24	5.49	0.136394877
121	30	4	121	12	4	119	22	7	343	695.41	3.35	832	90.52	5.64	0.130167815
121	30	4	121	12	4	119	22	7	472	696.38	3.40	963	109.57	6.27	0.157342256
121	30	4	121	12	4	119	22	7	433	729.04	3.25	857	104.09	5.76	0.142776802
121	30	4	121	12	4	119	22	7	686	693.65	3.62	1736	101.09	8.22	0.145736322
121	30	4	121	12	4	119	22	7	404	709.13	3.09	1314	95.92	5.53	0.135264338
121	30	4	121	12	4	119	22	7	162	693.47	3.31	561	96.24	5.81	0.138780337
121	30	4	121	12	4	119	22	7	1054	702.15	3.29	995	95.70	4.96	0.136295663
121	30	4	121	12	4	119	22	7	296	689.82	3.12	1054	93.12	5.90	0.134991737
121	30	4	121	12	4	119	22	7	125	695.19	4.19	1829	98.80	5.19	0.142119421
121	30	4	121	52	4	119	73	7	515	690.12	3.01	545	96.95	4.57	0.140482815
121	30	4	121	52	4	119	73	7	798	747.24	2.97	624	104.29	4.18	0.139566940
121	30	4	121	52	4	119	73	7	726	701.51	4.30	987	100.77	5.93	0.143647275
121	30	4	121	52	4	119	73	7	1030	690.62	4.05	666	97.63	5.60	0.141365729
121	30	4	121	13	4	119	67	7	939	716.85	3.56	641	101.60	5.02	0.141731185
121	30	4	121	13	4	119	67	7	991	729.19	2.93	1461	101.54	4.23	0.139250401
121	30	4	121	13	4	119	67	7	644	694.81	3.35	548	96.74	5.43	0.139232308

Table S4. Continued,

C) Mixed-ploidy fertilizations seeds

Pollen recipient			Pollen donor 1			Pollen donor 2			Standard			Embryo			Embryo:Standard ratio
Population	Individual	Ploidy [*x*]	Population	Individual	Ploidy [*x*]	Population	Individual	Ploidy [*x*]	Count	Mean	CV	Count	Mean	CV	
121	30	4	121	13	4	119	67	7	908	707.45	5.22	2557	98.72	6.64	0.139543431
121	30	4	121	13	4	119	67	7	524	720.24	3.88	460	99.70	4.56	0.138426080
121	30	4	119	21	7	121	12	4	608	728.26	3.55	1246	101.94	5.76	0.139977481
121	30	4	119	21	7	121	12	4	335	718.43	3.27	779	101.84	5.47	0.141753546
121	30	4	119	21	7	121	12	4	673	700.36	3.08	451	99.15	5.57	0.141570050
121	30	4	119	73	7	121	52	4	526	707.94	2.65	499	98.95	6.01	0.139771732
121	30	4	119	73	7	121	52	4	740	703.19	3.26	413	101.16	3.92	0.143858701
121	30	4	119	73	7	121	52	4	897	708.63	3.22	492	103.10	4.84	0.145492006
121	30	4	119	67	7	121	13	4	357	718.90	2.71	1165	101.06	4.94	0.140575880
121	30	4	119	67	7	121	13	4	359	726.93	3.59	1287	100.56	4.79	0.138335190
121	36	4	121	29	4	119	29	7	306	714.90	3.37	1074	99.47	5.68	0.139138341
121	36	4	121	5	4	119	26	7	437	728.33	3.33	829	103.94	5.39	0.142710035
121	36	4	121	5	4	119	26	7	510	719.96	2.85	766	100.74	4.17	0.139924440
121	36	4	121	5	4	119	26	7	476	721.30	3.25	1214	100.36	5.59	0.139137668
121	36	4	121	5	4	119	26	7	218	687.68	2.74	1231	96.88	4.13	0.140879479
121	36	4	121	5	4	119	26	7	490	712.07	3.16	1282	100.34	5.75	0.140913112
121	36	4	121	2	4	119	64	7	564	713.76	2.83	1120	101.61	4.55	0.142358776
121	36	4	121	2	4	119	64	7	599	704.58	3.36	1280	99.92	5.93	0.141814982
121	36	4	121	2	4	119	64	7	805	713.12	2.78	1450	100.83	4.66	0.141392753
121	36	4	121	2	4	119	64	7	335	734.90	2.96	1175	103.76	5.53	0.141189277
121	36	4	119	29	7	121	29	4	281	713.11	2.89	1047	99.48	6.82	0.139501620
121	36	4	119	29	7	121	29	4	325	723.54	2.73	1488	100.53	4.19	0.138941869
121	36	4	119	29	7	121	29	4	440	730.79	3.13	1120	103.30	5.23	0.141353877
121	36	4	119	29	7	121	29	4	381	720.71	3.07	1196	98.16	4.44	0.136199026
121	36	4	119	26	7	121	5	4	501	726.49	2.67	1741	101.48	4.91	0.139685336
121	36	4	119	26	7	121	5	4	1287	727.61	2.80	1246	98.96	4.24	0.136006927
121	36	4	119	26	7	121	5	4	242	720.04	3.97	432	106.33	6.77	0.147672352
121	36	4	119	26	7	121	5	4	534	702.35	2.96	1667	96.10	5.00	0.136826369
121	36	4	119	64	7	121	12	4	668	699.84	2.57	1081	94.92	4.42	0.135631001
121	36	4	119	64	7	121	12	4	926	724.61	2.82	1305	100.22	4.64	0.138308883
121	36	4	119	64	7	121	12	4	735	702.04	3.08	825	96.70	4.04	0.137741439
121	37	4	121	5	4	119	53	7	741	715.25	3.16	1990	99.13	4.57	0.138594897
121	37	4	121	5	4	119	53	7	325	719.98	2.43	1062	102.98	5.83	0.143031751
121	37	4	121	5	4	119	53	7	339	706.23	3.10	904	97.86	4.71	0.138566756
121	37	4	121	5	4	119	53	7	470	720.91	3.25	1233	99.68	5.30	0.138269687
121	37	4	121	13	4	119	51	7	413	714.98	2.47	2381	99.25	4.53	0.138815072
121	37	4	121	13	4	119	51	7	445	715.21	3.22	385	95.02	7.78	0.132856084
121	37	4	121	13	4	119	51	7	129	694.60	3.27	349	100.01	5.37	0.143982148

Table S4. Continued,

C) Mixed-ploidy fertilizations seeds

Pollen recipient			Pollen donor 1			Pollen donor 2			Standard			Embryo			Embryo:Standard ratio
Population	Individual	Ploidy [*x*]	Population	Individual	Ploidy [*x*]	Population	Individual	Ploidy [*x*]	Count	Mean	CV	Count	Mean	CV	
121	37	4	121	52	4	119	5	7	581	694.07	3.32	681	99.34	5.88	0.143126774
121	37	4	121	52	4	119	5	7	318	701.56	3.14	534	100.34	4.82	0.143024118
121	37	4	121	52	4	119	5	7	1249	694.66	3.07	605	96.54	3.93	0.138974462
121	37	4	119	53	7	119	5	7	440	701.97	2.76	1519	96.49	4.45	0.137456017
121	37	4	119	53	7	119	5	7	723	722.71	2.63	699	130.60	6.63	0.180708721
121	37	4	119	53	7	119	5	7	305	723.87	2.99	1036	98.26	3.97	0.135742606
121	37	4	119	51	7	121	13	4	388	709.46	2.51	365	96.77	5.95	0.136399515
121	37	4	119	51	7	121	13	4	336	691.62	2.68	503	94.02	3.94	0.135941702
121	37	4	119	51	7	121	13	4	336	700.34	2.45	1080	95.28	4.20	0.136048205
121	37	4	119	51	7	121	13	4	486	714.18	3.39	1690	100.96	5.14	0.141364922
121	43	4	121	12	4	119	16	7	249	715.84	2.33	658	102.25	5.03	0.142839182
121	43	4	121	12	4	119	16	7	490	718.27	2.28	1187	102.37	4.74	0.142523007
121	43	4	121	12	4	119	16	7	594	711.84	3.27	1069	97.55	4.39	0.137039222
121	43	4	119	16	7	121	12	4	414	689.84	2.78	677	96.77	5.31	0.140278905
121	43	4	119	16	7	121	12	4	808	696.18	2.90	1397	98.67	4.58	0.141730587
121	43	4	119	16	7	121	12	4	1223	703.84	2.67	554	107.76	5.70	0.153102978
121	43	4	119	16	7	121	12	4	1125	710.69	2.77	573	100.07	5.47	0.140806822
144	29	4	144	8	4	144	2	5	774	703.94	2.85	2502	97.23	5.60	0.138122567
144	29	4	144	8	4	144	2	5	709	715.94	3.49	1397	102.12	4.82	0.142637651
144	29	4	144	8	4	144	2	5	1737	712.44	3.14	1269	100.70	5.06	0.141345236
144	29	4	144	8	4	144	2	5	1249	737.96	3.25	1449	124.80	4.35	0.169114857
144	29	4	144	8	4	144	2	5	868	684.29	3.07	1104	92.56	6.04	0.135264289
144	29	4	144	8	4	144	2	5	1087	714.19	3.14	1788	98.40	6.54	0.137778462
144	29	4	144	8	4	144	2	5	2186	687.71	2.88	424	89.45	4.98	0.130069361
144	29	4	144	8	4	144	2	5	1341	741.00	2.95	1657	112.88	5.63	0.152334683
144	29	4	144	8	4	144	2	5	1622	716.99	2.55	726	97.78	4.91	0.136375682
144	29	4	144	8	4	144	2	5	1046	715.02	3.07	914	97.66	4.46	0.136583592
144	29	4	144	8	4	144	2	5	1932	704.50	3.45	602	94.09	4.31	0.133555713
144	31	4	144	8	4	144	26	5	855	766.93	2.85	2012	106.14	4.24	0.138395942
144	31	4	144	8	4	144	26	5	1529	720.42	2.51	1469	98.05	4.23	0.136101163
144	31	4	144	8	4	144	26	5	1180	723.81	3.24	1672	98.33	4.83	0.135850569
144	31	4	144	6	4	144	20	5	2056	714.99	3.16	1366	100.59	4.31	0.140687282
144	31	4	144	4	4	144	5	5	1622	754.25	2.91	1940	111.45	4.87	0.147762678
144	31	4	144	4	4	144	5	5	1048	714.60	2.61	946	122.52	4.59	0.171452561
144	31	4	144	4	4	144	5	5	1262	743.22	2.80	1504	131.81	4.15	0.177349910
144	31	4	144	4	4	144	5	5	1153	710.74	3.05	1514	97.25	5.21	0.136829220
144	31	4	144	4	4	144	5	5	1120	714.45	2.78	883	95.38	5.62	0.133501295
144	31	4	144	26	5	144	8	4	1915	762.85	2.68	1788	106.62	4.72	0.139765354

Table S4. Continued,

C) Mixed-ploidy fertilizations seeds															
Pollen recipient			Pollen donor 1			Pollen donor 2			Standard			Embryo			Embryo:Standard ratio
Population	Individual	Ploidy [*x*]	Population	Individual	Ploidy [*x*]	Population	Individual	Ploidy [*x*]	Count	Mean	CV	Count	Mean	CV	
144	31	4	144	10	5	144	6	4	1523	737.71	2.88	671	101.60	4.31	0.137723496
144	31	4	144	10	5	144	6	4	1409	726.51	2.84	700	97.49	4.75	0.134189481
144	31	4	144	10	5	144	6	4	1399	735.19	2.60	1156	100.79	4.52	0.137093812
144	31	4	144	10	5	144	6	4	1410	717.25	2.49	1070	97.99	5.93	0.136619031
144	31	4	144	10	5	144	6	4	1406	709.52	3.69	1515	119.82	5.03	0.168874732
144	31	4	144	5	5	144	4	4	1537	705.53	2.85	1700	97.65	4.72	0.138406588
144	31	4	144	5	5	144	4	4	2462	700.52	2.59	1160	95.29	4.91	0.136027522
144	31	4	144	5	5	144	4	4	1866	728.76	3.69	1485	100.69	4.90	0.138166200
144	31	4	144	5	5	144	4	4	1556	704.24	2.63	1089	95.54	5.41	0.135663978
144	31	4	144	5	5	144	4	4	942	718.88	3.75	418	120.14	4.90	0.167121077
144	33	4	144	25	5	144	20	4	1967	708.40	2.83	869	116.85	4.81	0.164949181
144	33	4	144	25	5	144	20	4	2097	707.41	2.60	594	96.41	4.35	0.136285888
144	33	4	144	25	5	144	20	4	1761	726.22	3.14	1618	107.85	5.22	0.148508716
144	33	4	144	25	5	144	20	4	2182	719.61	3.35	761	98.00	5.57	0.136184878
144	33	4	144	25	5	144	20	4	1098	705.18	2.58	1244	96.06	7.16	0.136220539
144	33	4	144	25	5	144	20	4	1252	718.77	2.84	1428	100.58	4.54	0.139933498
144	33	4	144	96	5	144	21	4	1250	694.08	3.01	1655	99.37	5.17	0.143167935
144	33	4	144	96	5	144	21	4	2055	724.97	3.00	901	101.58	5.57	0.140116143
144	33	4	144	96	5	144	21	4	364	725.21	3.78	843	99.71	5.16	0.137491209
144	33	4	144	96	5	144	21	4	731	733.76	3.24	776	103.48	4.43	0.141027039
144	33	4	144	27	5	144	22	4	1553	724.30	3.50	693	108.53	4.68	0.149841226
144	33	4	144	27	5	144	22	4	777	731.91	2.95	1081	101.83	5.56	0.139129128
144	33	4	144	27	5	144	22	4	1525	724.33	3.06	1649	102.65	4.93	0.141717173
144	33	4	144	27	5	144	22	4	796	705.09	2.71	1281	98.02	5.29	0.139017714
144	33	4	144	27	5	144	22	4	1250	715.34	3.05	1201	100.57	4.89	0.140590488
144	33	4	144	27	5	144	22	4	720	717.66	2.86	1453	100.77	4.90	0.140414681
144	33	4	144	27	5	144	22	4	916	695.43	2.59	1419	124.98	5.03	0.179716147
144	37	4	144	77	4	144	2	5	1311	709.32	3.40	2242	99.62	5.08	0.140444369
144	37	4	144	77	4	144	2	5	1758	728.62	3.38	1556	101.57	4.89	0.139400511
144	37	4	144	30	4	144	99	5	1183	729.66	2.71	1873	102.65	4.65	0.140681961
144	37	4	144	30	4	144	99	5	865	722.24	3.40	910	104.79	5.28	0.145090275
144	37	4	144	30	4	144	99	5	1510	710.34	2.89	1737	125.11	4.72	0.176126925
144	37	4	144	50	4	144	10	5	1330	734.10	3.06	468	101.08	3.92	0.137692412
144	37	4	144	50	4	144	10	5	784	709.85	2.65	1474	117.20	4.35	0.165105304
144	37	4	144	50	4	144	10	5	664	716.66	3.12	1736	99.06	5.35	0.138224542
144	37	4	144	50	4	144	10	5	448	720.84	3.04	688	99.21	3.87	0.137631097
144	37	4	144	2	5	144	77	4	1236	689.20	4.28	2319	96.57	5.60	0.140118979
144	37	4	144	2	5	144	77	4	716	712.56	2.95	1307	100.87	4.79	0.141560009

Table S4. Continued,

C) Mixed-ploidy fertilizations seeds

Pollen recipient			Pollen donor 1			Pollen donor 2			Standard			Embryo			Embryo:Standard ratio
Population	Individual	Ploidy [*x*]	Population	Individual	Ploidy [*x*]	Population	Individual	Ploidy [*x*]	Count	Mean	CV	Count	Mean	CV	
144	37	4	144	2	5	144	77	4	1108	683.06	3.45	1367	95.85	6.61	0.140324422
144	37	4	144	2	5	144	77	4	1192	687.85	3.55	1497	96.58	4.74	0.140408519
144	37	4	144	99	5	144	30	4	617	712.90	3.31	755	104.66	5.18	0.146808809
144	37	4	144	99	5	144	30	4	728	674.75	3.31	1233	92.72	4.37	0.137413857
144	37	4	144	99	5	144	30	4	664	732.66	2.84	1275	102.19	5.27	0.139478066
144	37	4	144	10	5	144	50	4	513	686.32	3.37	2220	95.29	4.73	0.138841940
144	37	4	144	10	5	144	50	4	466	705.16	3.33	807	97.00	4.50	0.137557434
144	37	4	144	10	5	144	50	4	1027	697.76	3.13	1978	98.68	4.72	0.141423985
144	37	4	144	10	5	144	50	4	1180	701.92	3.21	1123	98.69	4.74	0.140600068
144	47	4	144	9	4	144	27	5	1267	690.04	2.90	1379	95.37	4.95	0.138209379
144	47	4	144	9	4	144	27	5	1025	725.21	2.81	1442	101.36	4.98	0.139766412
144	47	4	144	9	4	144	27	5	1121	695.71	2.94	1270	96.60	4.88	0.138850958
144	47	4	144	9	4	144	27	5	883	709.15	3.11	1267	98.43	5.66	0.138799972
144	47	4	144	9	4	144	27	5	1236	718.98	3.09	1199	101.35	5.94	0.140963587
144	47	4	144	9	4	144	27	5	344	713.62	2.45	1105	100.16	3.71	0.140354811
144	47	4	144	18	4	144	15	5	1747	689.14	3.03	1815	97.30	4.65	0.141190469
144	47	4	144	18	4	144	15	5	806	706.61	2.73	1544	100.34	5.11	0.142001953
144	47	4	144	18	4	144	15	5	1482	700.69	3.14	656	96.72	5.28	0.138035365
144	47	4	144	30	4	144	69	5	225	699.28	3.05	711	96.85	4.48	0.138499600
144	47	4	144	30	4	144	69	5	994	703.01	2.80	1540	99.62	6.03	0.141704954
144	47	4	144	30	4	144	69	5	487	719.02	2.91	861	100.77	5.22	0.140149092
144	47	4	144	30	4	144	69	5	1573	715.60	2.89	1076	97.59	4.98	0.136375070
144	47	4	144	27	5	144	9	4	1503	701.83	3.31	1187	98.14	5.14	0.139834433
144	47	4	144	27	5	144	9	4	454	724.37	2.78	182	98.26	4.01	0.135648909
144	47	4	144	27	5	144	9	4	952	722.64	2.73	1976	101.85	5.06	0.140941548
144	47	4	144	27	5	144	9	4	775	733.63	3.91	1017	102.94	4.53	0.140315963
144	47	4	144	15	5	144	18	4	1420	698.01	3.49	1469	97.83	5.30	0.140155585
144	47	4	144	15	5	144	18	4	1525	723.05	2.77	1755	100.99	4.47	0.139672222
144	47	4	144	15	5	144	18	4	1615	702.97	2.98	1424	100.35	5.14	0.142751469
144	47	4	144	15	5	144	18	4	1404	728.24	2.67	1166	102.74	4.27	0.141079864
144	47	4	144	15	5	144	18	4	460	741.68	3.26	708	105.90	4.01	0.142783950
144	47	4	144	15	5	144	18	4	1822	720.44	3.44	1094	102.96	4.84	0.142912664
144	47	4	144	15	5	144	18	4	1266	729.37	3.41	1066	101.97	5.22	0.139805586
144	49	4	144	50	4	144	96	5	813	718.34	2.60	1360	99.49	5.41	0.138499875
144	49	4	144	50	4	144	96	5	424	695.94	2.92	814	97.26	5.46	0.139753427
144	49	4	144	50	4	144	96	5	417	706.29	3.28	996	100.41	5.21	0.142165399
144	49	4	144	50	4	144	96	5	479	706.17	2.92	956	100.61	5.01	0.142472776
144	49	4	144	37	4	144	100	5	802	714.11	2.57	611	99.71	5.33	0.139628349

Table S4. Continued,

C) Mixed-ploidy fertilizations seeds

Pollen recipient			Pollen donor 1			Pollen donor 2			Standard			Embryo			Embryo:Standard ratio
Population	Individual	Ploidy [*x*]	Population	Individual	Ploidy [*x*]	Population	Individual	Ploidy [*x*]	Count	Mean	CV	Count	Mean	CV	
144	49	4	144	47	4	144	15	5	1756	701.53	2.99	1168	98.54	5.01	0.140464413
144	49	4	144	47	4	144	15	5	1710	701.80	2.76	1223	98.33	5.45	0.140111143
144	49	4	144	96	5	144	50	4	1548	722.38	2.74	1620	100.99	4.97	0.139801766
144	49	4	144	96	5	144	50	4	828	716.37	2.53	1432	99.91	6.34	0.139467035
144	49	4	144	96	5	144	50	4	236	718.94	2.43	557	99.75	5.42	0.138745932
144	49	4	144	100	5	144	37	4	1893	681.55	2.98	1222	93.26	5.36	0.136835155
144	49	4	144	100	5	144	37	4	1701	699.15	2.76	1859	99.55	6.33	0.142387184
144	49	4	144	100	5	144	37	4	2139	716.24	2.45	1095	99.13	4.44	0.138403328
144	49	4	144	15	5	144	47	4	1046	707.31	2.80	495	98.45	4.34	0.139189323
144	49	4	144	15	5	144	47	4	1852	712.32	2.58	1061	98.51	4.24	0.138294587
144	49	4	144	15	5	144	47	4	1114	712.55	2.45	1193	100.24	4.56	0.140677847
144	50	4	144	77	4	144	100	5	902	711.89	2.51	1206	100.19	3.81	0.140738035
144	50	4	144	77	4	144	100	5	852	725.64	2.51	731	100.21	4.61	0.138098782
144	50	4	144	77	4	144	100	5	623	735.59	2.75	1042	101.15	4.63	0.137508667
144	50	4	144	77	4	144	100	5	417	720.77	2.13	980	100.90	4.23	0.139989178
144	50	4	144	77	4	144	100	5	696	724.14	2.36	1596	102.86	4.79	0.142044356
144	50	4	144	37	4	144	35	5	500	693.52	2.61	1832	112.12	4.79	0.161668012
144	50	4	144	37	4	144	35	5	1320	708.30	2.41	2090	99.46	5.11	0.140420726
144	50	4	144	37	4	144	35	5	882	737.66	2.60	940	100.96	5.08	0.136865222
144	50	4	144	37	4	144	35	5	702	717.57	2.99	1491	100.61	4.88	0.140209318
144	50	4	144	49	4	144	25	5	1006	742.51	3.04	1248	102.54	5.70	0.138099150
144	50	4	144	49	4	144	25	5	669	714.78	2.50	1712	104.49	4.56	0.146184840
144	50	4	144	49	4	144	25	5	1296	753.81	2.63	1401	105.37	4.85	0.139783235
144	50	4	144	100	5	144	77	4	645	712.54	2.51	2306	100.76	5.11	0.141409605
144	50	4	144	100	5	144	77	4	564	737.97	3.05	1164	103.32	5.32	0.140005691
144	50	4	144	35	5	144	37	4	695	737.89	3.05	1971	120.18	5.49	0.162869804
144	50	4	144	35	5	144	37	4	493	740.60	3.37	626	101.71	5.32	0.137334594
144	50	4	144	25	5	144	49	4	604	714.07	2.85	1990	99.69	5.69	0.139608162
144	50	4	144	25	5	144	49	4	659	716.03	2.77	1592	98.83	5.31	0.138024943
144	50	4	144	25	5	144	49	4	504	727.28	2.74	1812	102.43	5.19	0.140839842
144	50	4	144	25	5	144	49	4	1040	711.34	3.16	1034	97.50	5.09	0.137065257
144	51	4	144	4	4	144	26	5	1360	731.38	3.33	777	101.63	4.87	0.138956493
144	51	4	144	6	4	144	15	5	591	729.18	3.84	915	99.94	4.95	0.137058065
144	51	4	144	6	4	144	15	5	878	748.54	3.74	1085	100.63	5.50	0.134435034
144	51	4	144	6	4	144	15	5	598	744.77	2.71	1714	103.08	4.84	0.138405145
144	51	4	144	6	4	144	15	5	738	780.42	3.26	649	106.00	4.74	0.135824300
144	51	4	144	26	5	144	4	4	464	754.09	2.89	1755	104.22	4.94	0.138206315
144	51	4	144	26	5	144	4	4	800	774.07	3.58	1220	106.82	4.70	0.137997855

Table S4. Continued,

C) Mixed-ploidy fertilizations seeds

Pollen recipient			Pollen donor 1			Pollen donor 2			Standard			Embryo			Embryo:Standard ratio
Population	Individual	Ploidy [*x*]	Population	Individual	Ploidy [*x*]	Population	Individual	Ploidy [*x*]	Count	Mean	CV	Count	Mean	CV	
144	51	4	144	26	5	144	4	4	872	721.46	3.08	578	98.07	5.38	0.135932692
144	51	4	144	97	5	144	18	4	370	755.90	2.72	818	103.49	5.28	0.136909644
144	51	4	144	97	5	144	18	4	573	696.09	2.67	1574	96.77	6.50	0.139019380
144	51	4	144	97	5	144	18	4	398	711.21	2.94	653	96.32	5.71	0.135431167
144	56	4	144	28	4	144	97	5	178	723.16	2.86	2760	102.63	5.24	0.141918801
144	56	4	144	28	4	144	97	5	562	721.62	4.14	602	96.04	6.39	0.133089438
144	56	4	144	28	4	144	97	5	162	753.46	2.85	1125	103.91	5.19	0.137910440
144	56	4	144	28	4	144	97	5	537	709.81	3.37	1180	97.98	4.75	0.138036939
144	56	4	144	29	4	144	2	5	594	699.81	3.27	1248	96.66	5.00	0.138123205
144	56	4	144	47	4	144	99	5	1193	730.86	2.73	1340	100.33	5.44	0.137276633
144	56	4	144	97	5	144	28	4	788	721.20	2.92	1456	100.07	5.82	0.138754853
144	56	4	144	97	5	144	28	4	1199	712.47	2.54	1336	98.23	5.46	0.137872472
144	56	4	144	97	5	144	28	4	645	727.31	2.38	829	98.62	4.12	0.135595551
144	56	4	144	2	5	144	29	4	1944	719.52	2.70	1024	100.03	4.79	0.139023238
144	56	4	144	2	5	144	29	4	1834	718.72	2.62	1102	99.96	4.62	0.139080588
144	56	4	144	2	5	144	29	4	1273	745.88	2.78	1461	103.90	5.24	0.139298547
144	56	4	144	2	5	144	29	4	1375	736.30	2.93	562	100.52	4.69	0.136520440
144	56	4	144	2	5	144	29	4	1396	698.63	3.27	1130	96.09	5.93	0.137540615
144	56	4	144	99	5	144	47	4	994	741.68	2.59	1348	101.91	4.46	0.137404271
144	56	4	144	99	5	144	47	4	1294	694.67	2.75	981	93.66	5.05	0.134826608
144	56	4	144	99	5	144	47	4	2512	748.17	2.67	1054	102.87	4.83	0.137495489
144	56	4	144	99	5	144	47	4	1216	720.37	2.61	1143	98.35	5.75	0.136527062
144	57	4	144	77	4	144	27	5	1445	736.35	2.91	1806	101.93	5.33	0.138426020
144	57	4	144	77	4	144	27	5	914	711.67	2.79	1133	97.24	5.38	0.136636362
144	57	4	144	77	4	144	27	5	1969	727.32	2.50	1347	101.07	3.88	0.138962217
144	57	4	144	77	4	144	27	5	756	717.27	2.67	690	96.03	4.52	0.133882638
144	57	4	144	9	4	144	5	5	789	739.60	3.29	1293	102.63	5.68	0.138764197
144	57	4	144	9	4	144	5	5	627	703.28	2.60	1700	95.22	5.69	0.135394153
144	57	4	144	9	4	144	5	5	801	725.28	2.69	900	102.46	4.43	0.141269579
144	57	4	144	9	4	144	5	5	1104	722.19	2.85	1050	97.76	5.48	0.135366039
144	57	4	144	27	5	144	77	4	1148	728.31	3.15	1325	100.85	4.96	0.138471255
144	57	4	144	5	5	144	9	4	583	699.80	2.46	1460	98.13	5.07	0.140225779
144	57	4	144	5	5	144	9	4	694	724.05	2.65	669	99.02	4.83	0.136758511
144	57	4	144	5	5	144	9	4	807	727.06	2.72	1669	101.26	5.00	0.139273237
144	58	4	144	21	4	144	100	5	919	721.95	2.91	1843	101.08	5.30	0.140009696
144	58	4	144	21	4	144	100	5	943	710.45	2.34	1057	96.81	4.97	0.136265747
144	58	4	144	21	4	144	100	5	1146	707.15	2.94	1644	96.24	4.98	0.136095595
144	58	4	144	21	4	144	100	5	878	700.46	2.87	851	96.77	5.75	0.138152071

Table S4. Continued,

C) Mixed-ploidy fertilizations seeds

Pollen recipient			Pollen donor 1			Pollen donor 2			Standard			Embryo			Embryo:Standard ratio
Population	Individual	Ploidy [*x*]	Population	Individual	Ploidy [*x*]	Population	Individual	Ploidy [*x*]	Count	Mean	CV	Count	Mean	CV	
144	58	4	144	29	4	144	96	5	915	737.09	2.95	842	122.87	4.34	0.166696062
144	58	4	144	29	4	144	96	5	997	698.67	2.72	1498	94.97	5.07	0.135929695
144	58	4	144	29	4	144	96	5	681	686.25	3.07	879	92.75	5.85	0.135154827
144	58	4	144	100	5	144	21	4	952	693.66	2.91	1683	92.63	5.21	0.133538045
144	58	4	144	100	5	144	21	4	1164	699.08	2.96	1357	97.51	4.78	0.139483321
144	58	4	144	100	5	144	21	4	1252	717.56	3.05	1032	100.34	4.49	0.139834996
144	58	4	144	100	5	144	21	4	563	707.48	3.94	830	94.96	5.73	0.134222876
144	58	4	144	100	5	144	21	4	582	705.22	3.26	1138	93.90	5.64	0.133149939
144	58	4	144	100	5	144	21	4	379	697.34	2.95	1416	94.19	6.13	0.13507041
144	60	4	144	9	4	144	35	5	1207	712.34	5.75	1275	102.75	5.62	0.144242918
144	60	4	144	9	4	144	35	5	540	710.22	3.35	1459	95.99	6.40	0.135155304
144	60	4	144	9	4	144	35	5	1205	724.33	3.39	1795	120.96	5.66	0.166995706
144	60	4	144	9	4	144	35	5	768	696.06	3.46	1278	93.55	6.28	0.134399333
144	60	4	144	22	4	144	25	5	2334	723.07	2.81	849	98.20	5.49	0.135809811
144	60	4	144	22	4	144	25	5	425	698.28	2.68	782	94.29	5.98	0.135031792
144	60	4	144	22	4	144	25	5	1561	708.35	2.56	1597	97.79	4.97	0.138053222
144	60	4	144	22	4	144	25	5	753	703.84	2.69	1660	97.13	6.37	0.138000114
144	60	4	144	47	4	144	97	5	669	728.10	3.17	535	101.21	5.40	0.139005631
144	60	4	144	47	4	144	97	5	845	710.17	3.46	1034	97.44	5.79	0.137206584
144	60	4	144	47	4	144	97	5	696	717.74	2.40	343	100.75	5.17	0.140371165
144	60	4	144	47	4	144	97	5	608	711.17	2.91	785	97.04	5.78	0.1364512
144	60	4	144	35	5	144	9	4	955	708.16	2.40	1708	98.09	5.33	0.138513895
144	60	4	144	35	5	144	9	4	634	707.87	2.76	1094	97.08	5.40	0.137143826
144	60	4	144	25	5	144	22	4	1361	716.63	3.01	769	98.73	5.04	0.137769839
144	60	4	144	25	5	144	22	4	358	716.32	3.48	999	96.87	6.18	0.135232857
144	60	4	144	25	5	144	22	4	1363	700.28	2.65	1619	95.24	4.63	0.136002742
144	60	4	144	97	5	144	47	4	2006	714.31	2.87	1295	98.48	5.05	0.137867313
144	60	4	144	97	5	144	47	4	313	699.04	3.12	256	93.17	5.17	0.133282788
144	60	4	144	97	5	144	47	4	977	721.30	3.38	807	100.19	5.36	0.138901983
144	60	4	144	97	5	144	47	4	250	707.15	4.14	798	98.54	5.57	0.139348087
